# A Study on the Effect of Quaternization of Polyene Antibiotics’ Structures on Their Activity, Toxicity, and Impact on Membrane Models

**DOI:** 10.3390/antibiotics13070608

**Published:** 2024-06-29

**Authors:** Olga Omelchuk, Anna Tevyashova, Svetlana Efimova, Natalia Grammatikova, Elena Bychkova, George Zatonsky, Lyubov Dezhenkova, Nikita Savin, Svetlana Solovieva, Olga Ostroumova, Andrey Shchekotikhin

**Affiliations:** 1 Gause Institute of New Antibiotics, 11 B. Pirogovskaya, Moscow 119021, Russiagzatonsk@gmail.com (G.Z.);; 2 School of Science, Constructor University, Campus Ring 1, 28759 Bremen, Germany; 3 Institute of Cytology, The Russian Academy of Sciences, 4 Tikhoretsky Ave., St. Petersburg 194064, Russia; ssefimova@mail.ru (S.E.); osostroumova@mail.ru (O.O.); 4 Research Laboratory of Biophysics, National University of Science and Technology “MISIS”, 4 p.1 Leninsky Pr., Moscow 119049, Russia

**Keywords:** polyene antimycotics, drug discovery, semisynthetic derivatives, antifungal activity, antibiofilm activity, scanning ion conductance microscopy, lipid bilayers, hemolysis, calcein leakage

## Abstract

Polyene antibiotics have been used in antifungal therapy since the mid-twentieth century. They are highly valued for their broad spectrum of activity and the rarity of pathogen resistance to their action. However, their use in the treatment of systemic mycoses often results in serious side-effects. Recently, there has been a renewed interest in the development of new antifungal drugs based on polyenes, particularly due to the emergence of highly dangerous pathogenic strains of fungi, such as *Candida auris*, and the increased incidence of mucormycosis. Considerable understanding has been established regarding the structure–biological activity relationships of polyene antifungals. Yet, no previous studies have examined the effect of introducing quaternized fragments into their molecular structure. In this study, we present a series of amides of amphotericin B, nystatin, and natamycin bearing a quaternized group in the side chain, and discuss their biological properties: antifungal activity, cytotoxicity, and effects on lipid bilayers that mimic fungal and mammalian cell membranes. Our research findings suggest that the nature of the introduced quaternized residue plays a more significant role than merely the introduction of a constant positive charge. Among the tested polyenes, derivatives **4b**, **5b**, and **6b**, which contain a fragment of *N*-methyl-4-(aminomethyl)pyridinium in their structure, are particularly noteworthy due to their biological activity.

## 1. Introduction

**Figure 1:**
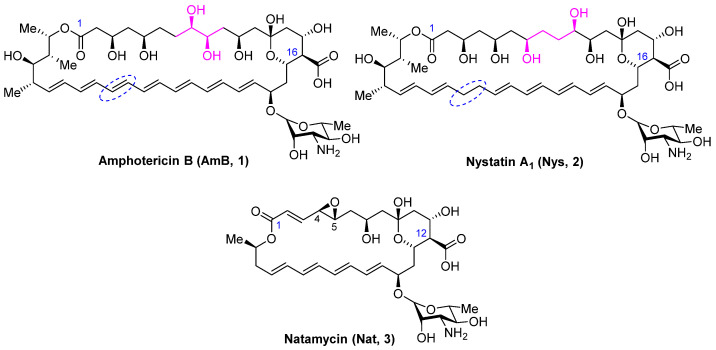
Structure of polyene antifungals Amphotericin B (**1**), Nystatin A_1_ (**2**), and Natamycin (**3**).

Systemic mycoses pose a serious risk, particularly to patients with compromised immunity. First-line antifungal drugs used for the treatment of nosocomial mycoses include azoles (fluconazole, itraconazole, voriconazole, etc.) and echinocandins (micafungin, caspofungin, anidulafungin) [1]. However, the prevalent use of these antifungal agents has led to widespread resistance to these classes of drugs [2,3]. For the treatment of mucormycosis, a life-threatening invasive fungal infection, which, although once considered rare, has become increasingly prevalent in patients affected by SARS-CoV-2, the polyene antibiotic amphotericin B (**1**) (Figure 1) is recommended as the drug of choice [4]. Despite over sixty years of clinical use, amphotericin B remains the gold standard for treating severe fungal infections due to its broad spectrum of antifungal activity and rare instances of clinically significant drug resistance [5]. The systemic use of amphotericin B is limited by its low solubility in aqueous media and serious side-effects such as hemolytic toxicity and nephrotoxicity. The less common polyene antifungal nystatin (**2**) (Figure 1), which differs from amphotericin B in the structure of the polyol region and the absence of a double bond at C28-C29, is usually administered orally or topically. However, it is poorly absorbed from the gastrointestinal tract or through the skin and mucous membranes. Another commercially effective polyene antimycotic, natamycin (**3**) (Figure 1), is in high demand in medicine, agriculture, and the food industry. Its widespread use has also not led to a significant increase in resistance to polyenes [6]. Natamycin (**3**), however, is prescribed only topically due to extremely low bioavailability, so its use for the treatment of systemic mycoses is limited. Recent publications have confirmed a high level of scientific interest in this class of antifungals for the search for a new generation of antifungals [7,8,9].

Polyene macrolides share a common structural feature—a 20–44-membered macrolactone ring containing 4–7 conjugated double bonds. The main structural features of these antifungals, which determine their physicochemical and biological properties, are the amphoteric nature of the molecule and the zwitterionic character—the carboxyl group carries a negative charge, and the amino group of mycosamine is positively charged.

The primary mechanism of action for polyene antifungals is believed to be disruption of the normal function of the cell membrane due to interaction with ergosterol (Erg) (in fungal cells) or cholesterol (Chol) (in mammalian cells) and pore-forming activity in the case of AmB and nystatin [10]. In contrast, natamycin with a smaller macrolactone ring (26 atoms in the ring compared to 38 atoms in the case of AmB and Nys) does not form pores in the membranes of fungal cells, and its antifungal effect is based only on specific binding to ergosterol [11]. Natamycin also inhibits sterol-dependent transport of amino acids and sugars [12]. A group of researchers led by Martin D. Burke hypothesized that the ability of polyenes to interact with Erg is mandatory for antifungal activity, while the formation of transmembrane channels is a secondary effect that increases the activity of amphotericin B and nystatin [13,14]. They also reported that eliminating the capacity to form ion channels while preserving the capacity to bind ergosterol in the case of amphotericin B is a promising approach to reduce its toxicity and increase its effectiveness [15].

Among all the modifications undertaken, the following transformations have significantly improved the pharmacological properties of polyenes: (1) removal of the C2’-OH and C35-OH groups in AmB [16,17], (2) introduction of bulky substituents into the mycosamine group [18,19,20], (3) synthesis of urea derivatives [21], and (4) introduction of an additional basic residue through amidation of the carboxyl group [22,23,24]. The reduction in toxicity of semi-synthetic and synthetic derivatives may be attributed to a decreased affinity for cholesterol (C2’-deoxyAmB) or a loss of pore-forming ability (C35-deoxyAmB) in the first instance. In subsequent cases, the disruption of the ‘salt bridge’ between the carboxyl group of the polyene core and the amino group of the sugar residue, as well as the loss of zwitterionic nature, may be the explanation of the more favorable activity/toxicity ratio. Additionally, it has been observed that the ability to form a positive charge on the mycosamine residue is critical for exhibiting antifungal activity [18,25]. However, to date, no one has focused on the investigation of the effect of introducing a cationic group with constant positive charge to the structure of polyenes.

In this study, we synthesized a series of amides of polyene antimycotics, including amphotericin B, nystatin, and natamycin bearing a quaternized group in the side chain. Our aim was to examine their antifungal activity, cytotoxicity toward human cells, and the ability to increase permeability of Erg- and Chol-containing lipid bilayers mimicking fungal and human cell membranes, respectively.

## 2. Results

### 2.1. Synthesis of Polyene Amides

**Scheme 1:**
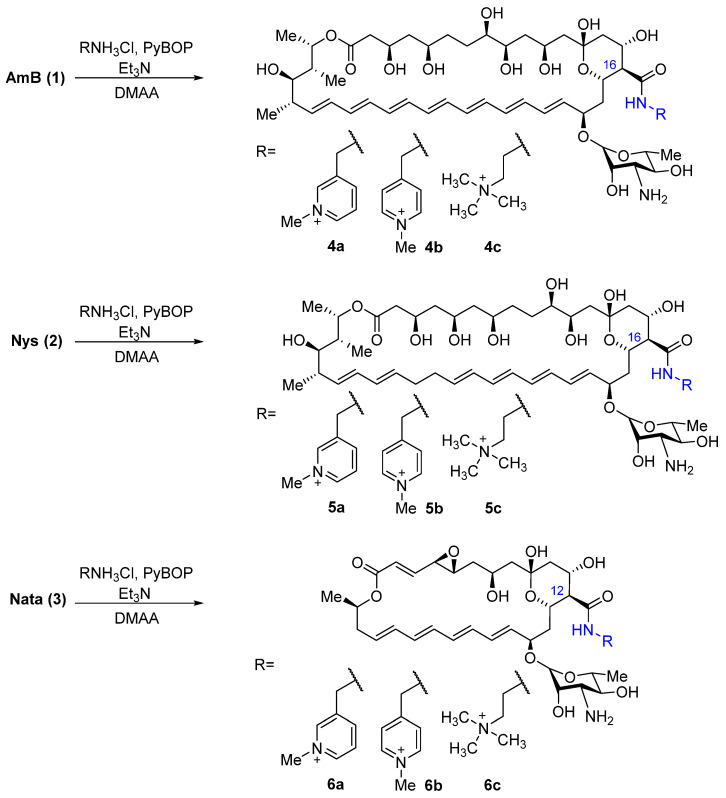
Preparation of amides of AmB (**4a**-**4c**), Nys (**5a**-**5c**), and Nata (**6a**-**6c**) bearing a quaternized group.

The choice of target compounds was based on previously obtained data on the structure–activity relationship. Thus, it was previously shown that *N*-(2-aminoethyl)amides of all three polyene antifungals have improved properties in comparison with the parent compounds [22,24]; in addition, *N*-(2-dimethylaminoethyl)amide and picolylamides of amphotericin B also showed high antifungal activity [26]. Thus, 3- and 4-picolylamines and *N*,*N*,-dimethylethylenediamine quaternized with a methyl group were selected as the introduced fragments. To obtain the target amides of polyene antimycotics, amphotericin B, nystatin, and natamycin were condensed with 3- and 4-(aminomethyl)-1-methylpiridinium hydrochlorides, synthesized from corresponding picolylamines according to a previously published method [27] and trimethyl-(2-aminoethyl)ammonium hydrochloride, synthesized from *N*,*N*-dimethylethylenediamine by the method described in [28]. The reactions with polyene were carried out in dry DMAA with 5 eq. of the corresponding amine hydrochlorides and 5.5 eq. triethylamine to maintain the optimal pH of the reaction. Benzotriazole-1-iloxitripyrrolidinophosphonium hexafluorophosphate (PyBOP) was used as a condensing agent for the coupling of polyene with amino components (Scheme 1).

Purification of the target amides of polyene antifungals with quaternized groups was carried out by reverse-phase chromatography on Snap Sfar C18 silica gel cartridges using the Isolera Prime automatic chromatography system. The purity of new amides **4a**-**4c**, **5a**-**5c,** and **6a**-**6c** was monitored by TLC and determined by HPLC, and the structure was confirmed by HR-ESI mass-spectrometry and NMR spectra. To fully assign signals in both ^1^H and ^13^C spectra, a set of 2D experiments was performed: ^1^H-^1^H COSY, TOCSY, NOESY (ROESY), HSQC, HMBC, and H2BC. The latter experiment was especially useful for assigning resonances from the polyene part of the molecules. In addition, a set of selective TOCSY experiments was performed in order to assign the signals of the H-19, H-20, H-33, and H-34 atoms of the polyene part. The assignment of ^1^H and ^13^C signals for compounds **4a**-**4c**, **5a**-**5c,** and **6a**-**6c** are shown in Tables S1 and S2 (Supplementary Materials).

### 2.2. Spectrum of Antifungal Activity of Quaternized Semisynthetic Polyenes

The antifungal activity of polyene derivatives **4a**-**4c**, **5a**-**5c,** and **6a**-**6c** was tested against strains of *Candida* spp. (*C. albicans* ATCC 10231 and clinical isolates *C. parapsilosis* 58L, *C. albicans* 604M, *C. albicans* 8R, *C. glabrata* 61L, *C. tropicalis* 3010, *C. krusei* 432M) and *Aspergillus fumigatus* ATCC 46645 and compared to that of AmB (**1**), Nys (**2**), and Nata (**3**) (Table 1). Reference strain *C. parapsilopsis* ATCC 22019 was used as the control in each experiment. In all experiments, the in vitro broth microdilution technique in 96-microwell plates was applied as described in the EUCAST Definitive documents [29,30]. The minimum inhibitory concentration (MIC) was defined as the lowest concentration that resulted in complete growth inhibition after incubation for 24 and 48 h for *Candida* spp. and 48–72 h for *A. fumigatus*.

Among the picolylamide isomers, *N*-((1-methylpyridin-1-ium-4-yl)methyl)amides **4b**, **5b**, and **6b** showed the highest activity for all tested polyene types (AmB, Nys, Nata). The activity of *N*-(2-(trimethylammonio)ethyl)amide of AmB (**4c**) was close to that of the parent antifungal and the corresponding amides of nystatin and natamycin showed slightly reduced activity.

### 2.3. Cytoxicity Assays

Preliminary evaluation of the cytotoxicity of new polyene amides was performed using the MTT test on human embryonic kidney cells (HEK293) and human skin fibroblast cells hFB-hTERT6 (Table 2). The reference drugs used were amphotericin B (**1**), nystatin (**2**), natamycin (**3**), and the antitumor antibiotic doxorubicin. 

All quaternized derivatives of nystatin and natamycin demonstrated reduced cytotoxicity. It is especially worth noting the derivative **4b** did not show cytotoxicity (in the range of tested concentrations) against kidney cells.

**Figure 2:**
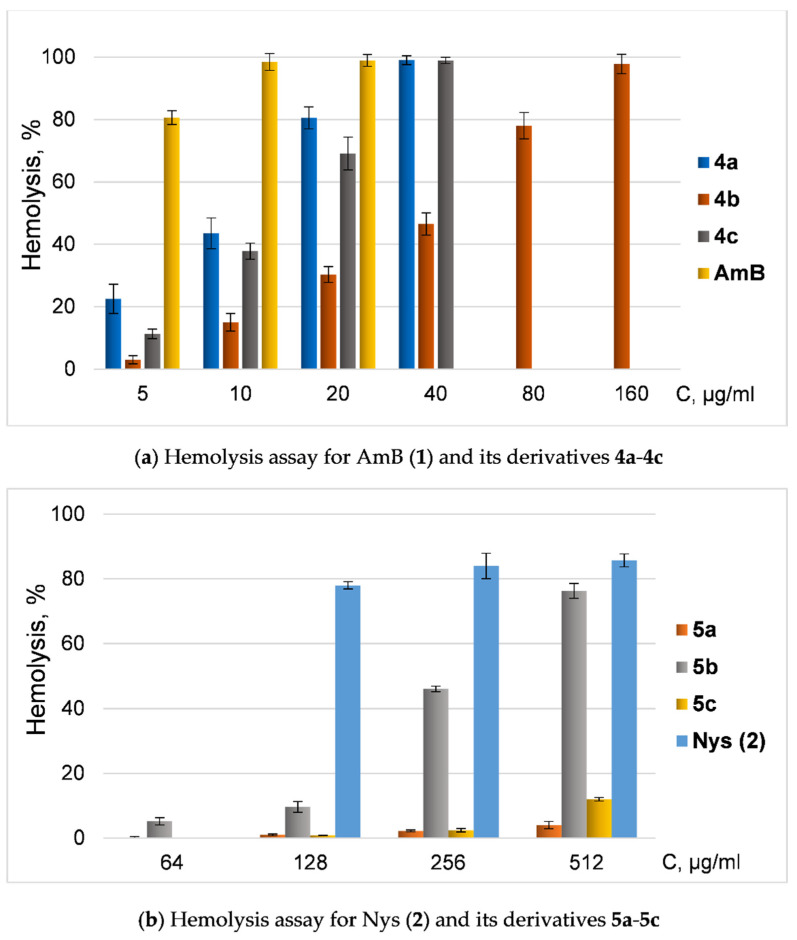
Hemolysis assay of quaternized polyenes. (**a**) Dependence of hemolysis ratio (%) from concentration of AmB (**1**) and its derivatives **4a**-**4c**; (**b**) dependence of hemolysis ratio (%) from concentration (μg/mL) of Nys (**2**) and its derivatives **5a**-**5c**.

Since the toxicity of amphotericin B and nystatin is associated with their hemolytic activity, we also tested the ability of new derivatives of AmB and Nys to disrupt red blood cells (hemolysis) (Figure 2).

All semisynthetic derivatives of AmB and Nys were less toxic in hemolysis tests than native antimycotics. However, derivatives of amphotericin and nystatin with similar modifications had contrasting effects on blood cells: AmB amide **4b** was significantly less toxic than amphotericin and even other tested amides, while Nys amide **5b** had the most pronounced ability to induce hemolysis in a series of nystatin amides (but was still less toxic than Nys).

### 2.4. Antibiofilm Activity 

To test the antibiofilm activity, the leader compound **4b** was selected in terms of activity/toxicity ratio among all set of obtained polyenes. The selection of strains was based on a review of publications on the clinical significance of candidiasis pathogens. Among non-*albicans* strains, *C. tropicalis* and *C. parapsilosis* are of clinical importance. These strains, even more so than *C. albicans*, tend to form biofilms that are resistant to the effects of antimycotic drugs [31,32].

At the first stage, the minimum inhibition concentration (MIC) of plankton cultures to the tested microorganisms *C. albicans* 604M, *C. tropicalis* 56-05, and *C. parapsilosis* 58L was determined (Table 3). The activity of the semi-synthetic derivative **4b** practically did not differ from the activity of the parent amphotericin B for all test cultures. Next, the cell viability of *Candida* species in biofilms exposed to the tested polyenes was evaluated using the MTT assay (Table 3). The concentration of derivatives that resulted in a 50% decrease in the relative metabolic activity of the cells in biofilm was considered the sessile minimum inhibitory concentration—SMIC_50_—for the biofilm phase of growth. Similarly, a value resulting in a decrease in a 80% was considered the SMIC_80_ [33,34].

The ratio of dead, damaged, and living cells in biofilms was also evaluated using fluorescent staining of DAPI (4′,6-diamidino-2-phenylindol)—PI (propidium iodide). Unlike in the case of mammalian cells, DAPI does not penetrate *Candida* cells with an intact cell membrane, and staining *Candida* cells with DAPI means permeabilization of their cell membrane has occurred [35]. Thus, the nature of DAPI-PI staining makes it possible to distinguish between living (DAPI/PI-negative), damaged (DAPI- positive), and dead cells (PI-positive).

**Figure 3:**
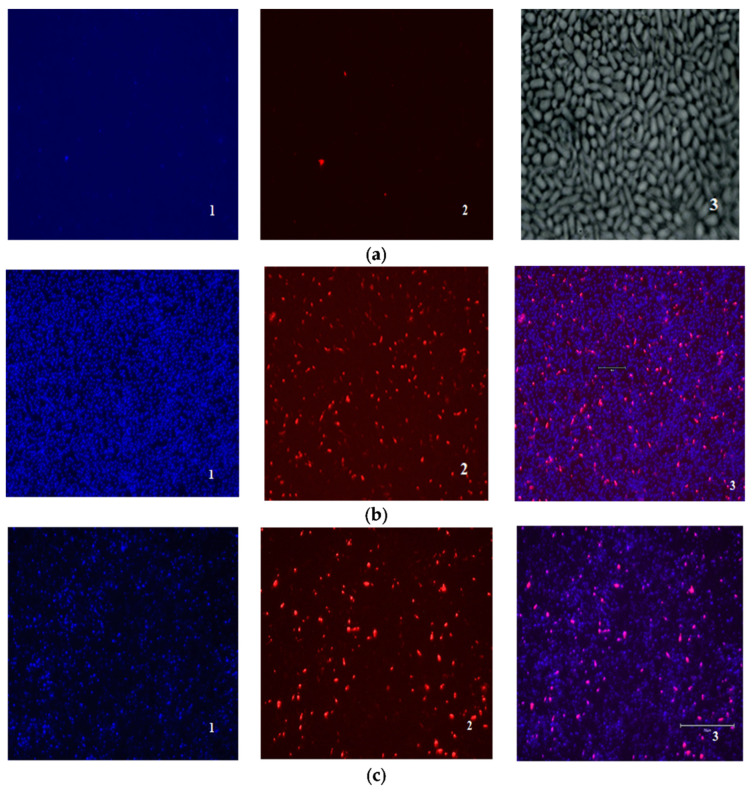
Evaluation of the ratio of dead and living cells in biofilms by fluorescence microscopy: (**a**) control biofilm *Candida parapsilosis* 58L (1—DAPI, 2—PI, 3—×400 light microscopy); (**b**) biofilm *Candida parapsilosis* 58L after 24 h of incubation in the presence of AmB (**1**) at a concentration of 1 × MIC (hereinafter 1—DAPI, 2—PI, 3—superimposed images of DAPI/PI); (**c**) *Candida parapsilosis* 58L biofilm after 24 h of incubation in the presence of a **4b** derivative at a concentration of 2 × MIC.

No fluorescence was detected when the cells were exposed to compound **4b** at a concentration of 1 × MIC. Amphotericin B (1 × MIC) and derivative **4b** in a higher concentration (2 × MIC) showed a similar effect (Figure 3).

Metabolic activity measurements and coloring with fluorescent dyes allowed us to assess the viability of cells in a biofilm but did not provide an indication of its structure. The analysis by scanning ion conductance microscopy (SICM) allowed for evaluating the change in the density of living biofilms without additional fixation and without destroying its structure [36].

**Figure 4:**
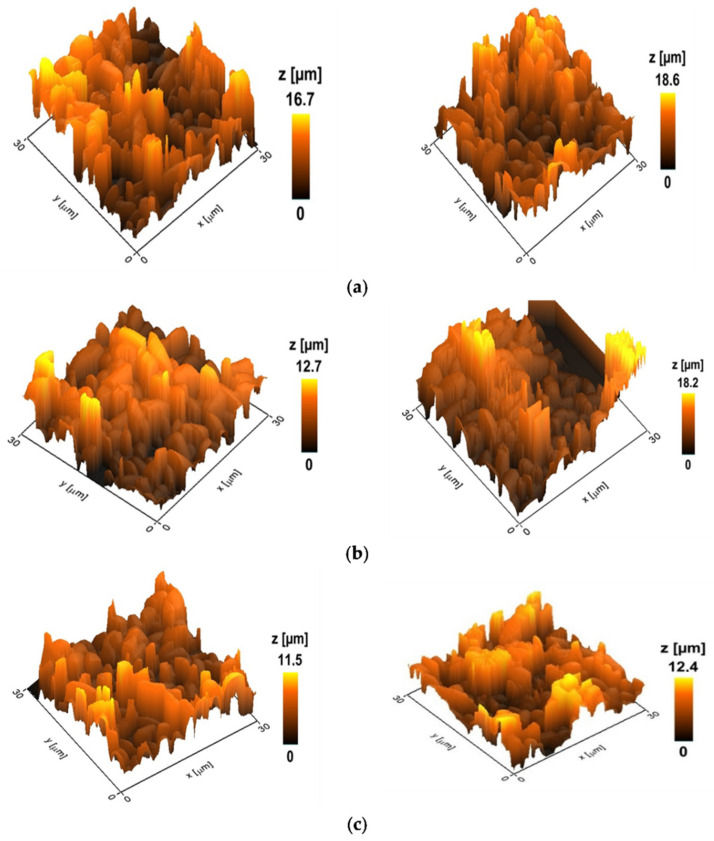
Topography of *Candida parapsilosis* 58L biofilms; the z-scale indicates the heights of cellular biofilms. (**a**) Control (untreated) biofilms. The average height is 17.2 microns (hereinafter *p* < 0.05). (**b**) Biofilm after 24 h incubation with AmB at a concentration of 2 μg/mL. The average height is 15.2 microns. (**c**) Biofilm after 24 h incubation with a derivative of **4b** at a concentration of 2 μg/mL. The average height is 11.8 microns.

Figure 4 showed the topography of the selected variants with the maximum and minimum heights of the control biofilms and biofilms treated with amphotericin B and derivative **4b**. The height values of the biofilms are calculated as the average of the obtained 5–10 images in the group.

The data obtained indicate a more pronounced effect of the semi-synthetic derivative **4b** on the structure of biofilms compared to the paternal antifungal. So, after 24 h of incubation with AmB at a concentration of 2 μg/mL, the height of the biofilm differed from the control by 12%. In contrast, for the derivative **4b**, this indicator was 31%. An analysis of the mechanical data of the surface of *C. parapsilosis* biofilms showed that incubation with amphotericin B and its derivative led to a loss of rigidity and a decrease in the adhesive ability of cells, rendering the biofilms more elastic. 

Evaluating the activity of amphotericin B during the prolonged incubation of biofilms is challenging due to its lability at elevated temperatures, which results in a decrease in inhibitory activity against *C. albicans* [37]. For a comparative analysis of the effect of amphotericin B and derivative **4b** on the 48 h biofilm, the biofilm was re-treated with experimental samples after 24 h. The adhered *C. parapsilosis* cells were washed and a fresh nutrient medium with the studied compounds was introduced at the same concentration (2 μg/mL) and incubated for another 24 h.

The size of the control 48 h biofilms increased, with average height values reaching 45 ± 1.5 microns. SICM analysis indicated that derivative **4b** reduces the biofilm volume and disrupts the cells’ adhesive abilities (in terms of the indentation) more effectively than AmB (Table 4).

**Figure 5:**
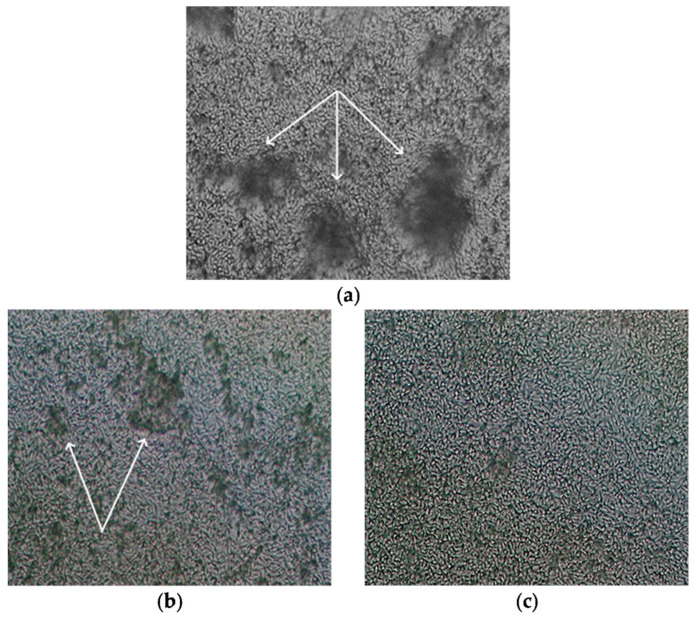
Light microscopy (magnification ×400) of 48 h dense biofilms of *C. parapsilosis* 58L (agglomerates growing from cells adhered to the bottom of the cup are indicated by white arrows): (**a**) without treatment; (**b**) after exposure to amphotericin B; (**c**) exposure to derivative **4b**.

Qualitative characteristics of 48 h dense biofilms of *Candida parapsilosis* 58L after exposure to amphotericin B and derivative **4b** are shown in Figure 5 (light microscopy, magnification ×400). Before the study, the biofilm was washed with saline solution, similar to the preparation for SICM analysis. When exposed to amphotericin B, the biofilm retains its structure, and agglomerates growing from cells adhered to the bottom of the cup are viewed, similar to the control; however, there are areas with reduced density. In the images of biofilms after exposure to derivatives **4b**, the destruction of biofilms is observed, the density is reduced, and there are no agglomerates.

### 2.5. Calcein Assay

Fluorescent marker release assay demonstrates the ability of polyene antifungals (parent polyene macrolides **1–3** and their derivatives) at 5 µM to disengage calcein from large unilamellar vesicles prepared from 67 mol% POPC and 33 mol% Chol or 33 mol% Erg. The POPC/Chol and POPC/Erg lipid mixture was used to mimic the polyene’s action on the mammalian and fungal cell membranes, respectively. The two-exponential dependences were used to fit the time dependences of calcein release induced by native polyenes and their derivatives from POPC/Chol- and POPC/Erg-liposomes as a first-order approximation. The characteristic parameters of dependences, maximal leakage, *RF_max_*, and the times related to fast and slow components, *t*_1_ and *t*_2_, respectively, are presented in Table 5. All AmB derivatives (**4a**-**4c**) showed more pronounced ability to induce calcein leakage from both POPC/Chol and POPC/Erg vesicles, resulted in a similar (**4a**, **4c**) or decreased (**4b**) ratio *RF_Erg_/RF_Chol_* compared to AmB itself. In contrast, Nys derivatives **5a**-**5c** demonstrated a lower ability to induce calcein leakage from both types of vesicles, but the resulting ratios *RF_Erg_/RF_Chol_* for these derivatives remained consistent with that of Nys. Derivatives **6a**-**6c** showed no significant difference from Nata in their ability to induce calcein leakage from POPC/Chol and POPC/Erg (except **6b**) vesicles (*RF_max_*). Notably, derivative **6b** was found to increase the release of the fluorescent marker from POPC/Erg liposomes by fivefold.

### 2.6. The Confocal Fluorescence Microscopy of Giant Unilamellar Vesicles

**Figure 6:**
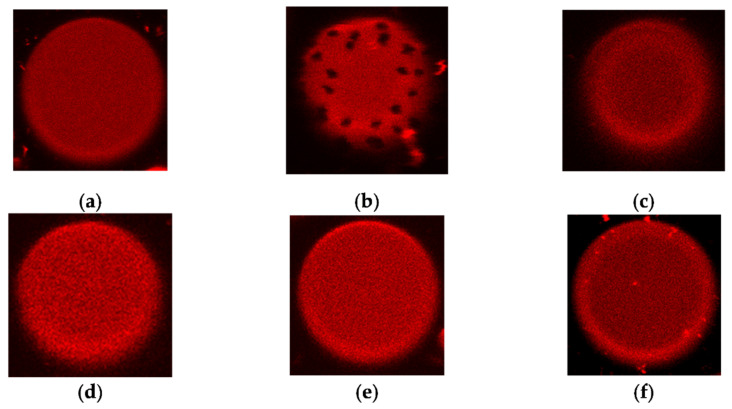
Typical fluorescence micrographs of POPC-liposomes demonstrating the homogeneity or inhomogeneity of membrane distribution of fluorescent-labeled lipid Rh-DPPE: (**a**) in the absence of polyenes (negative control); (**b**) in the presence of 150 μM AmB (**1**) (positive control); (**c**) in the presence of 150 μM Nata (**3**); (**d**) in the presence of 150 μM derivative **6a**; (**e**) derivative **6b**; (**f**) in the presence of 150 μM derivative **6c**. The size of each image is 15 μm × 15 μm.

Liposome composed of pure POPC does not produce visible phase segregation at room temperature (the melting temperature of POPC is about –2 °C [38]); fluorescent lipid Rh-DPPE, which is sensitive to the aggregate state of the lipid [39], is homogeneously distributed over the membrane (Figure 6a). An addition of AmB (**1**) led to the appearance of uncolored domains (Figure 6b). Similar effects were also produced by Nys (**2**), filipin, and AmB derivatives [40]. Taking into account the sensitivity of Rh-DPPE to the lipid packing and ordering, these effects were previously interpreted as the induction of phase segregation by the named polyene antifungals. Here, we tested whether Nata (**3**) and its derivatives **6a**-**6c** produce similar action. Figure 6 c-f illustrates that neither Nata (**3**) nor its derivatives **6a**-**6c** induce phase segregation in POPC vesicles at 150 μM. Increasing the concentration of Nata and its derivatives up to 300 μM led to the destruction of POPC vesicles. Similar effects were observed with liposomes composed of POPC/Erg (67/33 mol%).

**Figure 7:**
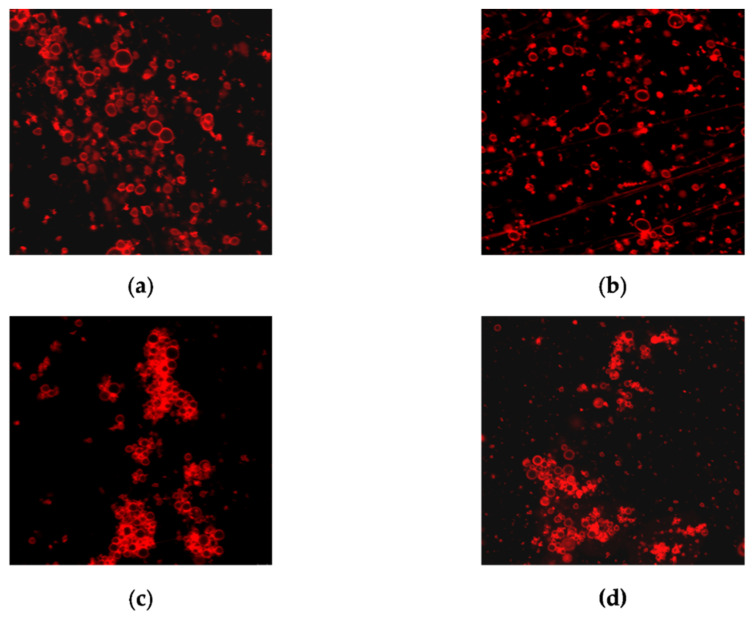
The effect on the aggregation of liposomes composed of POPC/Erg (67/33 mol %) of the 150 μM of (**a**) Nata; (**b**) derivative **6a**; (**c**) derivative **6b**; (**d**) derivative **6c**. The size of each image is 50 μm × 50 μm.

However, we noticed that compound **6b** at 150 μM produced a pronounced aggregation of the POPC/Erg (67/33 mol%) vesicles in contrast to Nata and its other derivatives (Figure 7). The aggregation of liposomes might explain the greatest ability of **6b** to induce calcein leakage from POPC/Erg-liposomes (Table 5) at membrane deformation.

### 2.7. Differential Scanning Microcalorimetry

The effect of AmB and Nys derivatives on the lipid packing was assessed using differential scanning microcalorimetry assay. The melting temperature (T_m_) and the half-width of the transition peak (T_1/2_) of DPPC/Erg (90/10 mol.%) in the absence of polyenes were equal to 40.3 ± 0.1 °C and 1.3 ± 0.2 °C, respectively. The parameters characterizing the phase behavior of DPPC/Erg (90/10 mol.%) in the presence of AmB, Nys, and their derivatives at different lipid/polyene molar ratios (50:1 and 10:1) are presented in Table 6. 

AmB and Nys and their derivatives did not practically affect the *T_m_* and *T*_1/2_ values of DPPC/Erg (90/10 mol.%) at a molar lipid/polyene ratio of 50:1 (the changes did not exceed 0.3 °C). Increasing the polyene concentration of AmB, **4a**, **4b**, Nys, **5a**, and **5b** up to a molar ratio of 10:1 produced slight effects (the changes did not exceed 0.5 °C). Raising the molar content of **4c** and **5c** up to a 10:1 ratio led to significant increase in *T*_1/2_ by 0.7–2.6 °C (Table 6). An increase in T_m_ in the presence of all tested polyenes at a 10:1 molar ratio might be explained by the formation of a more ordered lipid phase enriched with antimycotics. The data obtained were in agreement to the results of confocal fluorescence microcopy demonstrating the inhomogeneity of Rh-DPPE distribution in the presence of AmB. The ability to increase lipid packing was higher in the case of the AmB and Nys derivatives **4c** and **5c** with a trimethylammonium radical compared to other quaternized amides. 

### 2.8. Ion Channel Recording

**Figure 8:**
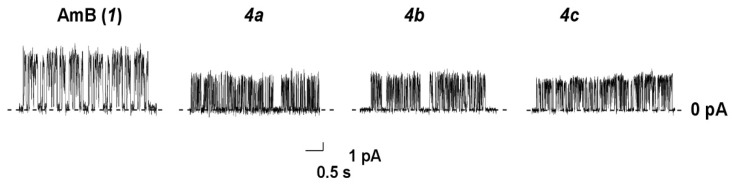
Current fluctuations corresponding to openings and closures of single channels induced by AmB, **4a**, **4b**, and **4c** in the lipid bilayers composed of DPhPC/Erg (67/33 mol.%) and bathed in 2.0 M KCl (pH 7.4). The transmembrane voltage was equal to 200 mV.

Figure 8 presents the current fluctuations corresponding to openings and closures of single channels formed by AmB (**1**) and its tested amides **4a**, **4b**, and **4c** in the lipid bilayers composed of DPhPC and Erg (67/33 mol.%) and bathed in 2 M KCl (pH 7.4) at transmembrane voltage 200 mV.

One can see that all tested derivatives (**4a**-**4c**) produced pores of smaller amplitude than AmB did under the same conditions. The conductance of AmB and its derivatives at 200 mV, the mean dwell times, *τ*, and the probability of polyene channels to be open, *P_op_*, are presented in Table 7.

**Figure 9:**
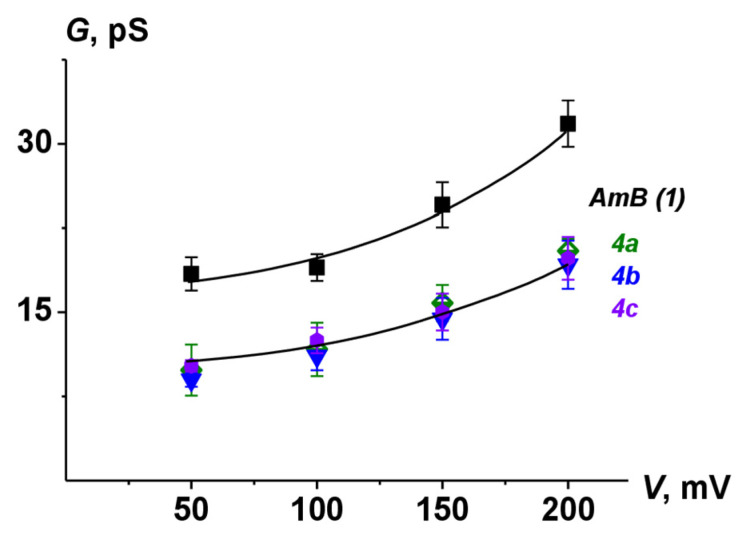
*G*–*V* curves of single channels produced by AmB, **4a**, **4b**, and **4c**. Membranes were made from DPhPC/Erg (67/33 mol.%) and bathed in 2.0 M KCl (pH 7.4).

The lifetime and *P_op_* of pores induced by **4a**-**4c** were lower than the corresponding parameters for AmB channels. These data were in a good agreement with previously published results [24,26,41]. Figure 9 shows *G*(*V*) characteristics of pores produced by AmB and its derivatives. The chemical modification of natural polyene molecules led to a decrease in channel amplitude without affecting the shape of nonlinear *G*(*V*)-dependence compared to the parent antifungal. 

At neutral pH, single Nys channels are characterized by conductance less than the level of current noise (of about 0.5 pA) [42]; therefore, the registration of the single step-like transmembrane current fluctuations that might be related to single channels produced by Nys or their derivatives could not be performed at neutral pH. Since Nata does not form pores in the Erg-containing membranes [11], we did not test the pore-forming ability of its derivatives.

**Figure 10:**
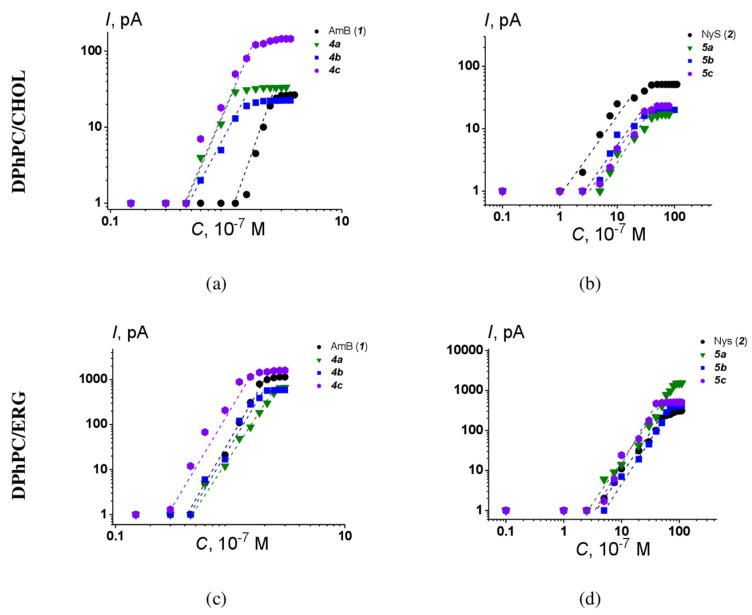
Bilogarithmic dependence of the steady-state polyene-induced transmembrane current on the concentration of (**a**) AmB derivatives **4a**-**4c** in the bilayer bathing solution, membranes composed of DPhPC/Chol (67/33 mol.%); (**b**) Nys derivatives **5a**-**5c** in the bilayer bathing solution, membranes composed of DPhPC/Chol (67/33 mol.%); (**c**) AmB derivatives **4a**-**4c** in the bilayer bathing solution, membranes composed of DPhPC/Erg (67/33 mol.%); (**d**) Nys derivatives **5a**-**5c** in the bilayer bathing solution, membranes composed of DPhPC/Erg (67/33 mol.%). The transmembrane voltage was equal to 100 mV. Membranes bathed in 2.0 M KCl (pH 7.4). The relation between the color symbol and the compound is provided on the figure.

The one-side addition of AmB or Nys to the membrane bathing solution produced an increase in macroscopic conductance in a dose-dependent manner. Figure 10 shows the examples of logarithmic plots of the dependence of steady-state transmembrane current flowing through membranes composed of DPhPC and CHOL or ERG at 100 mV on the concentration of the tested AmB and Nys derivatives.

The slopes of the linear regression of the growth section of presented curves for AmB and Nys derivatives are close to 5 ÷ 6 and 2 ÷ 3, respectively. This means that the steady-state polyene-induced conductance is proportional to about the 4–5th and 2–3rd power of the AmB and Nys derivative concentration, respectively. The results obtained indicate that the number of polyene–sterol complexes forming the channel does not depend on the type of chemical modification of the parent molecules. Similar results were obtained for other AmB and NyS derivatives [24].

## 3. Discussion

The initial attempts to modify the structure of polyene antimycotics were made back in the 1970s [25,43], and decades later, this area of research continues to attract global interest [7,8,9]. Numerous papers have been published focusing on both the mode of action of polyenes and the relationship between their structure and biological activity [10,14,15,44,45]; however, it remains challenging to consolidate all experimental data into a coherent predictive model. It is well-established that amphotericin B can form pores in cell membranes, with sterols (ergosterol and cholesterol) playing a critical role in the manifestation of the biological action of antifungal polyenes. A consistent pattern has been observed in the chemical transformation of polyene structures: modifying the carboxyl group into non-ionized group or the introduction of the side chain with an additional basic group typically results in reduced toxicity while preserving or enhancing antifungal activity [8,22,23,24,25,26]. Independent studies also highlight the key role of the positive charge of the mycosamine amino group in the antifungal activity of amphotericin [18,25].

Quaternization is a simple and promising reaction for modifying biologically active compounds; since such derivatives can form salts with improved water solubility at various pH levels, they can exhibit changes in electrostatic properties and in their ability to penetrate cell membranes. To our knowledge, no one has previously studied the impact of a constant positive charge on biological activity and interaction with model membranes. In this study, we chose polyene amides with two types of quaternized groups—those bearing an pyridinium ring and those with a short alkyl chain with quaternary trimethylammonium. Our choice was based on previously obtained data suggesting that introducing an ethylenediamine residue improves the biological properties of polyene antifungals [22,24] and that amphotericin B picolylamides exhibit high antifungal activity [26].

Our research indicates that the nature of the introduced quaternized residue is more significant than the mere presence of a constant positive charge. We also can conclude that quaternization of DMAE-amides [22,24] and 3-picolylamides [26] of polyenes with a methyl group led to slightly decreasing their antifungal activity. However, all quaternized nystatin and natamycin derivatives were found to be less cytotoxic to human embryonic kidney cells (HEK293) and human skin fibroblast cells hFB-hTERT6. It should be noted that a similar trend was observed for nystatin and natamycin amides, where the presence of a positive charge in the amide residue depends on pH [22,24]. Regarding the quaternized amphotericin B derivatives, only one derivative (**4b**) exhibited less cytotoxicity compared to the others. All quaternized amides of AmB and Nys also demonstrated reduced hemolytic activity compared to the paternal antifungals.

Our data suggest that among the tested polyenes with quaternized residues, derivatives containing in their structure a fragment of *N*-methyl-4-(aminomethyl)pyridine-1-ium **4b**, **5b**, and **6b** are particularly interesting due to their biological activity. They possessed a high antifungal activity, either equal to or slightly superior to natural antimycotics, and also demonstrated reduced cytotoxicity. Notably, derivative **4b** did not show cytotoxicity (within the range of tested concentrations) against kidney cells and was significantly less toxic for red blood cells than amphotericin B. The antibiofilm activity of this derivative was comparable to amphotericin B, but when considering the degree of eradication of biofilms treated with polyenes, the effect of amide **4b** significantly surpassed that of the paternal antifungal. Nys amide **5b** was also less toxic than nystatin but, in contrast with AmB amide **4b**, showed the most pronounced hemolytic activity within its series. Natamycin amide **6b** at 150 μM caused pronounced aggregation of the POPC/Erg (67/33 mol%) vesicles in contrast to other tested natamycines. This might explain why **6b** can induce calcein leakage from POPC/Erg-liposomes, unlike the native antimycotic and other tested quaternized Nata derivatives. A similar effect (the ability to induce calcein leakage from POPC/Erg-liposomes) was also observed for natamycin amides with long lipophilic chains in the amide moiety [22]. 

However, a study on liposomes that mimic the composition of fungal and mammalian cell membranes has shown that amides bearing a quaternized moiety did not significantly differ from their natural counterparts. The observed decrease in cytotoxicity for the quaternized derivatives of antifungal polyenes was not accompanied by an increase in selectivity towards lipid membranes containing ergosterol over those with cholesterol (except Nata derivative **6b**). The threshold concentrations of Nys derivatives required to observe an increase in ionic permeability of Chol-containing bilayers were higher than that of the parent antifungal (Table 5). This fact may be attributed to the lower toxicity of quaternized derivatives compared to nystatin. As was established earlier [24,26], modifying the carboxyl group into an amide led to a decrease in both the lifetime and the probability of transmembrane pores being open. We can conclude that an introduction of side chains with quaternized radicals did not significantly affect the characteristics of the pores formed by AmB amides.

This suggests that metabolic pathways triggered by the action of quaternized polyenes inside the membrane or inside the cell may play an important role in antifungal action selectivity. Therefore, this area warrants further research.

## 4. Materials and Methods

### 4.1. General

All chemicals were purchased from commercial suppliers and used as received. Amphotericin B (AmB), Nystatin (Nys), Natamycin (Nata), benzotriazol-1-yl-oxytripyrrolidinophosphonium hexafluorophosphate (PyBOP), dimethylsulfoxide (DMSO), *N,N*-dimethylacetamide (DMAA), triethylamine calcein, KCl, HEPES, KOH, triton X-100, iodomethane, 3-, 4-picolylamines, and *N,N*-dimethylethylenediamine were purchased from Sigma-Aldrich^®^ (Merck KGaA, Darmstadt, Germany). Synthetic 1,2-diphytanoyl-*sn*-glycero-3-phosphocholine (DPhPC), 1-palmitoyl-2-oleoyl-*sn*-glycero-3-phosphocholine (POPC), 1,2-dipalmitoyl-*sn*-glycero-3-phosphocholine (DPPC), 1,2-dipalmitoyl-*sn*-glycero-3-phosphoethanolamine-N-(lissamine rhodamine B sulfonyl) (Rh-DPPE), ergosterol (Erg), and cholesterol (Chol) were obtained from Avanti^®^ Polar Lipids (Alabaster, AL, USA). Water was distilled twice and deionized. Solutions of 2.0 or 0.1 M KCl were buffered using 5 mM HEPES-KOH at pH 7.4. Thin-layer chromatography (TLC) analysis was performed on 0.20 mm silica gel 60 F_254_ plates (aluminum sheets 20 × 20 cm) (Macherey-nagel, Duren, Germany) in n-PrOH–EtOAc–NH_4_OH, 3:3:2. All final compounds were purified to over 90% using reverse-phase flash chromatography on C18 silica gel cartridges (Biotage^®^ SNAP, Stockholm, Sweden). The purity was determined via reverse-phase high-performance liquid chromatography (HPLC) carried on the Shimadzu LC 20AD series instrument (Kyoto, Japan) with a Kromasil-100 C18 column (4.6 × 250 mm, particle size 5 μm, Ekzo Nobel, Sweden). An injection volume was 20 μL with substance concentrations ranging from 0.01 to 0.02 mg/mL at a flow rate of 1.0 mL/min. Detection was carried out using a diode array ultraviolet detector at 408 nm for amphotericin B derivatives, at 320 nm for nystatin derivatives, and at 305 for natamycin derivatives. The system employed a buffer of 0.2% HCOONH_4_ at pH 4.5 and an organic phase of acetonitrile, with the proportion of acetonitrile varying from 30 to 70% for 30 min. NMR spectra (except for **4b**) were recorded on a Bruker Avance III 500 MHz NMR spectrometer (BrukerDaltonics GmbH, Bremen, Germany) with resonance frequencies of 500 MHz for ^1^H and 125 MHz for ^13^C. NMR spectra for **4b** were recorded on a Bruker Avance 600 spectrometer (BrukerDaltonics GmbH, Bremen, Germany) with resonance frequencies of 600 MHz for ^1^H and 150 MHz for ^13^C. Spectra were recorded in DMSO-d6 (except for **4b**) and in CD_3_OD (**4b**) solutions at 303 K and were referenced against residual solvent signals: DMSO-d6—2.50 ppm for ^1^H and 39.50 ppm for ^13^C, CD_3_OD—3.31 ppm for ^1^H and 49.2 ppm for ^13^C. Mixing times ranging from 10 ms to 180 ms were used to mimic stepwise magnetization propagation. For 2D TOCSY, a mixing time of 80 ms was used. For 2D ROESY and NOESY experiments, mixing times of 300 and 500 ms were used, respectively. ESI MS spectra were recorded on a Bruker microTOF-Q II™ instrument (BrukerDaltonics GmbH, Bremen, Germany).

### 4.2. Quaternized Derivatives of Amphotericin B, Nystatin, and Natamycin (4a-c, 5a-c, 6a-c) (General Method)

AmB or Nys or Nata (0.22 mmol) were dissolved in DMAA (5.0 mL) under argon flow, and then triethylamine (1.2 mmol) and the corresponding quaternized salt (1.1 mmol) were added. PyBOP (172 mg, 0.33 mmol) was added portionwise with stirring to the reaction mixture over 20 min. The reaction mixture was stirred for 1.5 h, and then Et_2_O (50 mL) was added. The reaction mixture was shook vigorously, and the Et_2_O layer was decanted; this procedure was repeated 4 times. The oil formed was dissolved in methanol, diluted with Et_2_O, and the precipitate was filtered off, washed with Et_2_O, and dried under vacuum. The progress of the reactions, chromatography purification, and the purity of final compounds were monitored by TLC and HPLC analysis. The crude amide was purified by reverse-phase flash chromatography on Biotage Sfar C18 (6 and 12 g) cartridges, respectively. Reverse-phase chromatography was performed as follows: the amide was dissolved in 0.01% aq. HCl (2 mL) and put on a column. The elution was carried out with (A) H_2_O, then 0.5% aq. AcOH and (B) acetonitrile (0 →20 (5 CV), 20→30 (10 CV), 30–100 (3 CV) B%). Fractions containing the target compound were combined and evaporated to a small volume. The addition of Et_2_O to the solution provided the precipitate of a dichloride of a targeted compound that was filtered off and dried.

*N*-((1-methylpyridin-1-ium-3-yl)methyl)amide of AmB (**4a**): yield: 90 mg (38%); orange powder; Rf 0.16; Rt 10.70 min; purity 95%; HRMS (ESI) m/z: [M + H]^+^ Calc. for C_54_H_82_N_3_O_16_^+^ 1028.5690, found 1028.5656.

*N*-((1-methylpyridin-1-ium-4-yl)methyl)amide of AmB (**4b**): yield: 61 mg (26%); orange powder; Rf 0.17; Rt 10.71 min; purity 94%; HRMS (ESI) m/z: [M + H]^+^ Calc. for C_54_H_82_N_3_O_16_^+^ 1028.5690, found 1028.5708.

*N*-(2-(trimethylammonio)ethyl)amide of AmB (**4c**): yield: 86 mg (37%); yellow powder; Rf 0.15; Rt 14.39 min; purity 92%; HRMS (ESI) m/z: [M + H]^+^ Calc. for C_52_H_86_N_3_O_16_^+^ 1008.6003, found 1008.5993.

*N*-((1-methylpyridin-1-ium-3-yl)methyl)amide of Nys (**5a**): yield: 60 mg (25%); beige powder; Rf 0.21; Rt 10.98 min; purity 96%; HRMS (ESI) m/z: [M + H]^+^ Calc. for C_54_H_84_N_3_O_16_^+^ 1030.5846, found 1030.5857.

*N*-((1-methylpyridin-1-ium-4-yl)methyl)amide of Nys (**5b**): yield: 41 mg (17%); yellow powder; Rf 0.20; Rt 11.07 min; purity 90%; HRMS (ESI) m/z: [M + H]^+^ Calc. for C_54_H_84_N_3_O_16_^+^ 1030.5846, found 1030.5839.

*N*-(2-(trimethylammonio)ethyl)amide of Nys (**5c**): yield: 105 mg (45%); beidge powder; Rf 0.17; Rt 14.85 min; purity 94%; HRMS (ESI) m/z: [M + H]^+^ Calc. for C_52_H_88_N_3_O_16_^+^ 1010.6159, found 1010.6175.

*N*-((1-methylpyridin-1-ium-3-yl)methyl)amide of Nata (**6a**): yield: 89 mg (35%); beige powder; Rf 0.28; Rt 6.44 min; purity 95%; HRMS (ESI) m/z: [M + H]^+^ Calc. for C_40_H_56_N_3_O_12_^+^ 770.3859, found 770.3891.

*N*-((1-methylpyridin-1-ium-4-yl)methyl)amide of Nata (**6b**): yield: 50 mg (20%); orange powder; Rf 0.24; Rt 6.05 min; purity 97%; HRMS (ESI) m/z: [M + H]^+^ Calc. for C_40_H_56_N_3_O_12_^+^ 770.3859, found 770.3920.

*N*-(2-(trimethylammonio)ethyl)amide of Nata (**6c**): yield: 143 mg (58%); beige powder; Rf 0.22; Rt 12.38 min; purity 99%; HRMS (ESI) m/z: [M + H]^+^ Calc. for C_38_H_60_N_3_O_12_^+^ 750.4172, found 750.4177.

### 4.3. Antifungal Activity Testing

Strains of *Candida* spp. (*C. parapsilosis* 58L, *C. albicans* 604M, *C. albicans* 8R, *C.glabrata* 61L, *C. tropicalis* 3010, *C. krusei* 432M) used in this study were obtained from the Medical Microbiology Laboratory of the State Research Center for Antibiotics (Moscow, Russia). Cells of *Candida* spp. and spores of filamentous fungi were stored in medium supplemented with 10% (vol/vol) glycerol at −80 °C.

The reference strain *C. parapsilopsis* ATCC 22019 and amphotericin B were used as controls in each experiment.

Antifungal susceptibility testing was performed by the EUCAST broth microdilution (E.DEF 7.3.2 and E.DEF 9.3.2) methods in the liquid nutrient medium PRMI 1640 with 2% (m/v) glucose, with L-glutamine and without bicarbonate (Merck KGaA, Darmstadt, Germany). MOPS (Sigma-Aldrich, St. Louis, MO, USA) was used as a buffer to this medium at a final concentration of 0.165 mol/L at pH 7.0. The studies were performed in sterile disposable 96-well plates with flat-bottomed wells with a capacity of 300 µL (Corning, NY, USA). To evaluate sensitivity to antimicrobial agents, 24 h old cultures of *Candida* spp. and a ~3-week-old culture of *A. fumigatus* grown in Sabouraud dextrose agar (Sigma-Aldrich, St. Louis, USA) were used. Substances of antifungals were dissolved with dimethyl sulfoxide (DMSO) (Merck KGaA, Darmstadt, Germany). Minimum Inhibitory Concentration (MIC) was defined as the lowest concentration that resulted in complete growth inhibition after incubation for 24, 48 h for *Candida* spp. and 48–72 h for *A. fumigatus.*

### 4.4. Cell Culture and Antiproliferative Activity

Cell line hFB-hTERT6 (non-malignant human skin fibroblasts) was obtained via the lentiviral transduction of full-length TERT genes under a cytomegalovirus promoter (generated at Engelhardt Institute of Molecular Biology, Moscow by Dr. E. Dashinimaev; gift of Prof. A. Shtil). Cell line HEK293 (human embryonic kidney cells) was obtained from American Type Culture Collection, USA. HEK293 and hFB-hTERT6 cells were cultured in Dulbecco’s modified Eagle’s medium (PanEco, Moscow, Russia) supplemented with 10% fetal calf serum (HyClone, Logan, UT, USA), 2 mM L-glutamine, 100 U/mL penicillin, and 100 mg/mL streptomycin at 37 °C, 5% CO_2_ in a humidified atmosphere. In the experiments, cells were used in the logarithmic phase of growth. Tested compounds were dissolved in DMSO as 100 mM or 10 mM stock solutions followed by serial dilutions with medium immediately before experiments. The cytotoxicity of tested compounds was determined in a formazan conversion assay (MTT test) by the standard method [24].

### 4.5. Hemolysis Assay

The analysis was carried out according to the technique described in the work of Biernasiuk A. et al. [46,47]. The studied samples were dissolved in DMSO and further prepared as stated above. Fresh donor blood, collected no later than 24 h prior, was centrifuged at 1700 rpm for 5 min. The supernatant was removed, and the pellet was resuspended in phosphate-buffered saline (PBS) pH 7.4 up to the initial volume. This washing process was repeated three times under the same conditions until a clear, colorless supernatant was obtained. A 2% suspension of the washed blood cells was then prepared in PBS. In a microplate, 50 µL of the red blood cell suspension and 50 µL of the test compound solution were added to each well. A 4% Triton X-100 solution was used as a positive control, while PBS served as the negative control. The plates with samples were incubated at 37 °C for 60 min. Optical density was measured at 405 nm, and the percentage of hemolysis was calculated relative to the positive and negative controls [47].

### 4.6. Antibiofilm Activity

Biofilms were formed in 96-well plates for immunological studies in RPMI-MOPS medium (SIGMA, St. Louis, USA). The inoculum was diluted to a titer of 10^6^ CFU/mL and an amount of 100 μL was transferred into the wells of the plate and kept for 2 h at 36 °C. After that time, unattached cells were removed by washing twice, adding 200 μL of sterile saline solution to each well. After removing the washing solution, fresh medium was added and incubated in accordance with the objectives of the study for 24–48 h at 36 °C. After 24 h of incubation, plankton was removed, washed twice with 200 μL of saline, and refilled with 100 μL of fresh RPMI-MOPS medium.

The studied drugs were added to the biofilms in a range of concentrations from 64 to 0.5 μg/mL and incubated for 24 h at 36 °C. The effect of the drugs was assessed by the MTT test. To do this, after removing the liquid phase, 50 μL of MTT solution was added to each well of the plate and incubated for 2 h at 36 °C. At the end of the incubation time, the liquid was removed and 100 μL of DMSO was added to each well. The mixture was kept in the dark at room temperature for 2 h until the formazan was completely dissolved. Optical density (OD) was measured at 580 nm on a Bioscreen plate reader with an automated system (Labsystems, Vantaa, Finland) using software. The OD values of the control biofilms without exposure were taken as 100%. To approximate the dose–effect in Microsoft Excel, logistic regression models were used; with 2–3 degrees of polynomial, the reliability of R^2^ was 0.96–0.98. Based on graphical data, concentrations were determined for sessile minimal inhibitory concentration (SMIC) 50% and 80% levels. To assess the statistical reliability of the results, each experimental variant was analyzed in 4–6 repetitions and in 2–3 independent experiments.

### 4.7. Studying the Effect of Drugs on Biofilms by Scanning Ion Conductance Microscopy (SICM)

Biofilms were prepared in Petri dishes with a diameter of 50 and 35 mm (Medpolymer, St. Petersburg, Russia) without additional processing. Biofilm preparation was carried out as previously described. After 48 h of incubation, fresh broth containing the test samples (at a concentration of 2 µg/mL) was introduced into the washed cups and incubated for another 24 h at 36 °C. Immediately before analysis, the broth was removed and the cups were rinsed again before being filled with saline solution. The examination of biofilms—both the control and those incubated with the studied compounds for 24–48 h—was conducted without any special treatment or fixation. Scanning was performed using the scanning ion conductance microscope ICAPPIC (ICAPPIC Limited, London, United Kingdom). The SICM by ICAPPIC was mounted on an inverted optical microscope Eclipse Ti-2 (Nikon, Tokyo, Japan) and covered with a Faraday cage for electrical noise shielding. The ICAPPIC Universal Controller and Piezo Control System (ICAPPIC Limited, London, United Kingdom) was used for piezo positioning and feedback control. A MultiClamp 700B amplifier (Molecular Devices, San Jose, CA, USA) was used for ion current monitoring. Nanopipettes created with a P-2000 laser puller (Sutter Instruments, Novato, CA, USA), featuring a radius of 40–60 nm, were utilized. The samples were positioned on a scanning platform, and a reference electrode was immersed into the liquid. A nanopipette filled with saline solution was secured in the piezoactuator, and then a working electrode was inserted into it. The electrical noise was verified as not exceeding 1 pA in the MultiClamp program window. Scanning was executed at a constant potential of 200 with high-frequency signal filtering (Bessel) at 4 kHz in hopping mode, observing an ion current drop of 0.3–0.6%, dependent on the ion current passing through the nanopipette. The loading rate was set at 100 microns/s. Surface topography was recorded upon a 0.5% drop from the initial ion current value. The stop point Sp1 indicates the topography measurement point, where the probe halts at a distance from the object equal to the capillary’s radius upon an ion current drop. Mechanical properties of the biofilm were assessed based on stopping points Sp2 and Sp3, which denote the capillary’s contact with the object at positions along zsp2 and zsp3, where the ion current drops by 1% (indicating an undeformable region) and 2% (indicating elastic deformation of the sample), respectively. The indentation value (δ) was determined by the height difference between the first second and third settings:

Δ = zsp2 − zsp3,(1)

where zsp2 is the height of the probe at position sp2 and zsp3 is the height of the probe at position sp3.

The scanning area was 40 × 40 mm^2^ with a resolution of 312 nm for imaging biofilms and 1.5 × 1.5 mm^2^ with a resolution of 60 nm for imaging single cells. To avoid the impact of transverse forces from the probe on the cell due to the weak fixation of the sample on the substrate, scanning was conducted in pre-scan hop mode with a 7-micron amplitude. This approach allows for a preliminary assessment of the surface roughness across 25 quadrants, which collectively constitute the entire scanning area. For each group of biofilms, 5–10 images were obtained. The average height of the biofilms was determined by measuring the height difference between the top of the biofilm and the point of the probe’s contact with the substrate surface. Deformation maps were created based on the indentation of the object by the probe, and the average indentation value for the centers of each cell within the biofilm was calculated.

### 4.8. Fluorescence Microscopy

The 24 h biofilms of *C. parapsilosis* 58 L, formed in 96-plate wells, were treated with AmB and derivative **4b** at concentrations equivalent to 1–2 MIC. The biofilms were incubated with the samples for 24 h under standard growth conditions. Following incubation with the studied samples and a control without treatment, the biofilms were washed twice with saline solution. Subsequently, 50 µL of DAPI (4′,6-diamidino-2-phenylindole, 1 µg/mL) was added and the biofilms were incubated at room temperature in the dark for 15 min. Then, 10 µL of PI (propidium iodide, 120 µM) was added, and the biofilms were further incubated at room temperature for 5 min. After exposure to the fluorescent dyes, the biofilms were washed with sterile saline solution. Fluorescence was analyzed using the EVOS m5000 imaging system with a 40× objective lens and appropriate filters for DAPI. In the case of PI, RFP and Cy5 filters were used with appropriate excitation/emission wavelengths of 355/433, 530/593 and 628/685 nm. The exposure time was set to 30 ms.

### 4.9. Statistical Analysis (Investigations of Biofilms)

The numerical values of the experimental data were organized in Microsoft Excel tables and imported into either IBM SPSS Statistics v23 or OriginPro 2020 (9.7) software. The average value and standard deviation (SD) for each group were calculated. To assess the differences between groups, nonparametric analysis using the Mann–Whitney test for independent variables was employed. For SICM data, the obtained values were analyzed using one-way analysis of variance (ANOVA). A two-sided Student’s t-test was applied, with a *p*-value of <0.005 considered statistically significant.

### 4.10. Calcein Release from Large Unilamellar Vesicles

To observe the membrane permeabilization induced by different polyenes, the fluorescence of calcein released from large unilamellar vesicles was utilized. The method for preparing liposomes composed of POPC/Chol (67/33 mol%) and POPC/Erg (67/33 mol%) was used as described in previous work [41]. Stock solutions (10 mM in DMSO or H_2_O) of the polyenes (AmB (**1**), Nys (**2**), Nata (**3**) and their derivatives) were used to add the tested compounds to calcein-loaded liposomes. Time-dependent increase in fluorescence of released calcein induced by 5 μM of polyenes was measured at 25 °C using a “Fluorat-02-Panorama” spectrofluorimeter (Lumex, St. Petersburg, Russia). Detection was performed over a period of 30–60 min, the excitation wavelength was 490 nm, and the emission wavelength was 520 nm 

At the conclusion of each experiment, triton X-100 (as detergent at a final concentration of 1%) was added, ensuring the complete disruption of liposomes and, consequently, the maximal release of calcein, resulting in peak fluorescence. 

The permeabilization potency of the tested polyenes was characterized by the relative intensity of calcein fluorescence (*RF*, %). *RF* was calculated using the following formula (2):

(2) $$RF\;=\;\frac{I-I_{0}}{I_{\max}/0.9-I_{0}}\cdot 100\%,$$

where *I* and *I*_0_ represent the calcein fluorescence intensities in the sample treated with AmB, Nys, and Nata, their derivatives, and in the untreated sample, respectively; *I_max_* denotes the maximum fluorescence intensity observed after liposome lysis induced by triton X-100. A factor of 0.9 was applied to account for the dilution of the sample by triton X-100. 

The time series data of calcein release were analyzed using a two-exponential function with characteristic times *t*_1_ and *t*_2_ corresponding to the fast and slow components of calcein release, respectively.

The values of *RF*, *t*_1_, and *t*_2_ were presented as *mean* ± *s.e.* (*p* ≤ 0.05).

### 4.11. Registration of Ion Channels in Planar Lipid Bilayers

Virtually solvent-free planar lipid bilayers were prepared according to the monolayer-opposition technique [48] on a 50 µm diameter aperture in a 10 µm thick Teflon film separating two (*cis* and *trans*) compartments of the Teflon chamber. The aperture was pretreated with hexadecane. The lipid bilayers were composed of DPhPC/Erg (67/33 mol%) or DPhPC/Chol (67/33 mol%). Once the membrane was completely formed and stabilized, the tested polyenes were added to the *cis*-compartments in a range of concentrations (diluted from a 1 mM stock solutions in DMSO or H_2_O) presented in Figure 5. Transmission voltage supply and measurement of the transmembrane current were performed using Ag/AgCl electrodes with agarose/2 M KCl bridges. “Positive voltage” refers to the condition in which the *cis*-side compartment was positive relative to the *trans*-side. All experiments were conducted at room temperature. The final solvent concentration in the chamber did not exceed 10^−4^ mg/mL and did not affect the stability or conductance of the bilayers.

Current measurements were performed using an Axopatch 200B amplifier (Molecular Devices, San Jose, CA, USA) in voltage clamp mode. Data were digitized by a Digidata 1440A and analyzed using a pClamp 10 (Molecular Devices, San Jose, CA, USA) and Origin 7.0 (OriginLab Corporation, Northampton, MA, USA). Data acquisition was conducted at a sampling frequency of 5 kHz with low-pass filtering at 1 kHz, and the current tracks were processed through an 8-pole Bessel 100-kHz filter.

Single-channel conductance (*G*) was defined as the ratio of the current flowing through a single polyene channel (*i*) to the transmembrane potential (*V*). The total number of events used for channel conductance fluctuation and dwell time (*τ*) analysis were 500 ÷ 1000 and 1500 ÷ 2000, respectively. The probability of the polyene channel being in an open state (*P_op_*) was calculated using the following formula (3):

(3) $$P_{op}=\frac{\tau }{\tau +\tau_{close}},$$

where *τ* is the dwell time of the single polyene channel and *τ_close_* is the time that the channel is in a closed state.

The values of the threshold polyene concentrations (*C_Chol/Erg_*) were determined by the intersections of the straight lines fitting the initial and growth portions of the bilogarithmic plots, which depict the dependence of the steady-state transmembrane current flowing through polyene-modified membranes at 100 mV on the concentration of the tested derivatives.

The values of *G* were presented as *mean* ± *s.d.* (*p* ≤ 0.05); *τ*, *P_op_*, and ***C_CHOL/ERG_*** were presented as *mean* ± *s.e.* (*p* ≤ 0.05).

### 4.12. Differential Scanning Microcalorimetry 

Differential scanning microcalorimetry experiments were conducted using a μDSC 7EVO microcalorimeter (Setaram, Caluire-et-Cuire, France). Giant unilamellar vesicles composed of DPPC/Erg (90/10 mol%) were prepared by the electroformation method with Vesicle Prep Pro (Nanion Technologies, Munich, Germany) following the standard protocol (3 V, 10 Hz, 1 h, 55 °C). The resulting liposome suspension contained 2 mM lipid was buffered by 5 mM HEPES at pH 7.4. AmB, Nys, and their derivatives from 10 mM DMSO stock solutions were added to aliquots to achieve lipid/polyene molar ratios of 50:1 and 10:1. The liposomal suspension was heated and cooled at a constant rate of 0.2 and 0.3 °C/min, respectively. The reversibility of the thermal transitions was assessed by reheating the sample immediately after the cooling step from the previous scan. The temperature dependence of the excess heat capacity was analyzed using Calisto Processing (Setaram, Caluire-et-Cuire, France). The thermograms were characterized by the maximum temperature of the main phase transition (*T_m_*) of DPPC/Chol (90/10 mol%) and the half-width of the main peak on the endotherm (*T*_1/2_), indicating the inverse cooperativity of the melting process.

### 4.13. The Confocal Fluorescence Microscopy of Giant Unilamellar Vesicles

Giant unilamellar vesicles were formed using the electroformation method on a pair of indium tin oxide (ITO) slides with a commercial Nanion Vesicle Prep Pro (Munich, Germany) as previously described [40]. Lipid stock solutions of POPC and POPC/Erg (67/33 mol%) were prepared in chloroform. Fluorescent labeling was achieved by adding the lipid probe Rh-DPPE at a concentration of 1 mol%. Rh-DPPE preferentially associates with the liquid disordered phase (*l_d_*) and is excluded from the ordered phase [39]. The resulting aqueous liposome suspension containing 0.8 mM lipid and 0.5 M sorbitol was divided into 50 mL aliquots. Nata and its derivatives from 1 mM DMSO stock solution were added to aliquots up to 150 μM. The liposome suspension with polyenes was allowed to equilibrate for 30 min at room temperature (25 ± 1 °C). For standard microscopy observation, 10 μL of the resulting liposome suspension, with or without polyenes, was placed on a standard microscope slide and covered by a cover slip. Vesicles were imaged through an oil immersion objective (65 ×/1.4HCX PL) on an Olympus (Hamburg, Germany) microscope. A helium–neon laser with a wavelength of 561 nm was used to excite Rh-DPPE. Temperature during observation was controlled by the air heating/cooling within a thermally insulated camera.

At least 3–4 independent experiments were performed with each tested derivative.

## Figures and Tables

**Table 1:** Antifungal activity of amides of AmB, Nys, and Nata with quaternized groups in comparison with paternal antifungals.

MIC (μg/mL) ^1^												
	4a ^2^	4b ^3^	4c ^4^	AmB	5a ^2^	5b ^3^	5c ^4^	Nys	6a ^2^	6b ^3^	6c ^4^	Nata
*C. albicans* ATCC 10231	2/2 ^1^	1/2	1/1	1/1	8/8	1/2	4/4	2/2	16/16	4/4	16/32	4/4
*C. albicans* 604M	2/2	1/1	1/1	1/2	8/8	2/2	4/8	2/2	32/32	8/8	32/32	4/4
*C. albicans* 8R	2/2	2/2	2/2	2/2	8/16	2/2	4/8	2/4	16/32	4/8	16/32	4/4
*C. parapsilosis* ATCC 22019	2/2	1/2	1/1	1/1	4/8	2/4	2/4	1/2	16/16	4/8	16/32	4/8
*C. parapsilosis* 58L	2/2	1/2	2/2	1/2	8/16	2/4	4/8	2/4	16/32	8/8	32/32	8/8
*C. glabrata* 61L	2/2	1/1	1/1	1/1	4/8	2/2	2/4	2/4	4/4	2/2	4/4	4/4
*C. tropicalis* 3010	2/2	1/1	1/1	1/1	4/8	2/2	2/4	2/4	4/4	2/2	4/4	4/4
*C. krusei* 432M	4/4	2/2	2/2	2/2	16/16	2/4	8/16	2/4	16/16	4/4	16/16	4/4
*A. fumigatus* ATCC 46645	4	4	4	2	8	4	4	8	>32	16	>32	4

^1^ For *Candida spp* strains, the value corresponds to the MIC after 24/48 h of incubation, respectively. ^2^ **4a**, **5a**, **6a**—*N*-((1-methylpyridin-1-ium-**3**-yl)methyl)amides of AmB, Nys, and Nata, respectively. ^3^ **4b**, **5b**, **6b**—*N*-((1-methylpyridin-1-ium-**4**-yl)methyl)amides of AmB, Nys, and Nata, respectively. ^4^ **4c**, **5c**, **6c**—*N*-(2-(trimethylammonio)ethyl)amides of AmB, Nys, and Nata, respectively.

**Table 2:** Cytotoxicity against human cells human embryonic kidney cells (HEK293) and human postnatal fibroblasts (HPF).

IC_50,_ μM		
	HEK293	hFB-hTERT6
**4a**	5.2 ± 0.7	4.2 ± 0.5
**4b**	>50	9.8 ± 1.4
**4c**	14.1 ± 1.8	4.3 ± 0.6
**AmB (1)**	18.2 ± 4.5	2.1 ± 0.3
**5a**	>100	>100
**5b**	>100	>100
**5c**	>100	>100
**Nys (2)**	25.3 ± 3.4	45.6 ± 6.4
**6a**	>500	174.7 ± 21.8
**6b**	443.0 ± 57.0	82.1 ± 10.3
**6c**	>500	144.1 ± 18.2
**Nata (3)**	78.6 ± 9.1	81.0 ± 11.0
**Doxorubicin**	0.11 ± 0.02	0.12 ± 0.03

**Table 3:** Minimum inhibition concentrations (MICs, μg/mL, 24/48 h incubation) and sessile minimum inhibitory concentration (SMIC_50_ and SMIC_80_, μg/mL) of AmB and its derivative **4b** on the planktonic and biofilm phase of growth of *Candida* species.

	MIC, μg/mL	SMIC_50_, μg/mL	SMIC_80_, μg/mL
*C. albicans* 604M			
AmB (**1**)	0.5/1	1.30 ± 0.05	8.9 ± 0.91
**4b**	1/1	1.20 ± 0.03	7.94 ± 1.1
*C. tropicalis* 56-05			
AmB (**1**)	0.5/2	3.10 ± 0.17	9.3 ± 0.52
**4b**	1/1	2.76 ± 0.19	6.4 ± 0.62
*C. parapsilosis* 58L			
AmB (**1**)	0.5/2	1.60 ± 0.06	4.46 ± 0.24
**4b**	1/2	1.60 ± 0.14	4.20 ± 0.24

**Table 4:** Changing the structure of the biofilm under the influence of the studied compounds.

	Biofilm Height, Microns	Biofilm Eradication (% of Control)	The Value of Cell Indentation on the Upper Layers of the Biofilm δ (nm)	Density Reduction (% of Control)
Control	45.2 ± 1.9	-	11.0 ± 1.4	-
AmB (**1**)	37.1 ± 1.6	17.7	13.1 ± 2.6	1.19
**4b**	16.2 ± 1.2	64.4	31.9 ± 8.0	65.5

**Table 5:** The characteristic parameters of the dependence of calcein release from large unilamellar vesicles of indicated composition induced by 5 μM of AmB, Nys, and Nata and their derivatives on time.

	POPC/Chol (67/33 mol%)			POPC/Erg (67/33 mol%)			RF_Erg_/RF_Chol_
Polyene	RF_max_, %	t_1_, min	t_2_, min	RF_max_, %	t_1_, min	t_2_, min	
**1**	6 ± 1	0.6 ± 0.3	12.4 ± 8.1	9 ± 1	0.3 ± 0.1	5.8 ± 1.1	1.5 ± 0.4
**4a**	22 ± 3	0.2 ± 0.1	3.6 ± 1.3	28 ± 4	0.2 ± 0.1	4.1 ± 0.9	1.3 ± 0.4
**4b**	25 ± 5	1.9 ± 0.7	14.1 ± 1.6	18 ± 6	1.2 ± 0.5	5.5 ± 1.9	0.7 ± 0.4
**4c**	14 ± 1	3.1 ± 0.6	9.1 ± 0.8	24 ± 4	0.2 ± 0.1	3.4 ± 0.8	1.7 ± 0.4
**2**	10 ± 2	0.9 ± 0.3	5.7 ± 1.9	19 ± 3	0.7 ± 0.1	5.4 ± 0.5	1.9 ± 0.1
**5a**	4 ± 2	–	–	8 ± 3	0.2 ± 0.1	4.9 ± 0.8	2.0 ± 0.4
**5b**	3 ± 1	–	–	6 ± 2	–	–	2.0 ± 0.3
**5c**	9 ± 1	0.2 ± 0.1	8.1 ± 3.3	16 ± 3	0.7 ± 0.6	4.1 ± 0.9	1.8 ± 0.5
**3**	3 ± 1	–	–	4 ± 2	–	–	1.3 ± 0.3
**6a**	2 ± 1	–	–	2 ± 2	–	–	1.0 ± 0.2
**6b**	5 ± 2	–	–	24 ± 6	5.1 ± 1.1	26 ± 3	4.8 ± 1.5
**6c**	5 ± 2	–	–	5 ± 2	–	–	1.0 ± 0.2

*RF_Chpl_*, *RF_Erg_*—maximal polyene-induced leakage of calcein from unilamellar vesicles composed of POPC/Chol and POPC/Erg, respectively. *t*_1_, *t*_2_—characteristic times referring to fast and slow components, respectively, of two-exponential dependence fitting the time dependence of calcein release.

**Table 6:** Parameters characterizing (ΔT_m_, ΔT_1/2_) the effects of polyenes and their quaternized amides on the thermotropic behavior of DPPC/Erg (90/10 mol.%) liposomes.

Compounds	Lipid/Polyene Ratio	ΔT_m_, °C	ΔT_1/2_, °C
AmB (**1**)	50:1	−0.1 ± 0.1	0
	10:1	0.2 ± 0.1	0.3 ± 0.1
**4a**	50:1	0	0
	10:1	0	0
**4b**	50:1	0	0
	10:1	0	0
**4c**	50:1	0.1 ± 0.1	0.1 ± 0.1
	10:1	0.3 ± 0.1	2.6 ± 0.2
Nys (**2**)	50:1	−0.1 ± 0.1	0
	10:1	0.4 ± 0.2	0.3 ± 0.1
**5a**	50:1	0 ± 0.1	0.2 ± 0.1
	10:1	0.4 ± 0.2	0.4 ± 0.1
**5b**	50:1	0 ± 0.1	0.2 ± 0.1
	10:1	0.2 ± 0.1	0.5 ± 0.1
**5c**	50:1	0.2 ± 0.1	0.2 ± 0.1
	10:1	0.5 ± 0.2	0.7 ± 0.1

ΔTm, ΔT1/2—the changes in the maximum temperature of lipid melting and in the half-width of the peak in the presence of AmB and Nys and its derivatives. Tm and T1/2 of DPPC/Erg (90/10 mol.%) liposomes untreated by polyenes were equal to 40.3 ± 0.1 °C and 1.3 ± 0.2 °C, respectively.

**Table 7:** Characteristic parameters of the single ion-permeable pores induced by different AmB derivatives in DPhPC/Erg (67/33 mol.%) bilayers.

Polyene	G_200 mV_, pS	τ, ms	P_op_
AmB	31.7 ± 2.1	23 ± 4	0.49 ± 0.09
**4a**	20.5 ± 1.1	6 ± 2	0.33 ± 0.05
**4b**	19.2 ± 2.2	6 ± 1	0.29 ± 0.07
**4c**	19.8 ± 1.8	7 ± 2	0.31 ± 0.08

*G*_200 *mV*_—the mean conductance of polyene channels at 200 mV. *τ*—the mean dwell time of polyene pores. *P_op_*—the probability of polyene channels to be open.

## Data Availability

Data are contained within the article and Supplementary Materials.

## Supplementary Materials

The following supporting information can be downloaded at: https://www.mdpi.com/article/10.3390/antibiotics13070608/s1, Table S1: ^1^H and ^13^C spectra assignment for amphotericin and nystatin derivatives **4a**-**4c**, **5a**-**5c**; Table S2: ^1^H and ^13^C spectra assignment for natamycin derivatives **6a**-**6c**; Figures S1–S4: NMR spectra of the AmB derivative **4a**; Figures S5–S8: NMR spectra of the AmB derivative **4b**; Figures S9–S12: NMR spectra of the AmB derivative **4c**; Figures S13–S16: NMR spectra of the Nys derivative **5a**; Figures S17–S20: NMR spectra of the Nys derivative **5b**; Figures S21–S24: NMR spectra of the Nys derivative **5c**; Figures S25–S28: NMR spectra of the Nata derivative **6a**; Figures S29–S32: NMR spectra of the Nata derivative **6b**; Figures S33–S36: NMR spectra of the Nata derivative **6c**.
