# Supplementary material for: A Study on the Effect of Quaternization of Polyene Antibiotics’ Structures on Their Activity, Toxicity, and Impact on Membrane Models
Source: Antibiotics (Basel). 2024 Jun 29;13(7):608. doi: 10.3390/antibiotics13070608 (PMC11274224; doi:10.3390/antibiotics13070608)
Supplement: Supplementary file 1 [file antibiotics-13-00608-s001.zip › antibiotics-3070807-supplementary.pdf]

SUPPORTING INFORMATION

A study on the effect of quaternization of polyene antibiotics' structures on their activity, toxicity, and impact on membrane models

Olga Omelchuk [1*], Anna Tevyashova [1,2], Svetlana Efimova [3], Natalia Grammatikova [1], Elena Bychkova [1], George Zatonsky [1], Lyubov Dezhenkova [1], Nikita Savin [4], Svetlana Solovieva [1], Olga Ostroumova [2], and Andrey Shchekotikhin [1]

[1] Gause Institute of New Antibiotics, 11 B. Pirogovskaya, Moscow, 119021, Russia; instna@mail.ru
[2] School of Science, Constructor University, Campus Ring 1, 28759 Bremen, Germany, info@constructor.university
[3] Institute of Cytology of the Russian Academy of Sciences, 4 Tikhoretsky ave., St. Petersburg, 194064, Russia; cellbio@incras.ru
[4] Research Laboratory of Biophysics, National University of Science and Technology MISiS, 4 p.1 Leninsky Pr., Moscow, 119049, Russia; kancela@misis.ru

* Correspondence: omelchuk.93@mail.ru

LIST OF CONTENTS

Table S1. ^{1}H and ^{13}C spectra assignment for amphotericin and nystatin derivatives 4a-4c, 5a-5c 2
Table S2. ^{1}H and ^{13}C spectra assignment for natamycin derivatives 6a-6c 7
Figures S1-S4. NMR spectra of the AmB derivative 4a. 11
Figure S5-S8. NMR spectra of the AmB derivative 4b. 15
Figure S9-S12. NMR spectra of the AmB derivative 4c. 19
Figure S13-S16. NMR spectra of the Nys derivative 5a. 23
Figure S17-S20. NMR spectra of the Nys derivative 5b. 27
Figure S21-S24. NMR spectra of the Nys derivative 5c. 31
Figure S25-S28. NMR spectra of the Nata derivative 6a. 35
Figure S29-S32. NMR spectra of the Nata derivative 6b. 39
Figure S33-S36. NMR spectra of the Nata derivative 6c. 43

Table S1. ^{1}H and ^{13}C spectra assignment for amphotericin and nystatin derivatives **4a-4c**, **5a-5c**

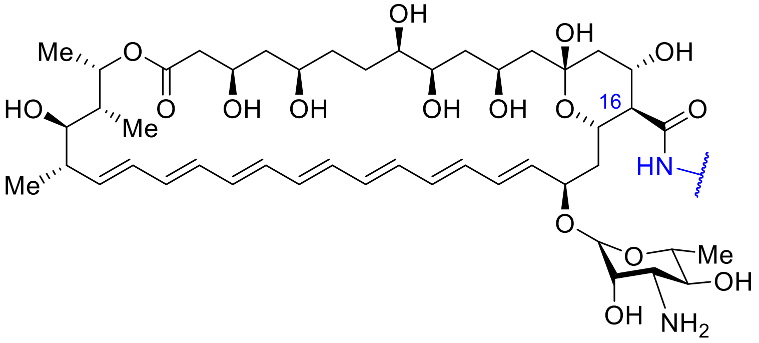

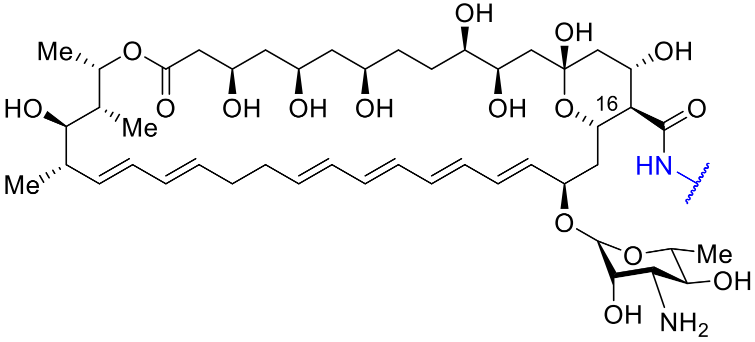

**Compound. ^{13}C/^{1}*H* δ. ppm (*J*, Hz)**							
**Atom**	**4a**	**4b**	**4c**	**Atom**	**5a**	**5b**	**5c**
**Aglycone**							
**1 O-C(O)**	170.5<br>-	172.9<br>-	170.5<br>-	**1 O-C(O)**	170.4<br>-	170.4<br>-	170.4<br>-
**2 CH₂**	42.1<br>*2.17*	43.3<br>*2.22; 2.3*	41.8<br>*2.16*	**2 CH₂**	42.7<br>*2.35; 2.30*	42.7<br>*2.33; 2.29*	42.6<br>*2.34; 2.29*
**3 CH**	66.2<br>4.06	69.0<br>4.19	66.0<br>4.06	**3 CH**	65.8<br>*4.02*	65.8<br>*4.01*	65.8<br>*4.02*
**4 CH₂**	44.6<br>*1.38; 1.32*	44.9<br>*1.42; 1.47*	44.4<br>*1.39; 1.32*	**4 CH₂**	44.2<br>*1.49*	44.2<br>*1.49*	44.3<br>*1.49*
**5 CH**	69.3<br>*3.54*	72.5<br>*3.76*	69.0<br>*3.53*	**5 CH**	67.9<br>*3.81*	67.9<br>*3.81*	67.8<br>*3.80*
**6 CH₂**	35.0<br>*1.40; 1.28*	36.1<br>*1.47*	34.8<br>*1.39; 1.28*	**6 CH₂**	34.3<br>*1.49; 1.38*	34.3<br>*1.47; 1.37*	34.3<br>*1.46; 1.38*
**7 CH₂**	29.0<br>*1.57; 1.27*	30.9<br>*1.47; 1.66*	28.8<br>*1.59; 1.26*	**7 CH**	69.4<br>*3.60*	69.4<br>*3.60*	69.2<br>*3.59*

**8 CH**	73.6<br>*3.10*	75.9<br>*3.25*	73.4<br>*3.09*	**8 CH_2**	42.1<br>*1.69; 1.61*	42.1<br>*1.69; 1.60*	42.2<br>*1.67; 1.57*
**9 CH**	73.7<br>*3.45*	75.2<br>*3.63*	73.6<br>*3.45*	**9 CH_2**	28.4<br>*1.46*	28.4<br>*1.45*	28.4<br>*1.45*
**10 CH_2**	39.5<br>*1.59; 1.36*	41.5<br>*1.71*	39.4<br>*1.57; 1.30*	**10 CH**	73.3<br>*3.27*	73.3<br>*3.27*	73.4<br>*3.24*
**11 CH**	67.6<br>*4.23*	67.8<br>*4.18*	67.4<br>*4.23*	**11 CH**	70.0<br>*3.93*	70.0<br>*3.92*	70.0<br>*3.90*
**12 CH_2**	46.2<br>*1.55*	46.1<br>*1.92*	46.0<br>*1.54*	**12 CH_2**	44.3<br>*1.49; 1.40*	44.3<br>*1.49; 1.39*	44.5<br>*1.46; 1.40*
**13 C-O**	97.1<br>-	106.3<br>-	97.1<br>-	**13 C-O**	97.2<br>-	97.2<br>-	97.2<br>-
**14 CH_2**	44.7<br>*1.93; 1.16*	41.5<br>*1.48; 2.27*	44.5<br>*1.88; 1.11*	**14 CH_2**	44.6<br>*2.00; 1.22*	44.7<br>*1.99; 1.22*	44.8<br>*1.90; 1.18*
**15 CH**	65.3<br>*4.04*	67.5<br>*4.13*	64.8<br>*4.00*	**15 CH**	65.3<br>*4.06*	65.2<br>*4.04*	65.2<br>*3.95*
**16 CH**	56.4<br>*2.18*	58.2<br>*2.21*	56.4<br>*2.07*	**16 CH**	57.2<br>*2.07*	57.2<br>*2.09*	56.8<br>*2.11*
**17 HC-O**	65.3<br>*4.25*	68.1<br>*4.42*	65.0<br>*4.24*	**17 HC-O**	65.6<br>*3.97*	65.6<br>*3.97*	65.9<br>*3.95*
**18 CH_2**	36.9<br>*1.92; 1.50*	40.0<br>*1.77; 2.07*	36.7<br>*1.89; 1.50*	**18 CH_2**	37.6<br>*1.78; 1.55*	37.7<br>*1.77; 1.58*	37.8<br>*1.69; 1.53*
**19 CH-O**	74.2<br>*4.36*	79.4<br>*4.49*	74.4<br>*4.32*	**19 CH-O**	76.4<br>*4.33*	76.4<br>*4.33*	75.9<br>*4.29*
**20 CH**	136.5<br>*5.90*	137.7<br>*6.23*	136.4<br>*5.92*	**20 CH**	133.1<br>*5.61*	133.1<br>*5.62*	133.7<br>*5.60*

**21 CH**	129.3<br>*6.11*	130.4<br>*6.17*	128.9<br>*6.09*	**21 CH**	130.6<br>*6.21*	130.6<br>*6.20*	130.8<br>*6.19*
**22 CH**	133.2<br>*6.30*	133.6<br>*6.23*	133.2<br>*6.39*	**22 CH**	133.1<br>*6.25*	133.0<br>*6.24*	133.3<br>*6.21*
**23 CH**	132.0<br>*6.29*	133.8<br>*6.20*	131.9<br>*6.28*	**23 CH**	131.6<br>*6.24*	131.6<br>*6.23*	131.6<br>*6.22*
**24 CH**	133.8<br>*6.45*	133.9<br>*6.20*	133.7<br>*6.46*	**24 CH**	131.5<br>*6.23*	131.5<br>*6.23*	130.7<br>*6.20*
**25 CH**	132.2<br>*6.31*	134.1<br>*6.21*	132.2<br>*6.30*	**25 CH**	133.3<br>*6.21*	133.0<br>*6.24*	133.3<br>*6.21*
**26 CH**	133.3<br>*6.39*	134.1<br>*6.19*	133.0<br>*6.30*	**26 CH**	130.9<br>*6.12*	130.9<br>*6.10*	131.0<br>*6.11*
**27 CH**	132.4<br>*6.31*	134.3<br>*6.22*	132.0<br>*6.28*	**27 CH**	134.6<br>*5.69*	134.6<br>*5.68*	134.5<br>*5.66*
**28 CH**	133.7<br>*6.34*	134.7<br>*6.17*	133.5<br>*6.34*	**28 CH_2**	31.6<br>*2.19*	31.6<br>*2.14*	31.6<br>*2.19*
**29 CH**	132.4<br>*6.33*	134.8<br>*6.23*	132.2<br>*6.28*	**29 CH_2**	31.6<br>*2.19*	31.6<br>*2.14*	31.7<br>*2.14*
**30 CH**	131.9<br>*6.16*	135.3<br>*6.21*	131.7<br>*6.15*	**30 CH**	131.1<br>*5.50*	131.1<br>*5.49*	131.1<br>*5.49*
**31 CH**	132.1<br>*6.16*	135.3<br>*6.19*	131.9<br>*6.15*	**31 CH**	131.3<br>*5.96*	131.3<br>*5.95*	131.3<br>*5.96*
**32 CH**	131.1<br>*6.07*	133.3<br>*6.17*	131.0<br>*6.07*	**32 CH**	129.3<br>*5.96*	129.3<br>*5.96*	129.3<br>*5.96*

**33 CH**	136.8<br>*5.44*	137.6<br>*5.42*	136.6<br>*5.42*	**33 CH**	135.6<br>*5.53*	135.5<br>*5.51*	135.6<br>*5.52*
**34 CH**	42.3<br>*2.28*	44.1<br>*2.38*	42.1<br>*2.29*	**34 CH**	40.2<br>*2.25*	40.3<br>*2.25*	40.4<br>*2.25*
**35 CH_3**	18.4<br>*1.03*	19.3<br>*1.12*	18.2<br>*1.04*	**35 CH_3**	16.4<br>*0.96*	16.5<br>*0.96*	16.7<br>*0.96*
**36 CH**	77.1<br>*3.09*	79.9<br>*3.23*	76.9<br>*3.09*	**36 CH**	75.9<br>*3.13*	75.9<br>*3.13*	75.9<br>*3.14*
**37 CH**	39.7<br>*1.73*	42.1<br>*1.82*	39.4<br>*1.73*	**37 CH**	39.8<br>*1.79*	39.9<br>*1.80*	39.9<br>*1.81*
**38 CH_3**	12.0<br>*0.91*	12.5<br>*1.01*	11.7<br>*0.92*	**38 CH_3**	12.0<br>*0.86*	12.0<br>*0.86*	12.1<br>*0.86*
**39 CH**	68.9<br>*5.19*	71.3<br>*5.32*	68.6<br>*5.20*	**39 CH**	70.5<br>*5.08*	70.5<br>*5.07*	70.5<br>*5.07*
**40 CH_3**	17.0<br>*1.11*	17.6<br>*1.20*	16.6<br>*1.11*	**40 CH_3**	16.3<br>*1.10*	16.4<br>*1.09*	16.5<br>*1.10*
**41 C=O**	173.0<br>-	175.8<br>-	170.9<br>-	**41 C=O**	172.7<br>-	172.8<br>-	170.9<br>-
**Mycosamine**							
**1' CH**	97.2<br>*4.27*	99.6<br>*4.54*	97.3<br>*4.30*	**1' CH**	97.4<br>*4.48*	97.4<br>*4.47*	98.8<br>*4.36*
**2' CH**	69.1<br>*3.61*	69.4<br>*3.96*	64.6<br>*3.39*	**2' CH**	69.4<br>*3.61*	69.4<br>*3.60*	69.5<br>*3.65*
**3' CH**	56.5<br>*2.29*	57.2<br>*3.08*	56.4<br>*2.35*	**3' CH**	55.2<br>*2.99*	55.2<br>*2.96*	56.4<br>*2.45*

**4' CH**	72.8<br>*2.92*	70.4<br>*3.40*	69.3<br>*3.62*	**4' CH**	68.5<br>*3.24*	68.5<br>*3.24*	72.6<br>*2.94*
**5' CH**	73.3<br>*2.93*	74.6<br>*3.23*	73.0<br>*2.89*	**5' CH**	72.6<br>*3.14*	72.6<br>*3.14*	73.1<br>*3.03*
**6' CH_3**	17.9<br>*1.13*	17.9<br>*1.26*	17.7<br>*1.15*	**6' CH_3**	17.6<br>*1.15*	17.6<br>*1.14*	18.0<br>*1.11*
**Amide moiety**							
**NH**	*9.32*		*9.10*	**NH**	*8.81*<br>t, J=5.9	*8.83*<br>t, J=6.2	*8.87*
	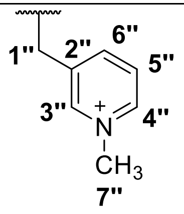	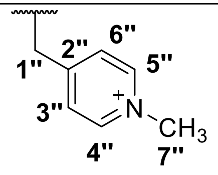	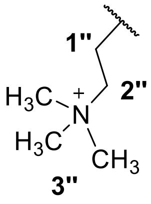		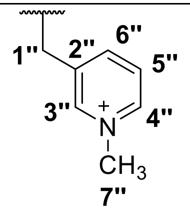	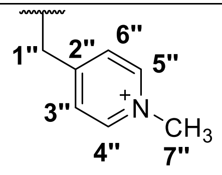	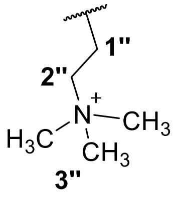
**1′′**	39.2<br>*4.59; 4.41*	43.5<br>*4.93; 4.52*	32.9<br>*3.55; 3.45*	**1′′**	39.1<br>*4.68; 4.32*	41.5<br>*4.74; 4.39*	33.2<br>*3.57; 3.44*
**2′′**	140.5<br>-	161.0<br>-	63.8<br>*3.43*	**2′′**	140.3<br>-	146.3<br>-	63.9<br>*3.40*
**3′′**	143.5<br><br>*8.96 s*	127.0<br>*8.05*<br>d, J=6.7	52.5<br><br>*3.11*	**3′′**	143.3<br><br>*8.86* s	124.9<br>*7.96*<br>d, *J*=6.4	52.7<br><br>*3.10*
**4′′**	143.1<br>*8.49*<br>d, *J*=7.9	146.4<br>*8.79*		**4′′**	143.1<br>*8.45*<br>d, *J*=8.0	144.8<br>*8.87*<br>d, *J*=6.4	
**5′′**	127.2<br>*8.08*<br>dd, J=6.0; 7.9	146.4<br>*8.79*		**5′′**	127.2<br>*8.08*<br>dd, J=8.0; 6.2	144.8<br>*8.87*<br>d, *J*=6.4	

**6′′**	143.7<br>*8.85*<br>d, J=6.1	127.0<br>*8.05*<br>d, J=6.7		**6′′**	143.7<br>*8.87*<br>d, *J*=6.5	124.9<br>*7.96*<br>d, *J*=6.4
**7′′**	48.0<br>*4.32*	48.4<br>*4.36*		**7′′**	48.0<br>*4.31*	47.2<br>*4.29*

Table S2. ^{1}H and ^{13}C spectra assignment for natamycin derivatives **6a**-**6c**

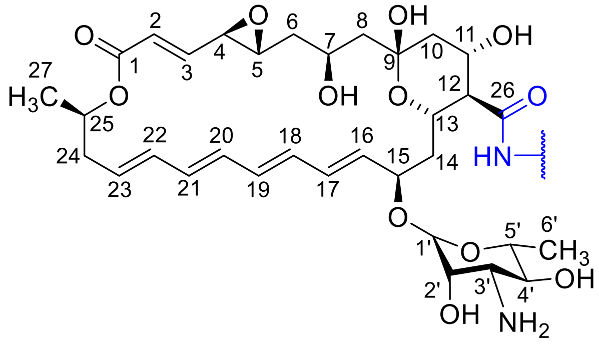

**Atom**	**6a**	**6b**	**6c**
		**Aglycone**	
**1 O-C(O)**	164.4<br>-	164.4<br>-	164.4<br>-
**2 CH**	124.6<br>*6.12*	124.5<br>*6.11*	124.6<br>*6.11*
**3 CH**	144.5<br>*6.27*	144.5<br>*6.26*	144.7<br>*6.25*
**4 HC-O**	53.7<br>*3.24*	53.7<br>*3.22*	53.7<br>*3.22*
**5 HC-O**	57.9	57.9	58.0

	2.76	*2.74*	*2.74*
**6 CH_2**	40.8<br>*1.97; 1.18*	40.8<br>*1.94; 1.16*	40.8<br>*1.93; 1.14*
**7 CH**	66.2<br>*4.15*	66.0<br>*4.13*	66.0<br>*4.12*
**8 CH_2**	46.1<br>*1.62; 1.54*	46.3<br>*1.58; 1.52*	46.4<br>*1.56; 1.48*
**9 C-O**	97.1<br>-	97.1<br>-	97.1<br>-
**10 CH_2**	44.0<br>*1.92; 1.19*	44.6<br>*1.88; 1.19*	44.7<br>*1.83; 1.13*
**11 CH**	65.3<br>*4.10*	65.3<br>*4.02*	65.0<br>*4.00*
**12 CH**	57.0<br>*2.07*	56.6<br>*2.24*	57.0<br>*2.04*
**13 HC-O**	64.9<br>*4.30*	65.1<br>*4.30*	65.2<br>*4.25*
**14 CH_2**	36.0<br>*1.98; 1.57*	37.1<br>*1.95*	37.0<br>*1.89; 1.47*
**15 C-O**	74.5<br>*4.40*	74.4<br>*4.35*	74.5<br>*4.31*
**16 CH**	135.6<br>*5.89*	136.0<br>*5.86*	136.2<br>*5.85*
**17 CH**	128.9<br>*6.14*	128.7<br>*6.11*	128.7<br>*6.10*
**18 CH**	133.2	133.4	133.5

	6.51	*6.49*	*6.50*
**19 CH**	131.5<br>*6.22*	131.5<br>*6.17*	131.3<br>*6.21*
**20 CH**	131.8<br>*6.16*	131.3<br>*6.18*	131.9<br>*6.15*
**21 CH**	131.6<br>*6.20*	131.3<br>*6.22*	131.5<br>*6.17*
**22 CH**	135.4<br>*6.07*	135.4<br>*6.06*	135.5<br>*6.06*
**23 CH**	128.8<br>*5.62*	128.7<br>*5.59*	128.7<br>*5.60*
**24 CH_2**	39.1<br>*2.39; 2.19*	39.1<br>*2.38; 2.20*	39.1<br>*2.38; 2.19*
**25 CH**	69.6<br>*4.68*	69.5<br>*4.68*	69.6<br>*4.67*
**26 C(O)**	172.9<br>-	173.0<br>-	172.9<br>-
**27 CH_3**	20.2<br>*1.26*	20.1<br>*1.25*	20.2<br>*1.25*
**Mycosamine**			
**1' CH**	95.6<br>*4.33*	97.2<br>*4.26*	97.2<br>*4.27*
**2' CH**	67.0<br>*3.82*	69.4<br>*3.60*	69.3<br>*3.64*
**3' CH**	55.4<br>*2.84*	56.6<br>*2.36*	56.6<br>*2.40*

**4' CH**	68.6<br>*3.24*	72.8<br>*2.92*	72.8<br>*2.93*
**5' CH**	72.8<br>*3.07*	73.2<br>*2.99*	73.3<br>*3.03*
**6' CH_3**	17.6<br>*1.18*	17.9<br>*1.14*	18.0<br>*1.14*
	**Amide moiety**		
**NH**	8.94	9.96	9.37
	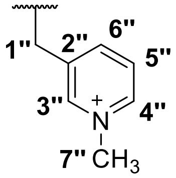	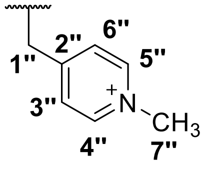	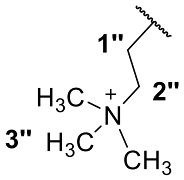
**1′′**	39.1<br>*4.71; 4.35*	40.9<br>*4.44; 4.29*	33.3<br>*3.56; 3.44*
**2′′**	140.3<br>-	144.5<br>-	64.0<br>*3.42*
**3′′**	143.3<br>*8.88*	121.9<br>*7.32*	52.7<br>*3.11*
**4′′**	143.9<br>*8.89*	149.3<br>*8.49*	
**5′′**	127.3<br>*8.10*	149.3<br>*8.49*	
**6′′**	143.0<br>*8.45*	121.9<br>*7.32*	
**7′′**	48.0<br>*4.32*	45.1<br>*4.33*	

LCTA-3646

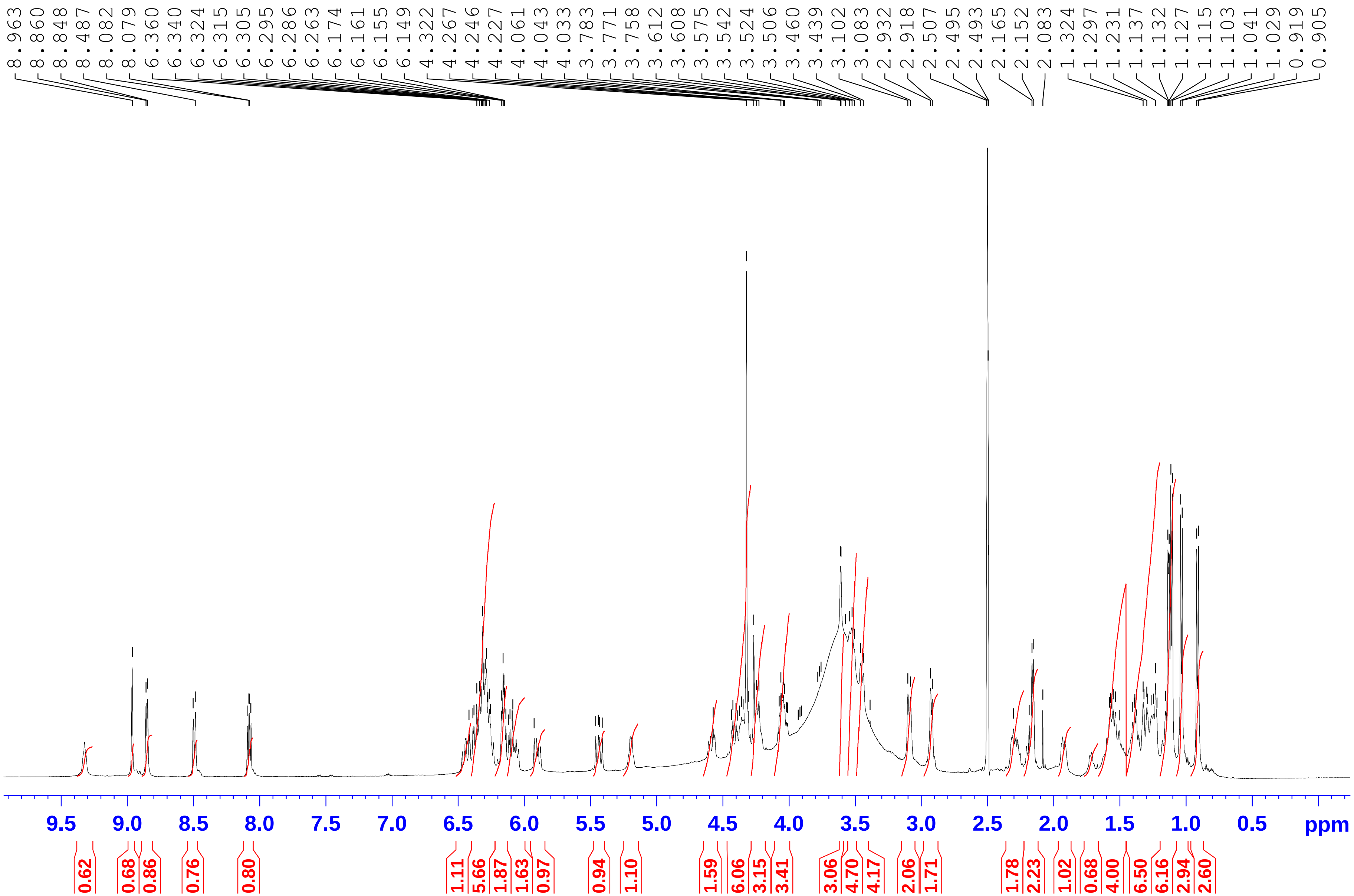

Figure S1. ^{1}H NMR spectra of the AmB derivative **4a.**

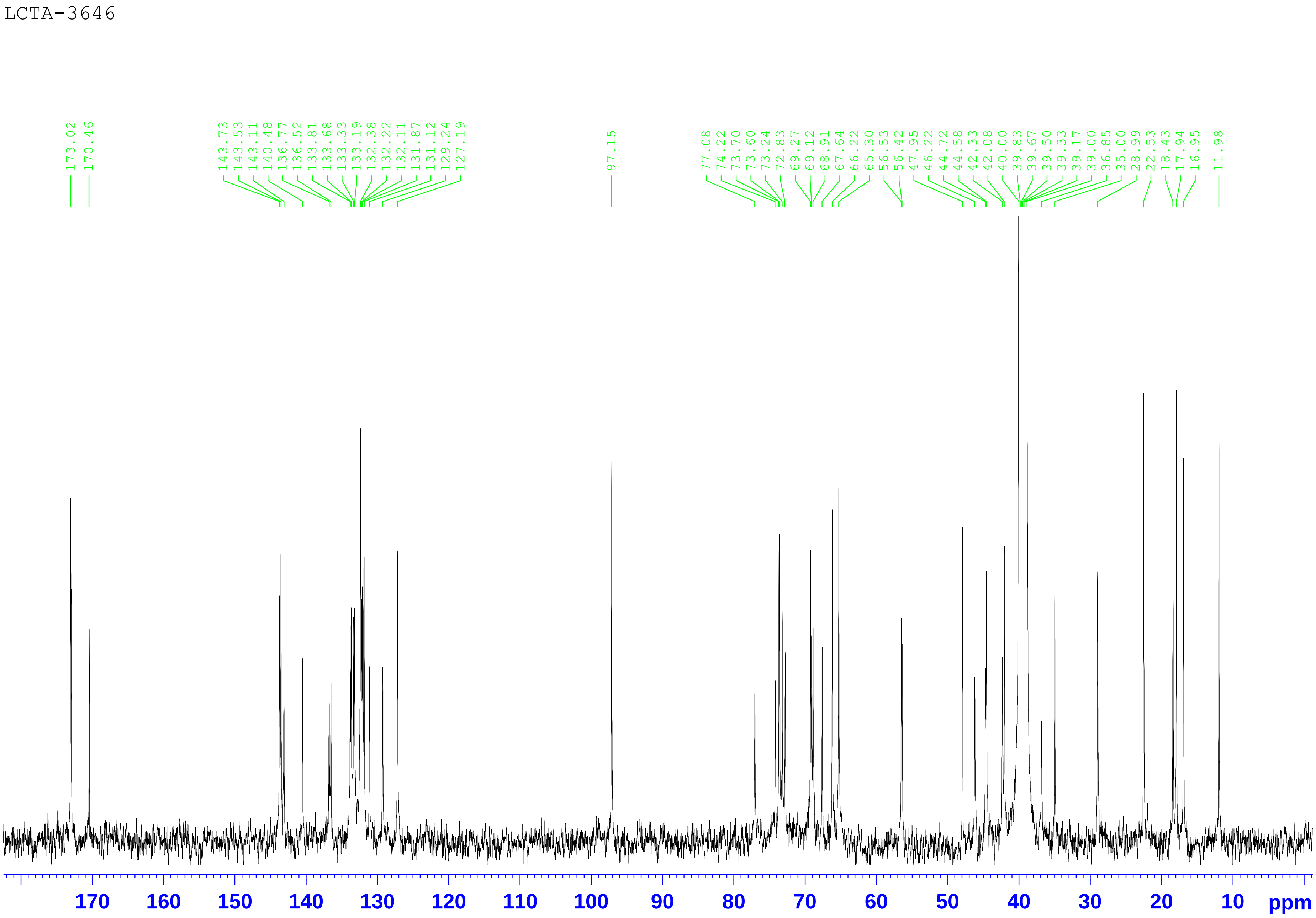

Figure S2. ^{13}C NMR spectra of the AmB derivative **4a.**

LCTA-3646 COSY

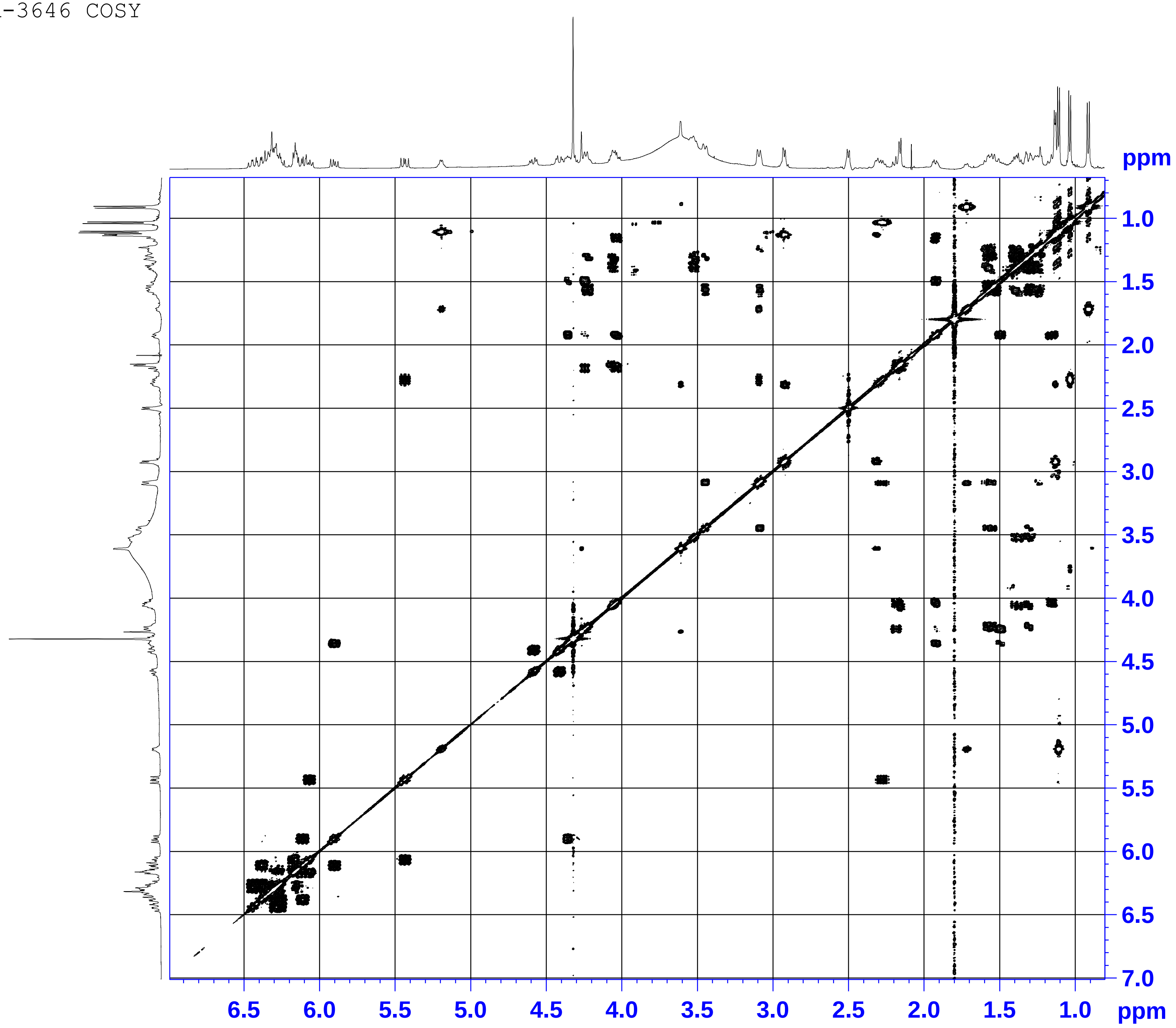

Figure S3. ^{1}H-^{1}H COSY spectra of the AmB derivative **4a**.

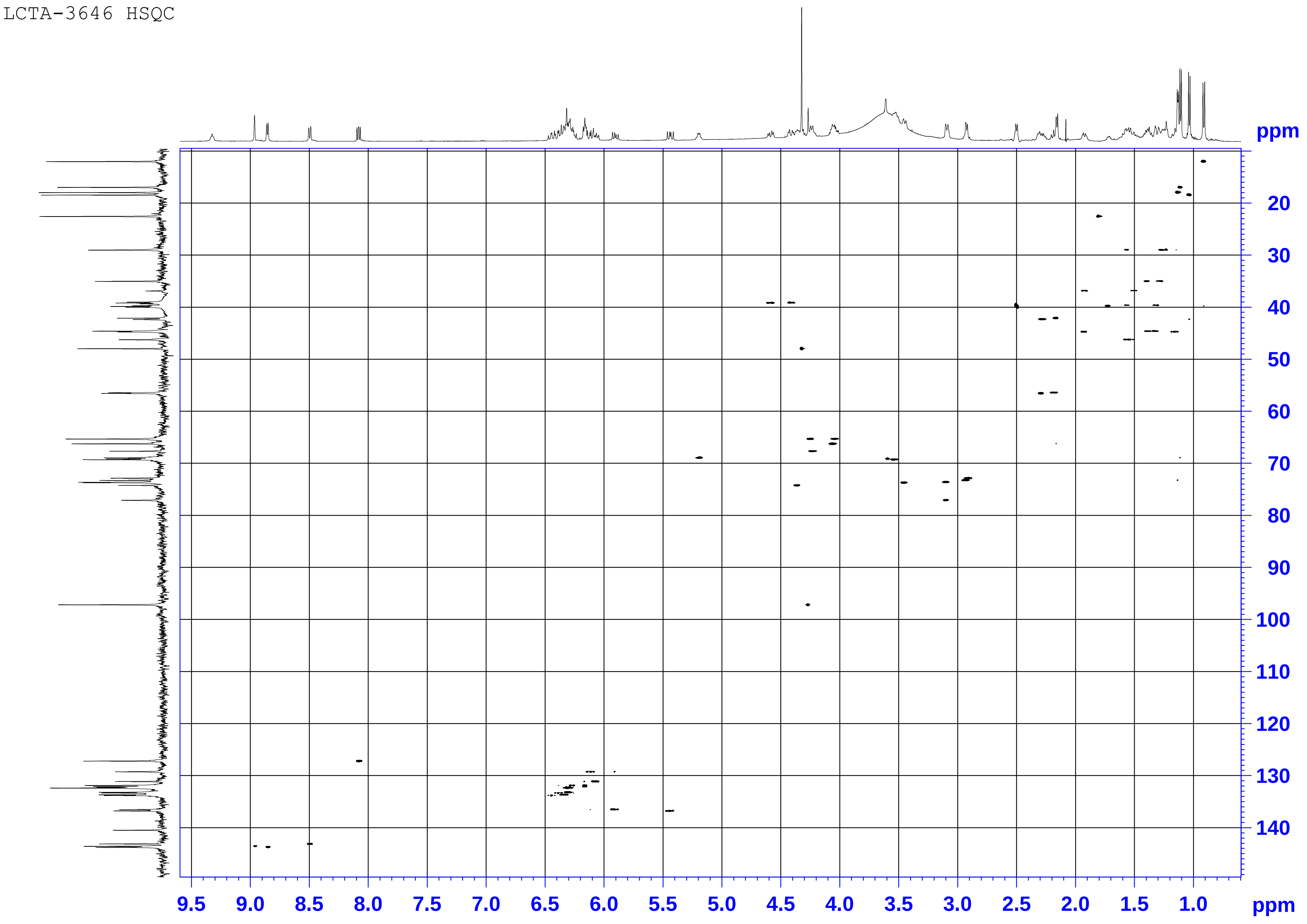

Figure S4. ^{1}H-^{13}C HSQC NMR spectra of the AmB derivative **4a**.

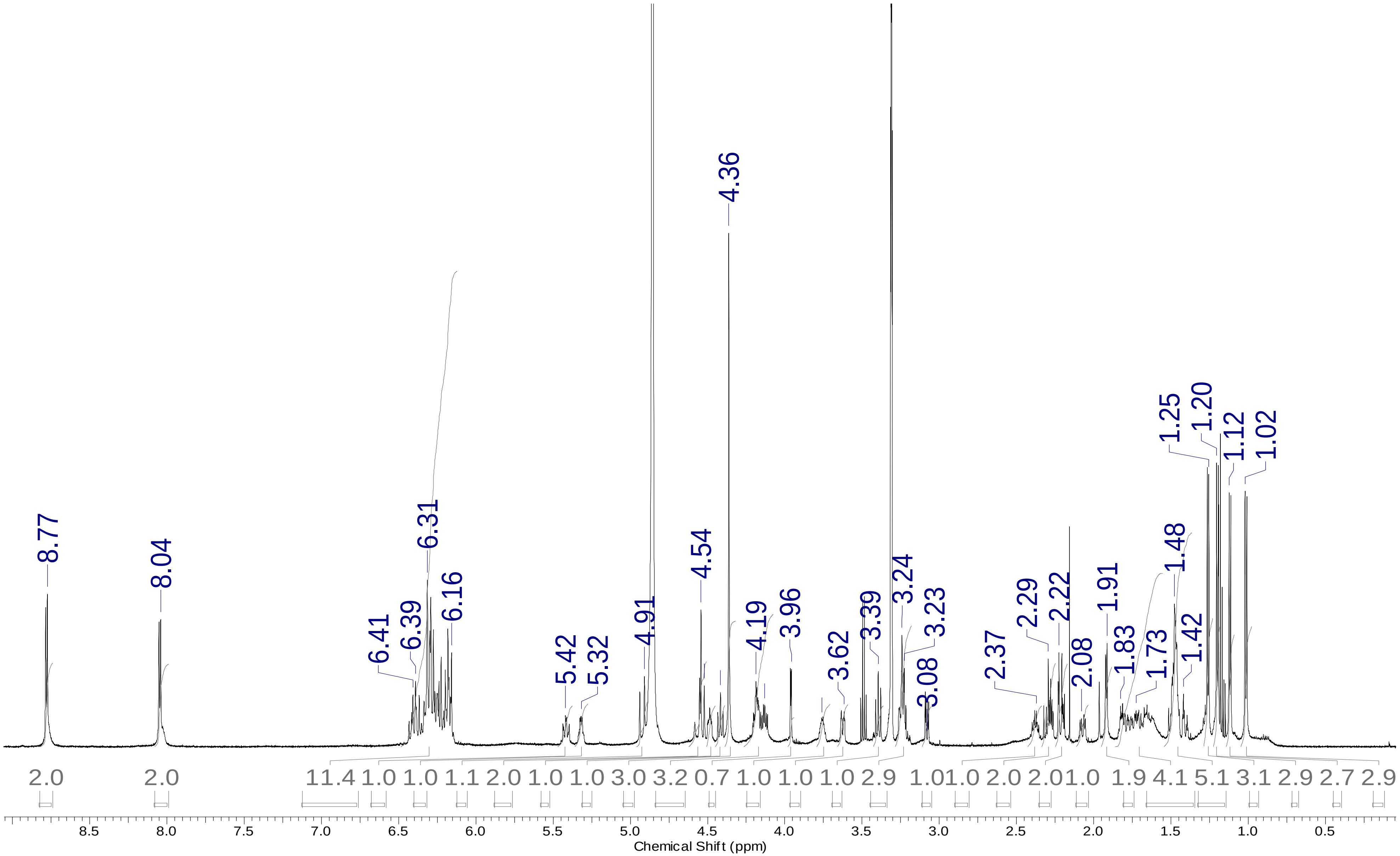

Figure S5. ^{1}H NMR spectra of the AmB derivative **4b.**

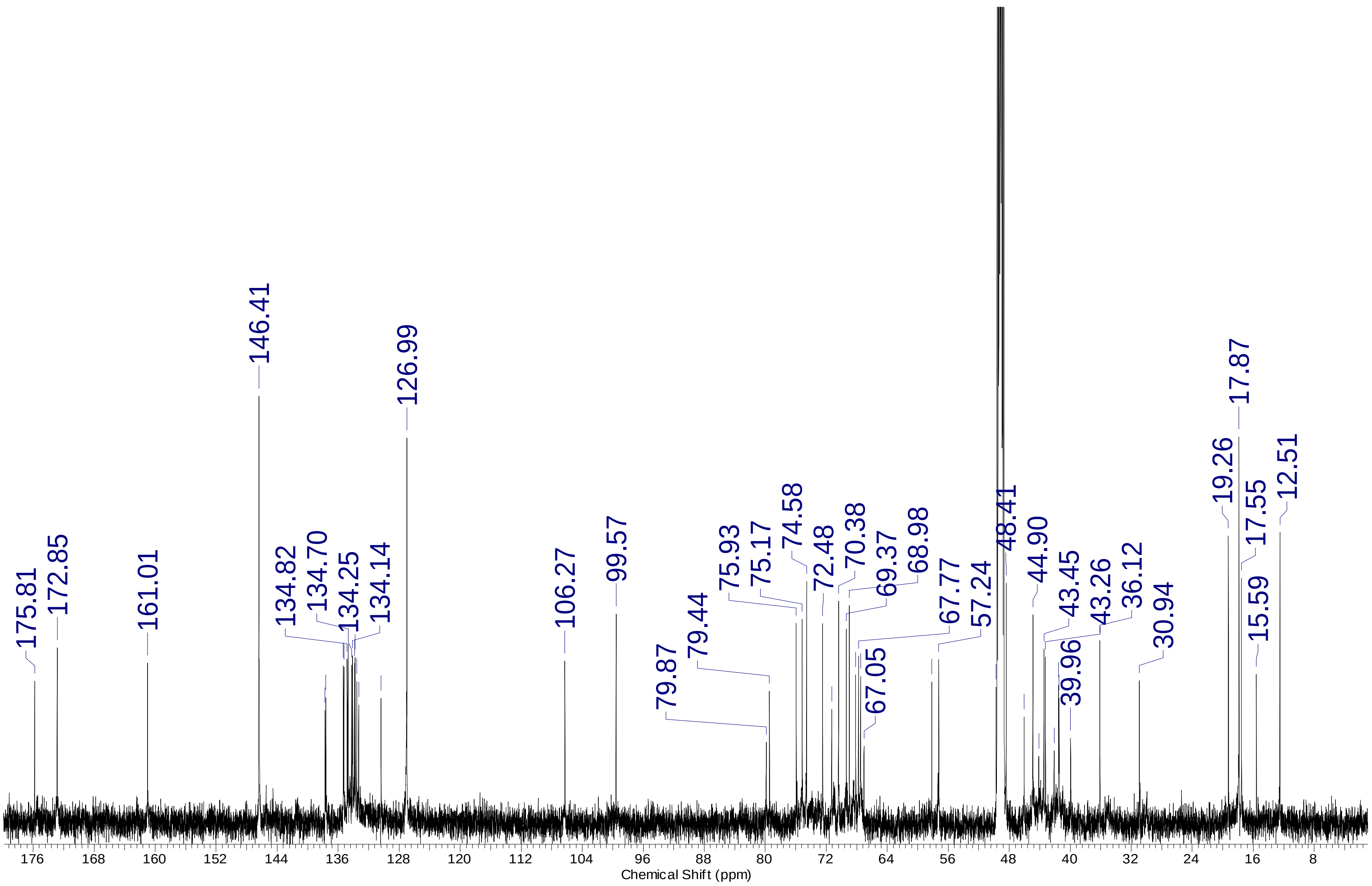

Figure S6. ^{13}C NMR spectra of the AmB derivative **4b.**

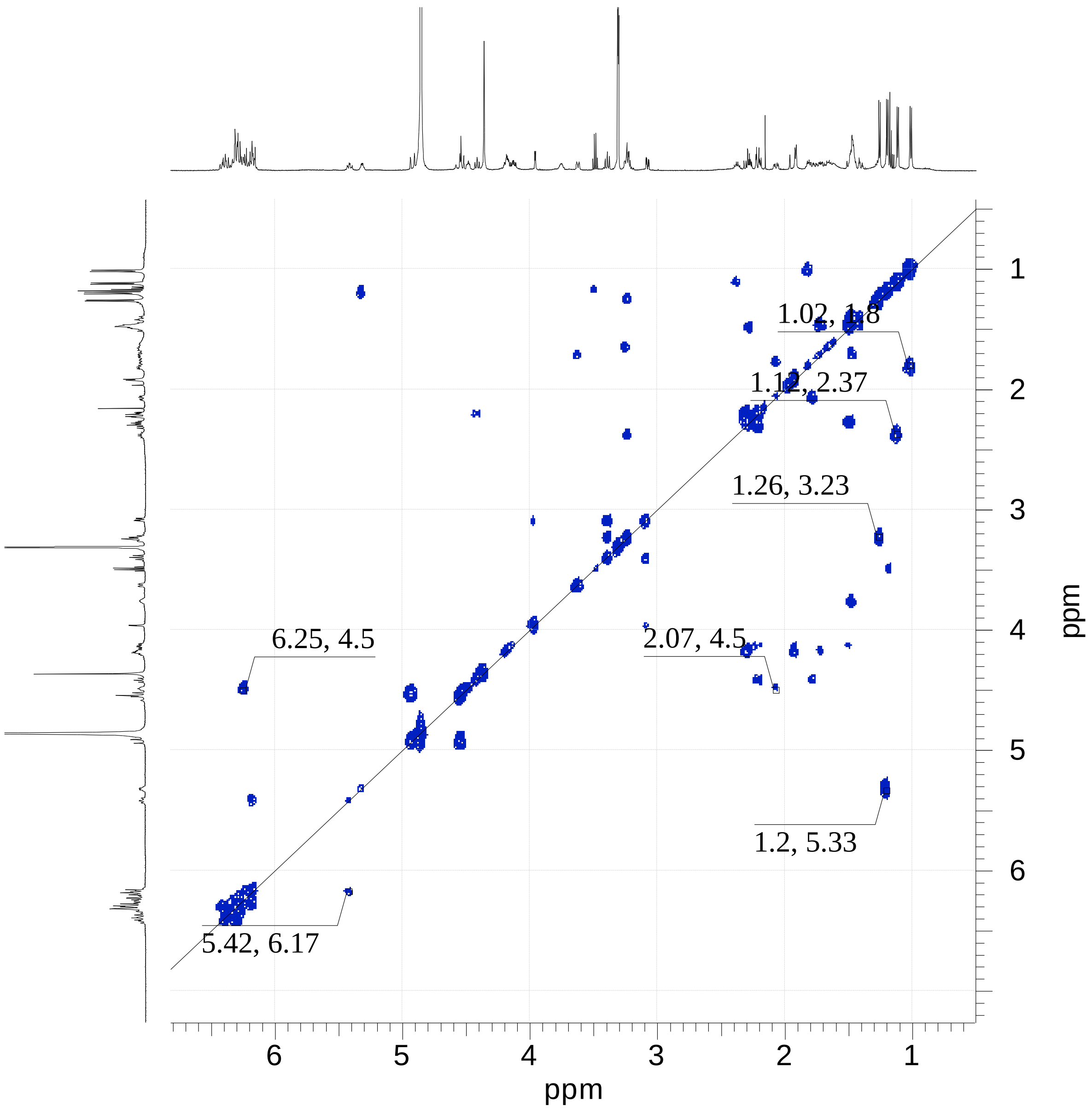

Figure S7. ^1H-^1H COSY spectra of the AmB derivative **4b**.

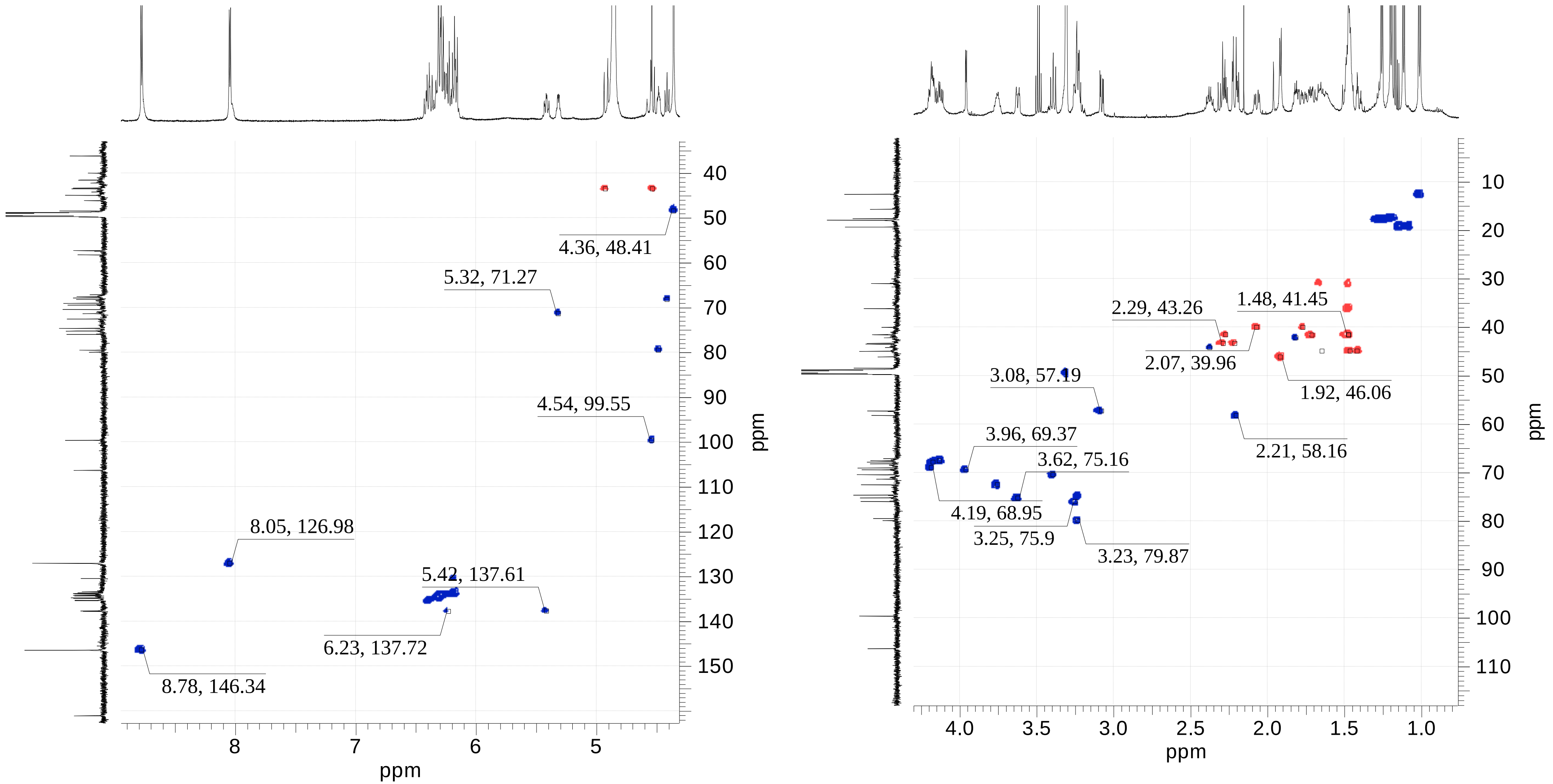

Figure S8. ^{1}H-^{13}C HSQC NMR spectra of the AmB derivative **4b**.

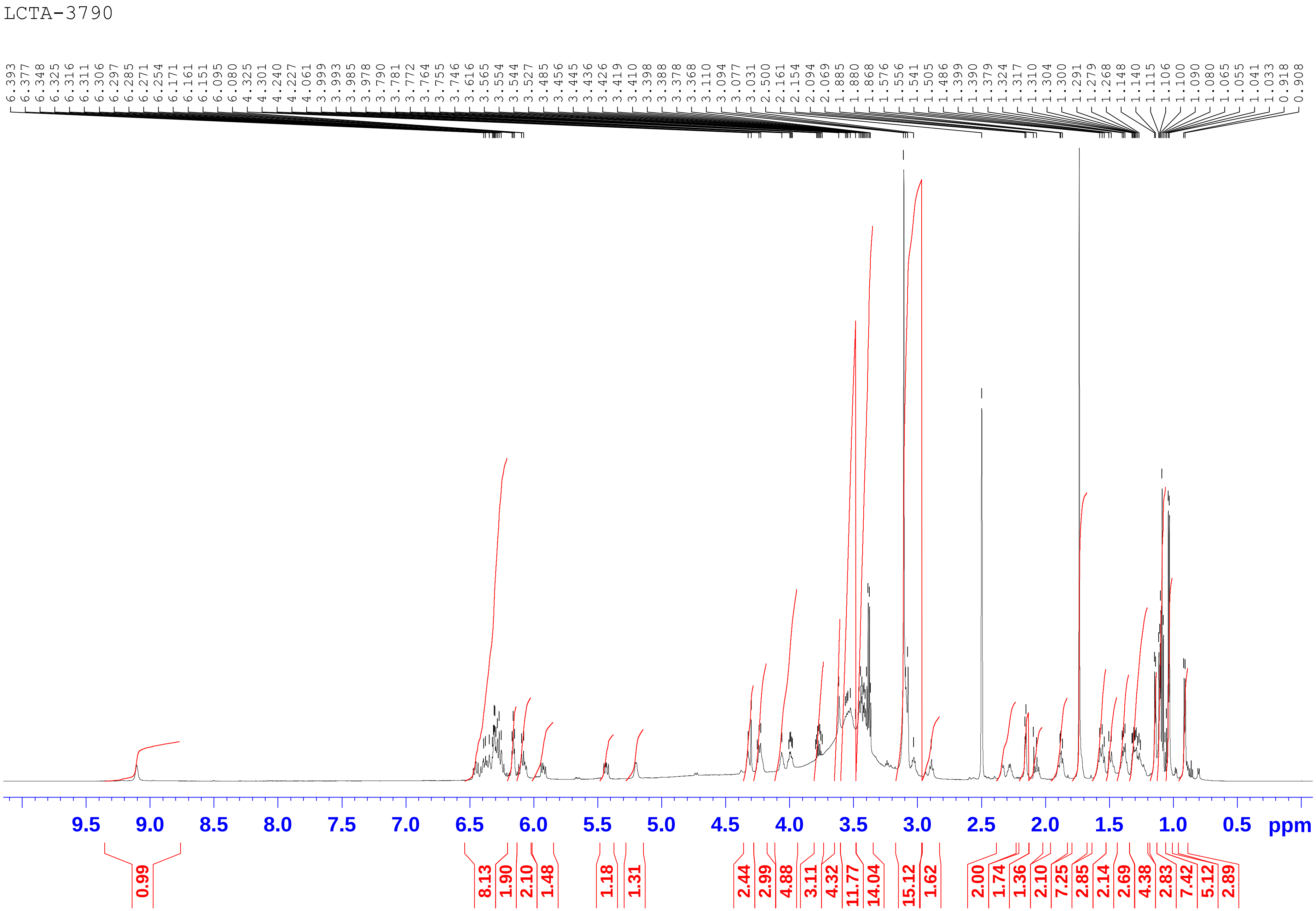

Figure S9. ^{1}H NMR spectra of the AmB derivative **4c.**

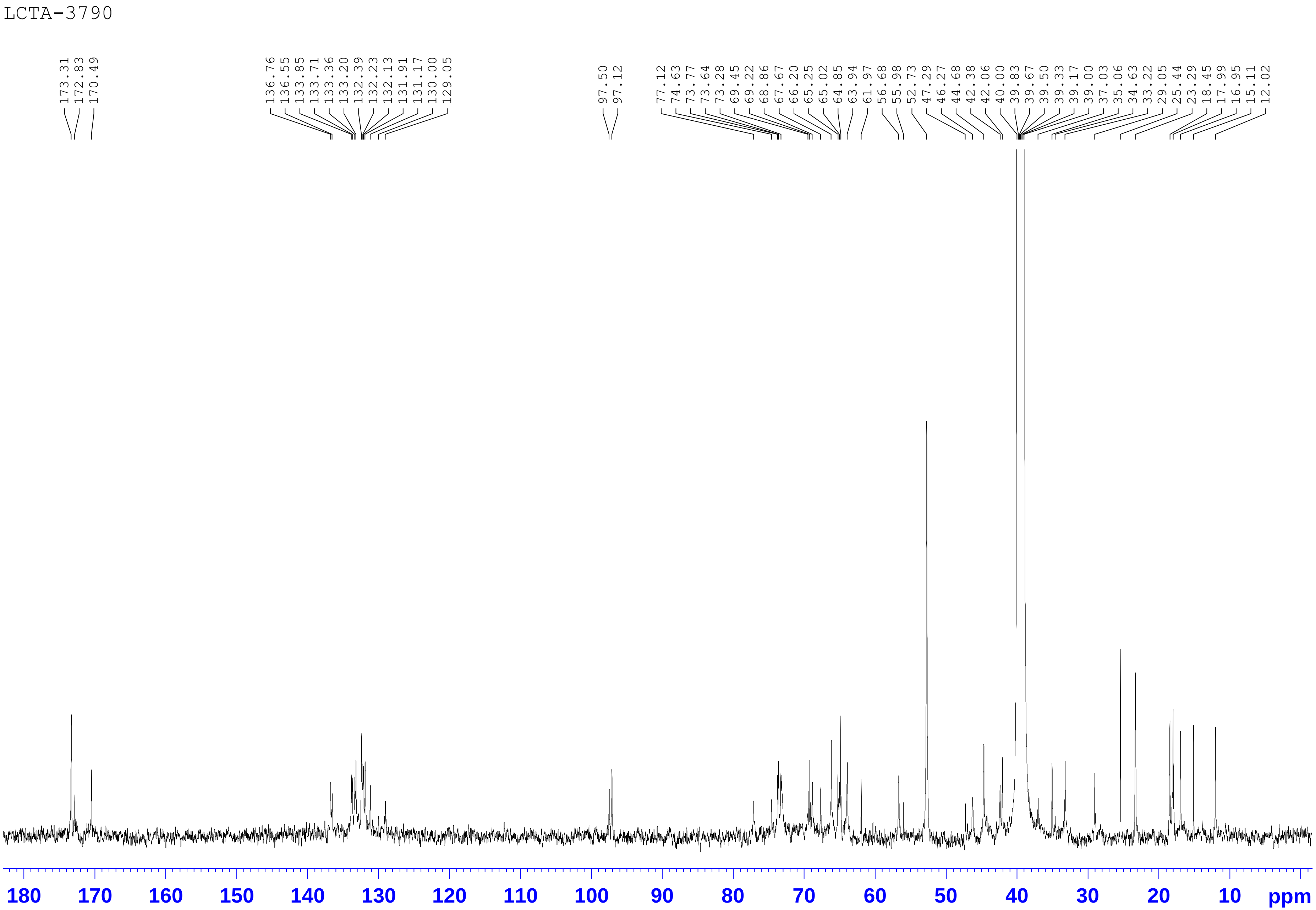

Figure S10. ^{13}C NMR spectra of the AmB derivative **4c.**

LCTA-3790 COSY

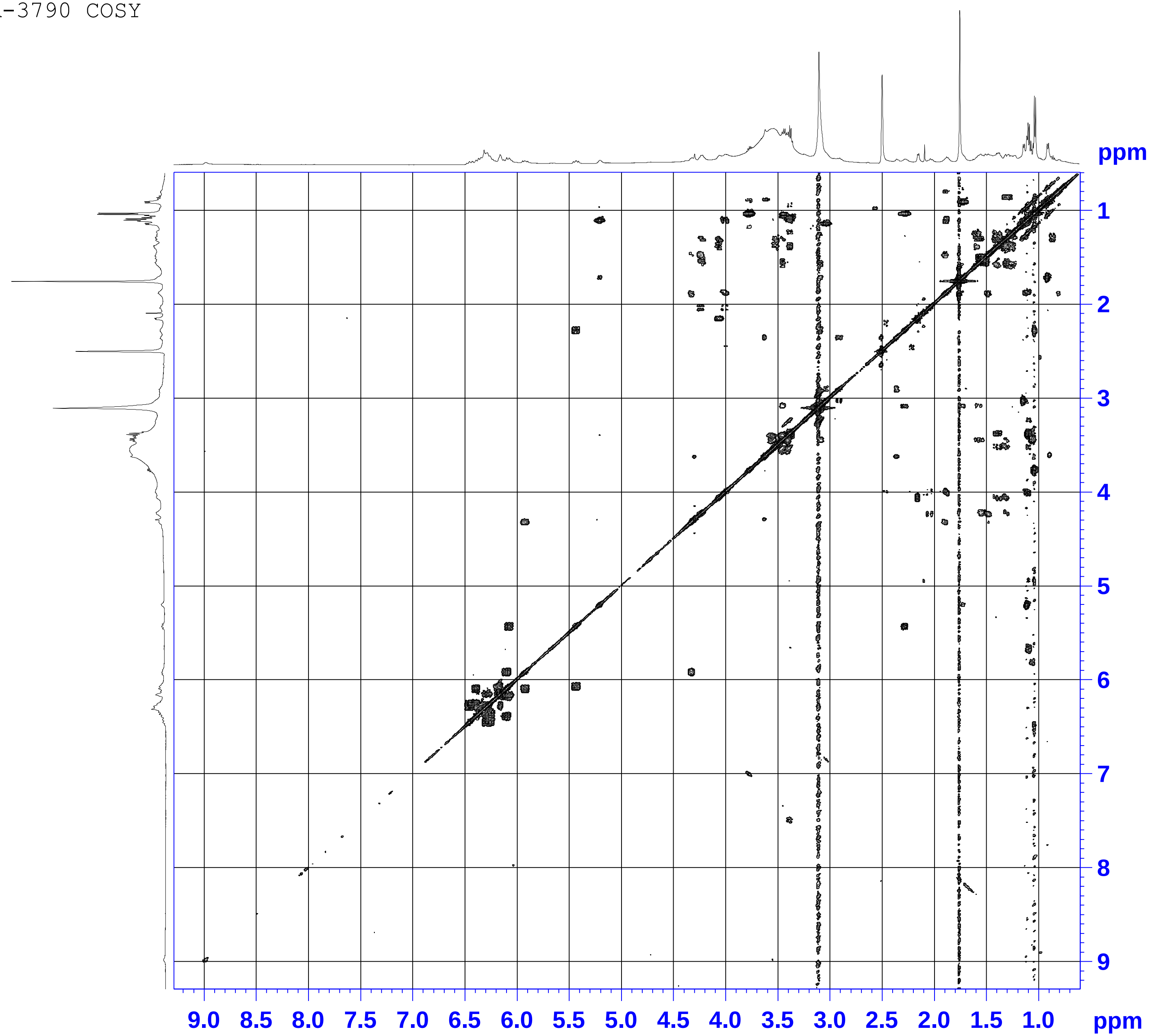

Figure S11. ^{1}H-^{1}H COSY spectra of the AmB derivative **4c**.

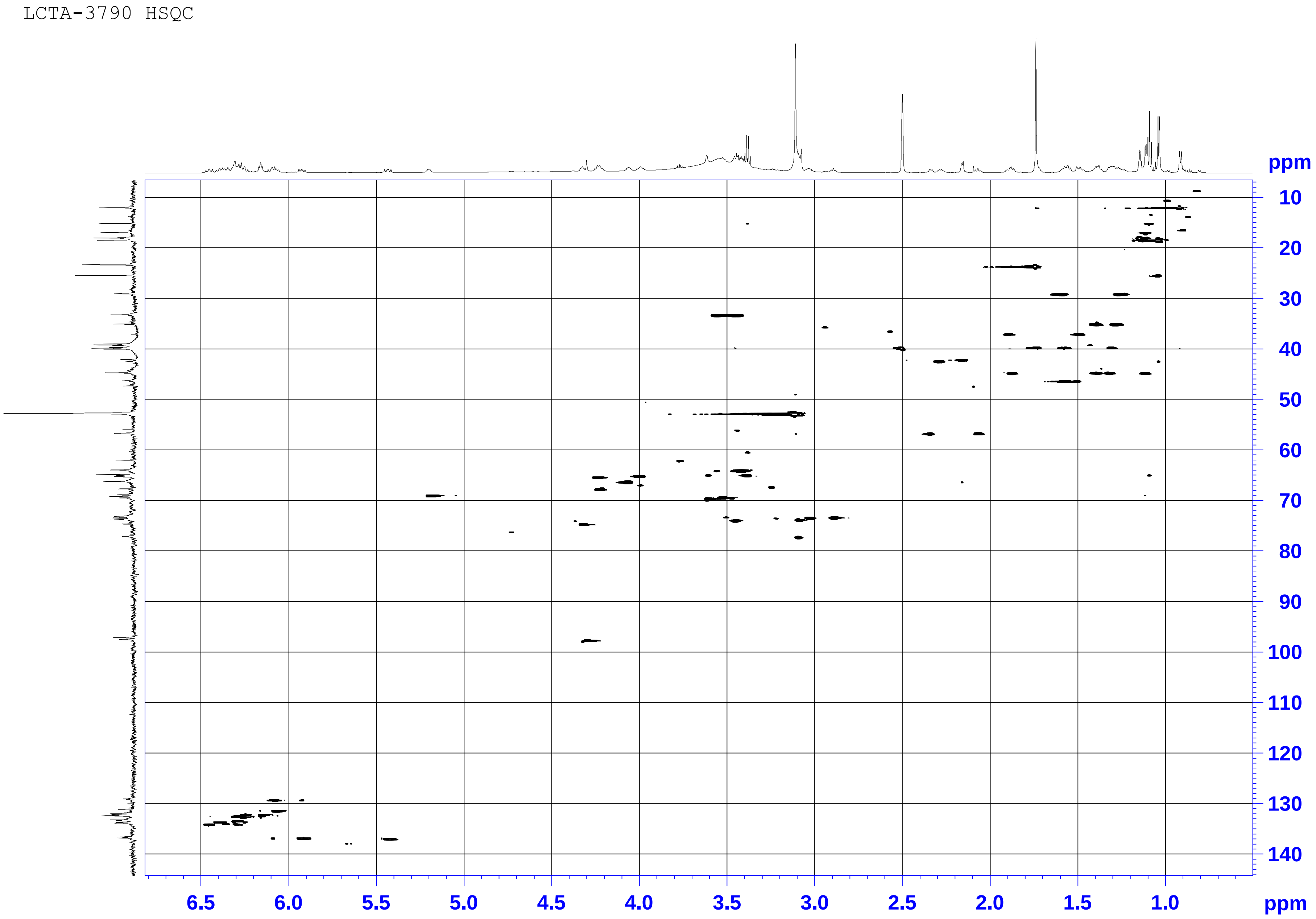

Figure S12. ^{1}H-^{13}C HSQC NMR spectra of the AmB derivative **4c**.

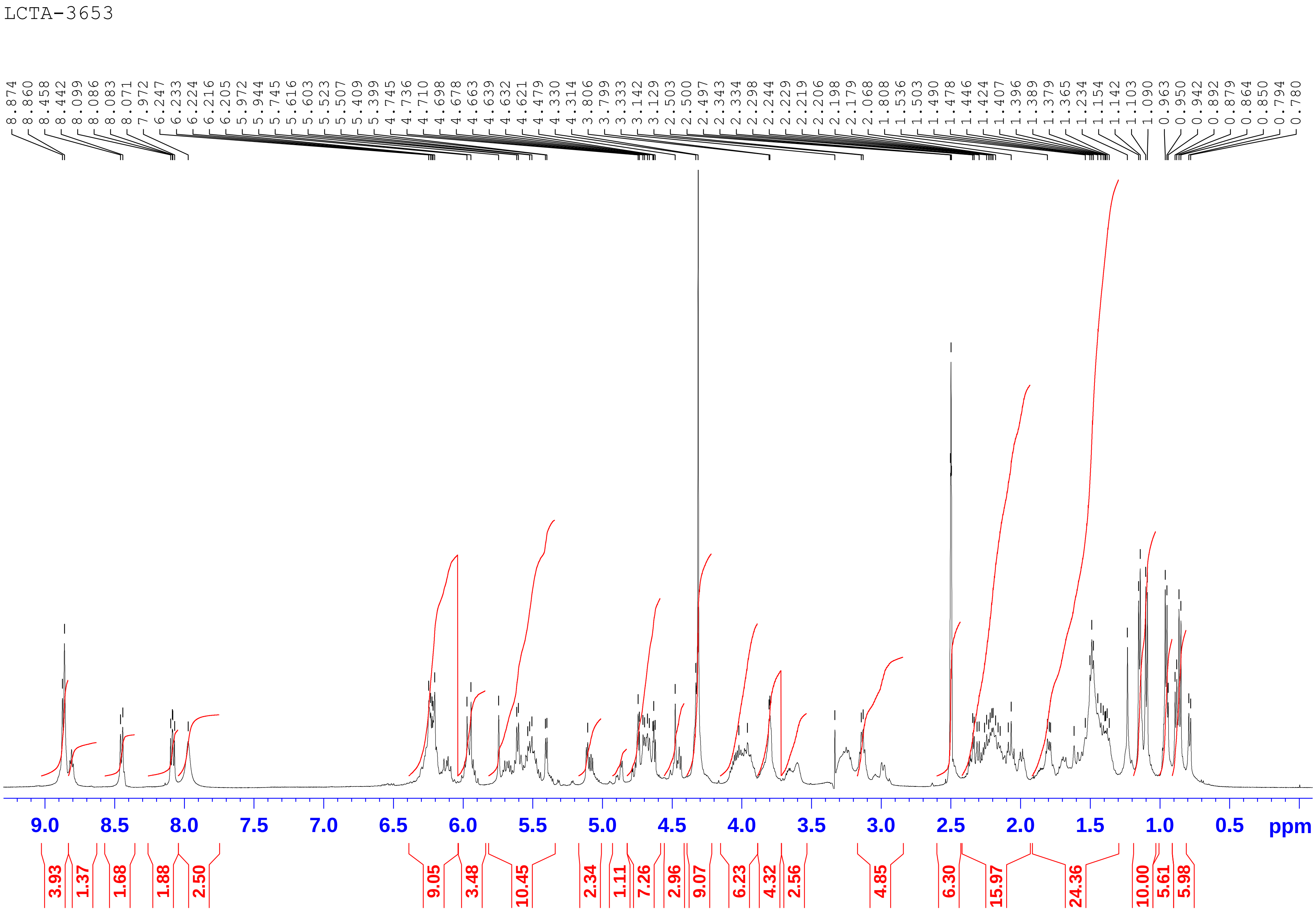

Figure S13. ^{1}H NMR spectra of the Nys derivative **5a**.

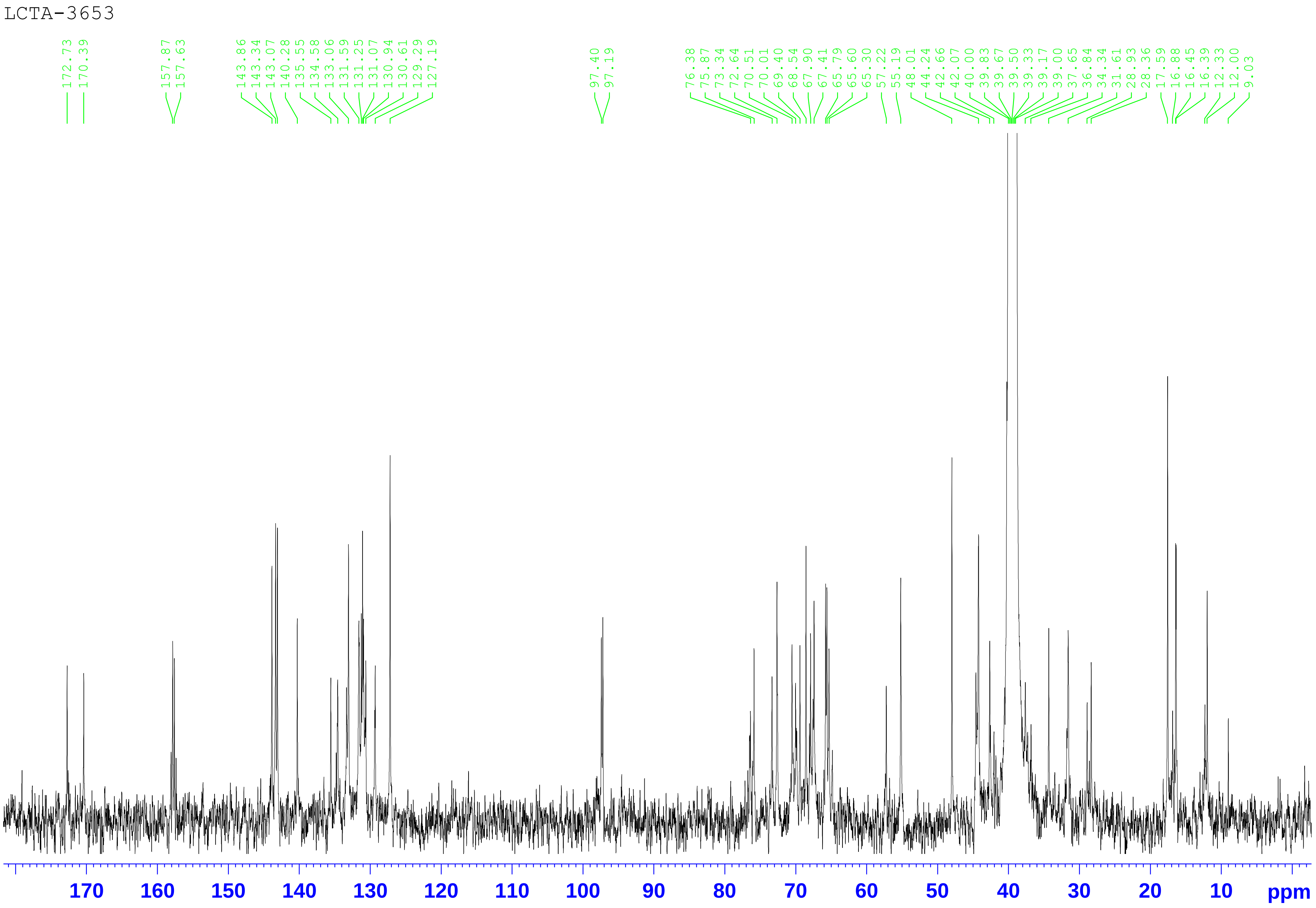

Figure S14. ^{13}C NMR spectra of the Nys derivative **5a**.

LCTA-3653 COSY

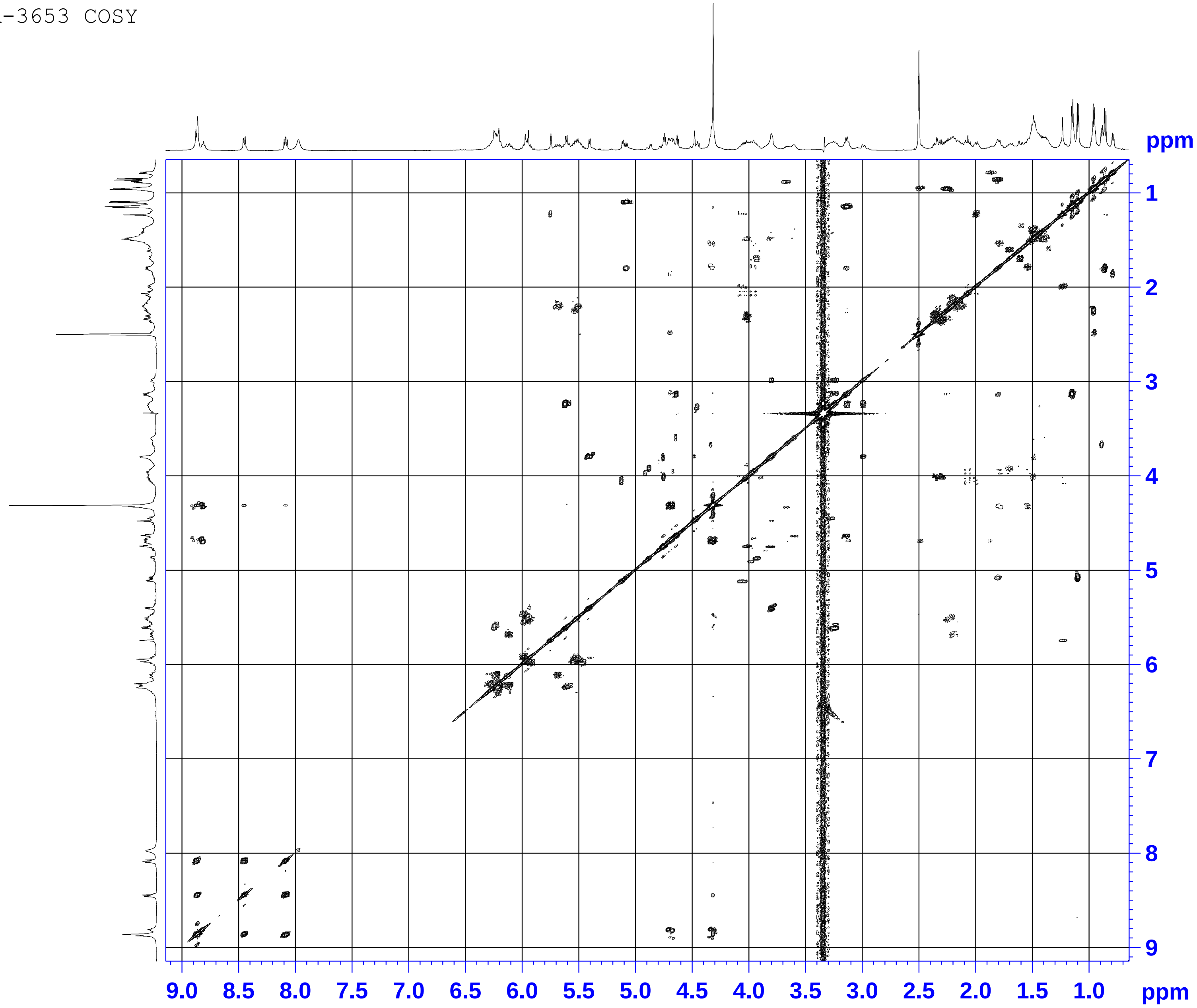

Figure S15. ^{1}H-^{1}H COSY spectra of the Nys derivative **5a**.

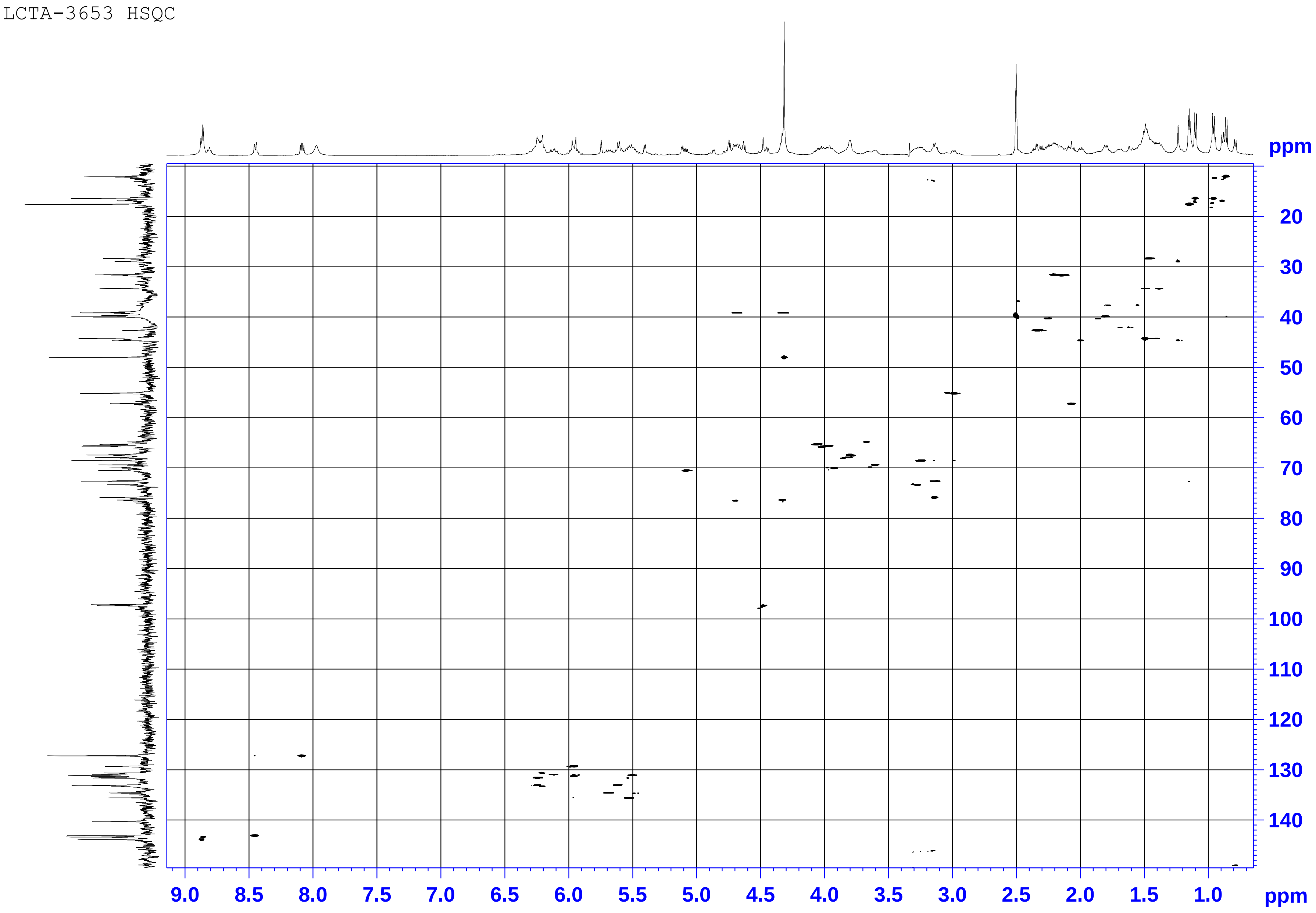

Figure S16. ^{1}H-^{13}C HSQC NMR spectra of the Nys derivative **5a**.

LCTA-3654

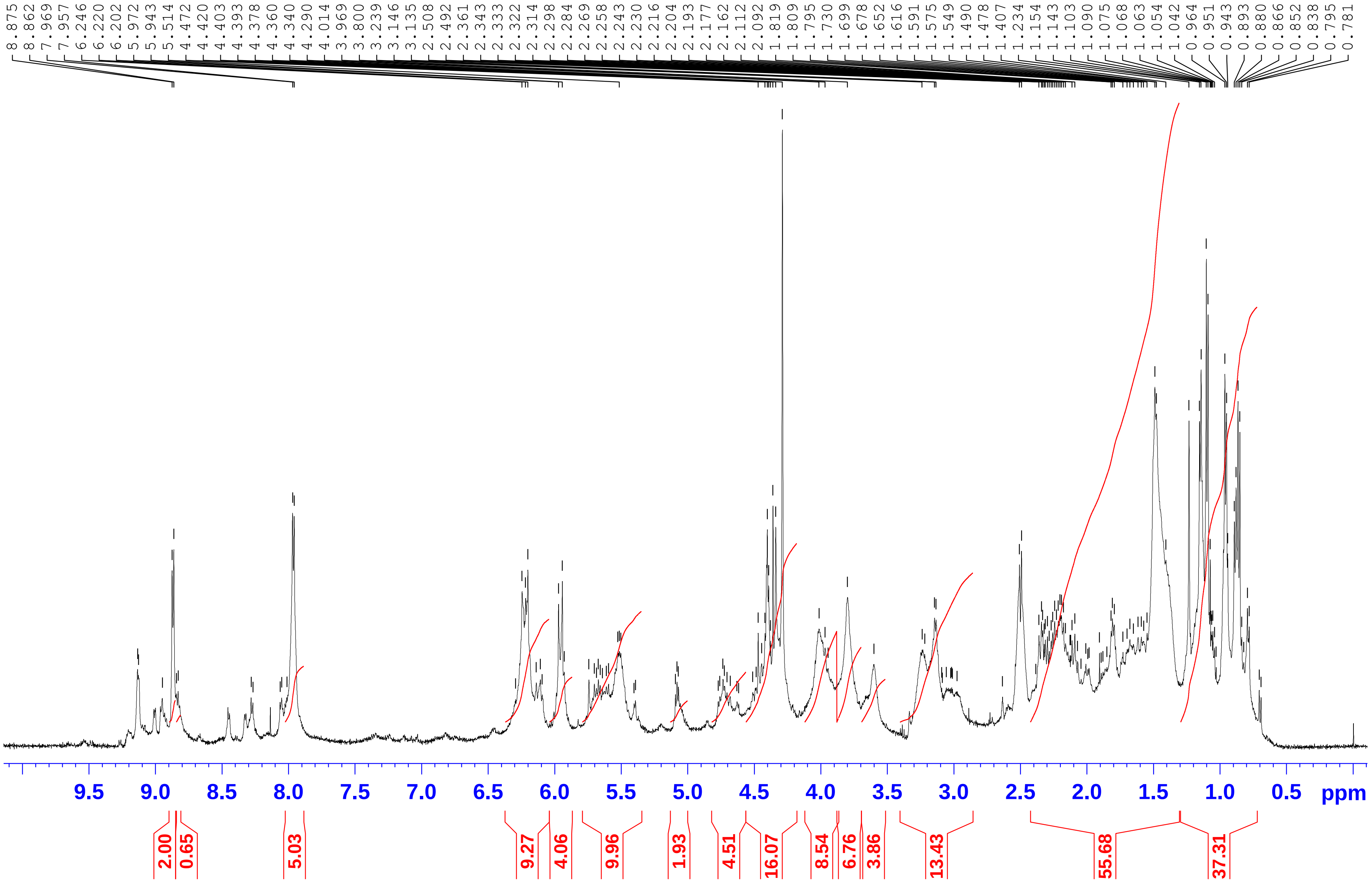

Figure S17. ^{1}H NMR spectra of the Nys derivative **5b**.

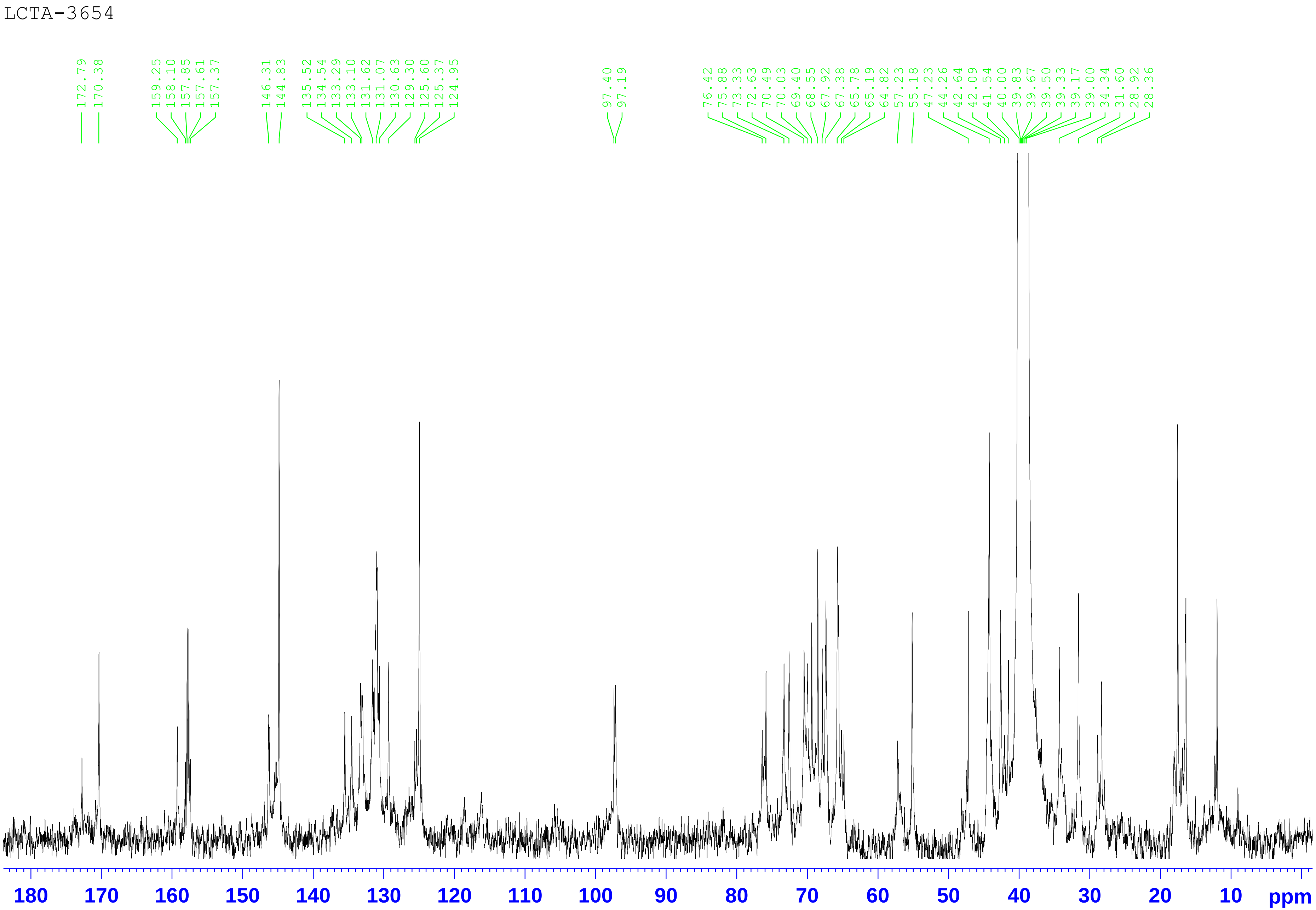

Figure S18. ^{13}C NMR spectra of the Nys derivative **5b**.

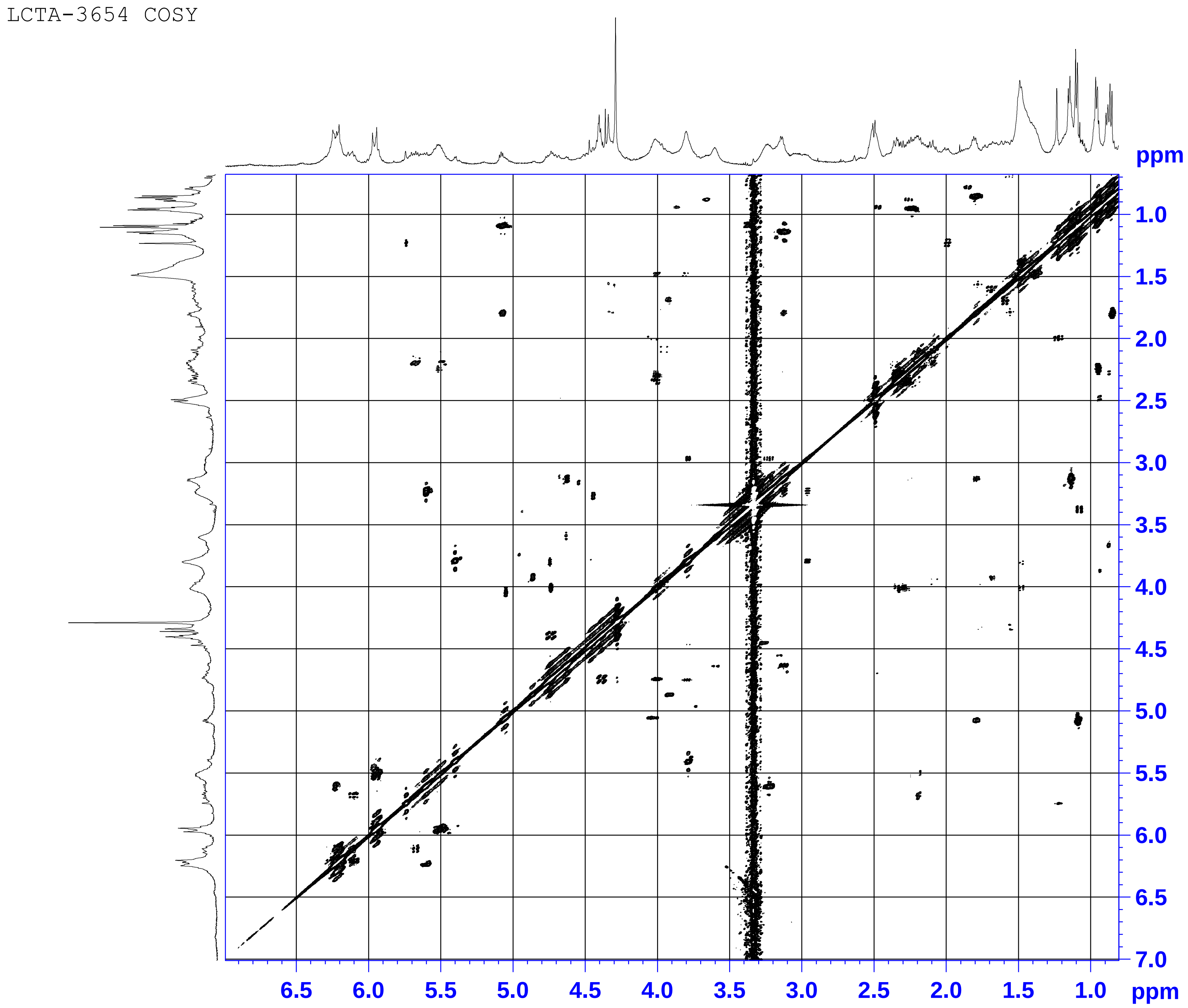

Figure S19. ^{1}H-^{1}H COSY spectra of the Nys derivative **5b**.

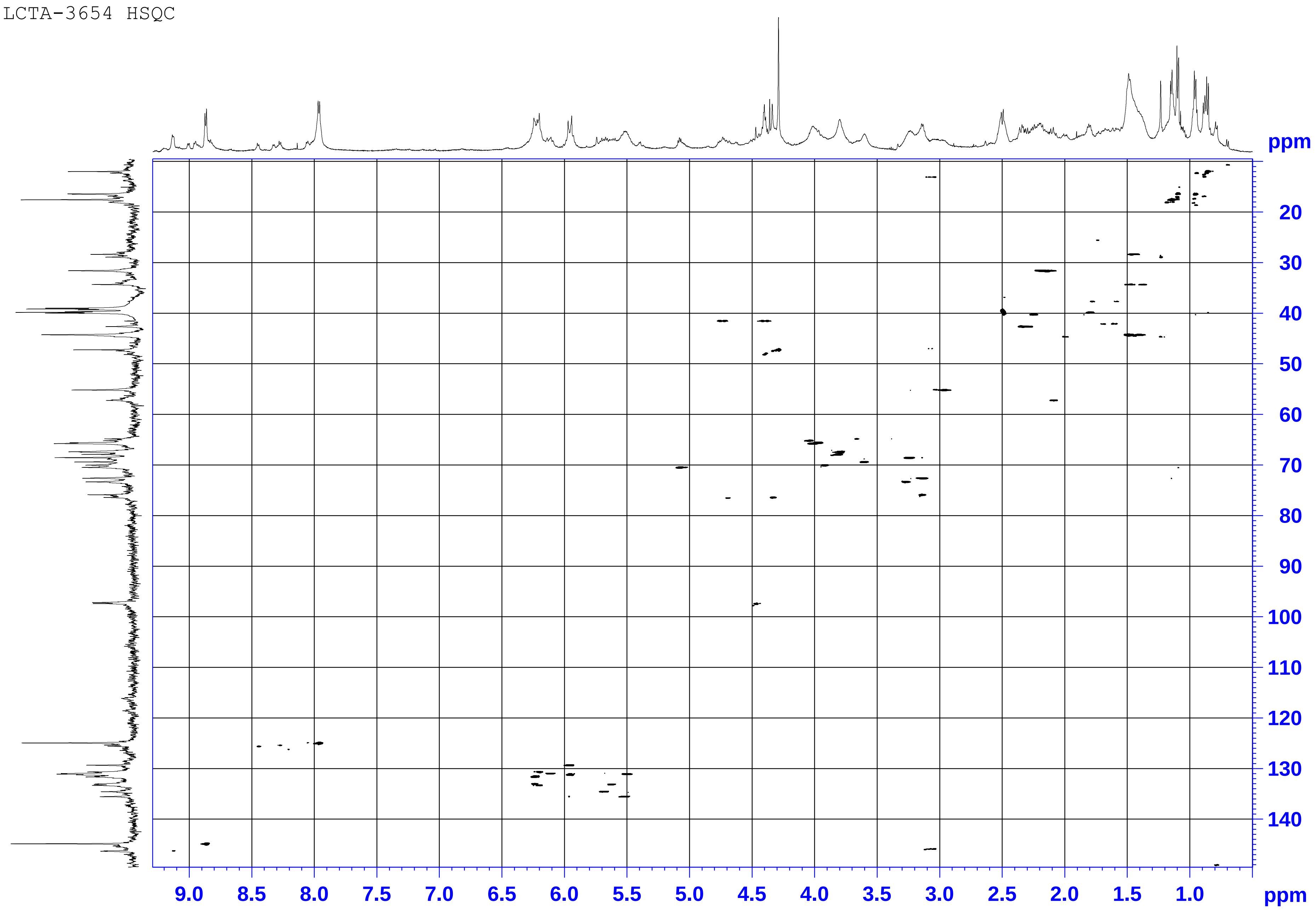

Figure S20. ^{1}H-^{13}C HSQC NMR spectra of the Nys derivative **5b**.

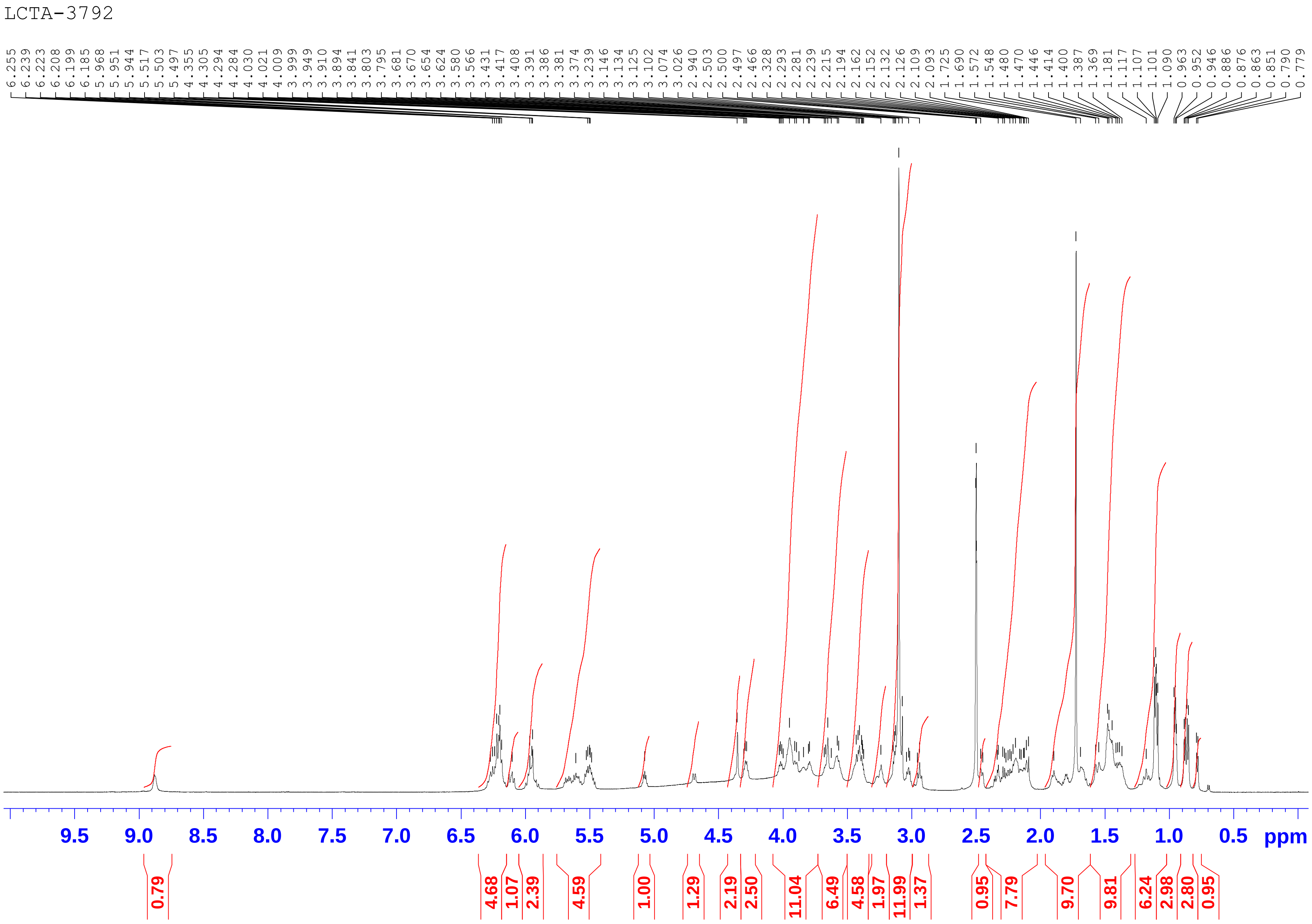

Figure S21. ^{1}H NMR spectra of the Nys derivative **5c**.

LCTA-3792

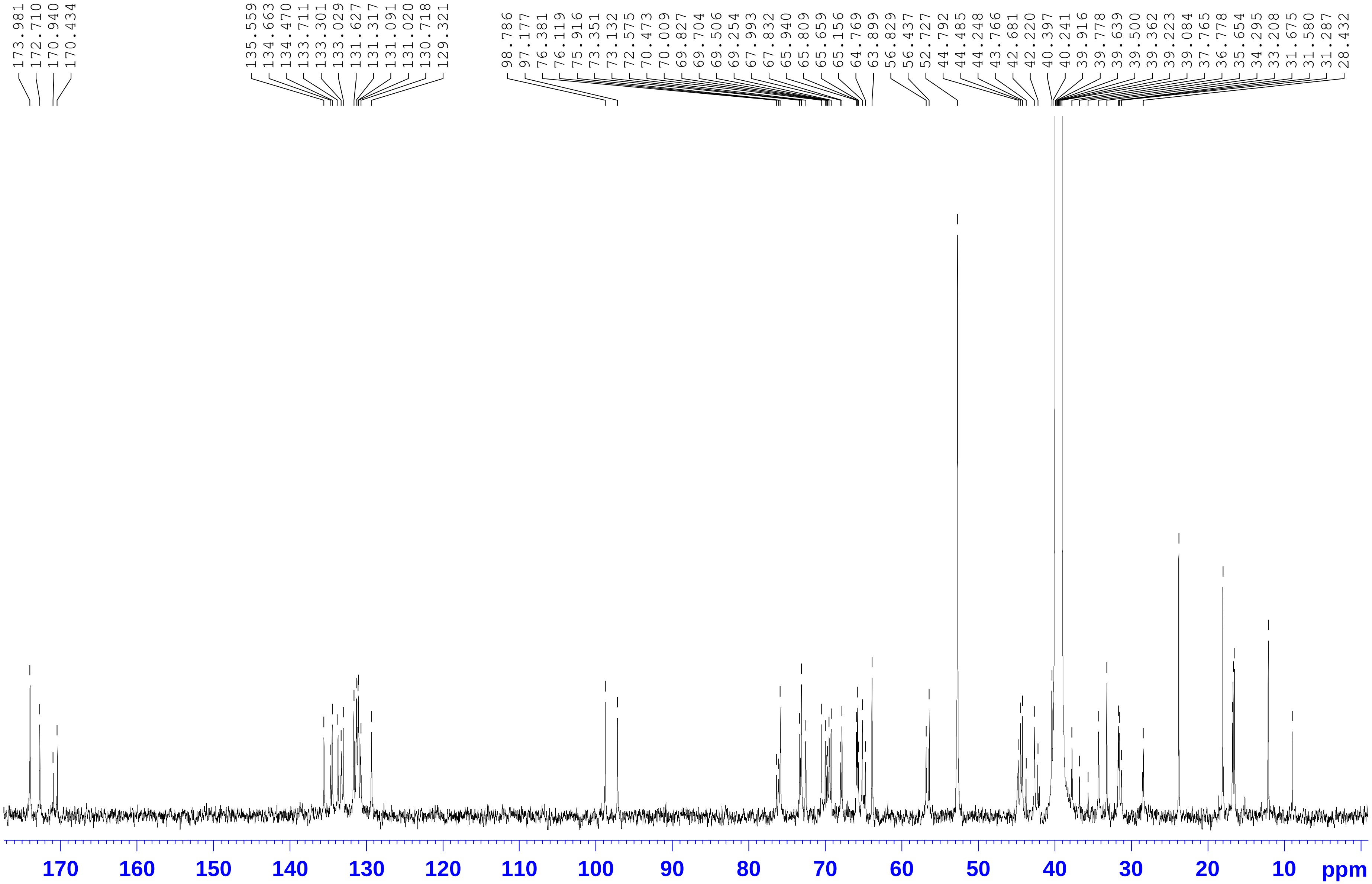

Figure S22. ^{13}C NMR spectra of the Nys derivative **5c**.

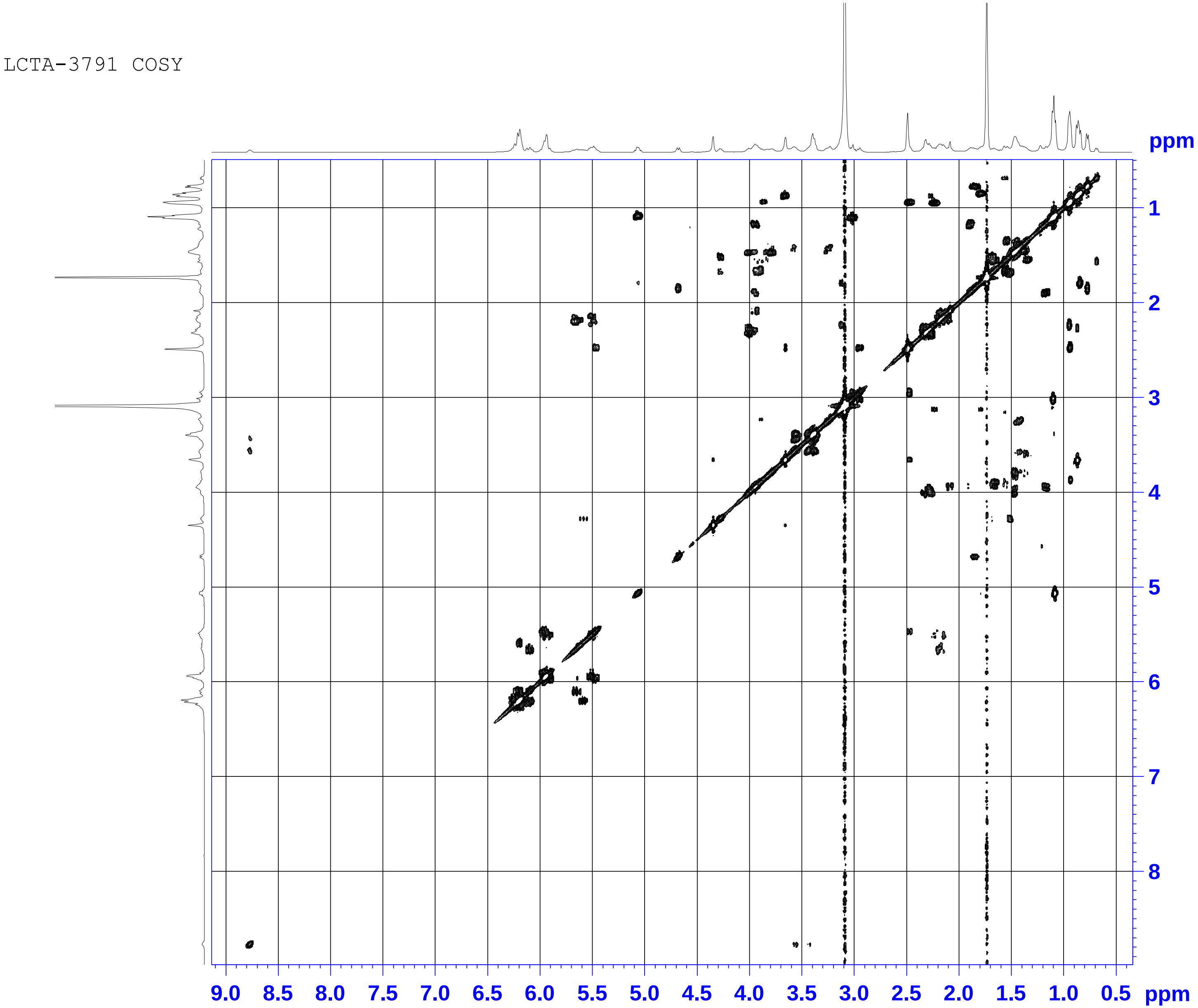

Figure S23. ^{1}H-^{1}H COSY spectra of the Nys derivative **5c**.

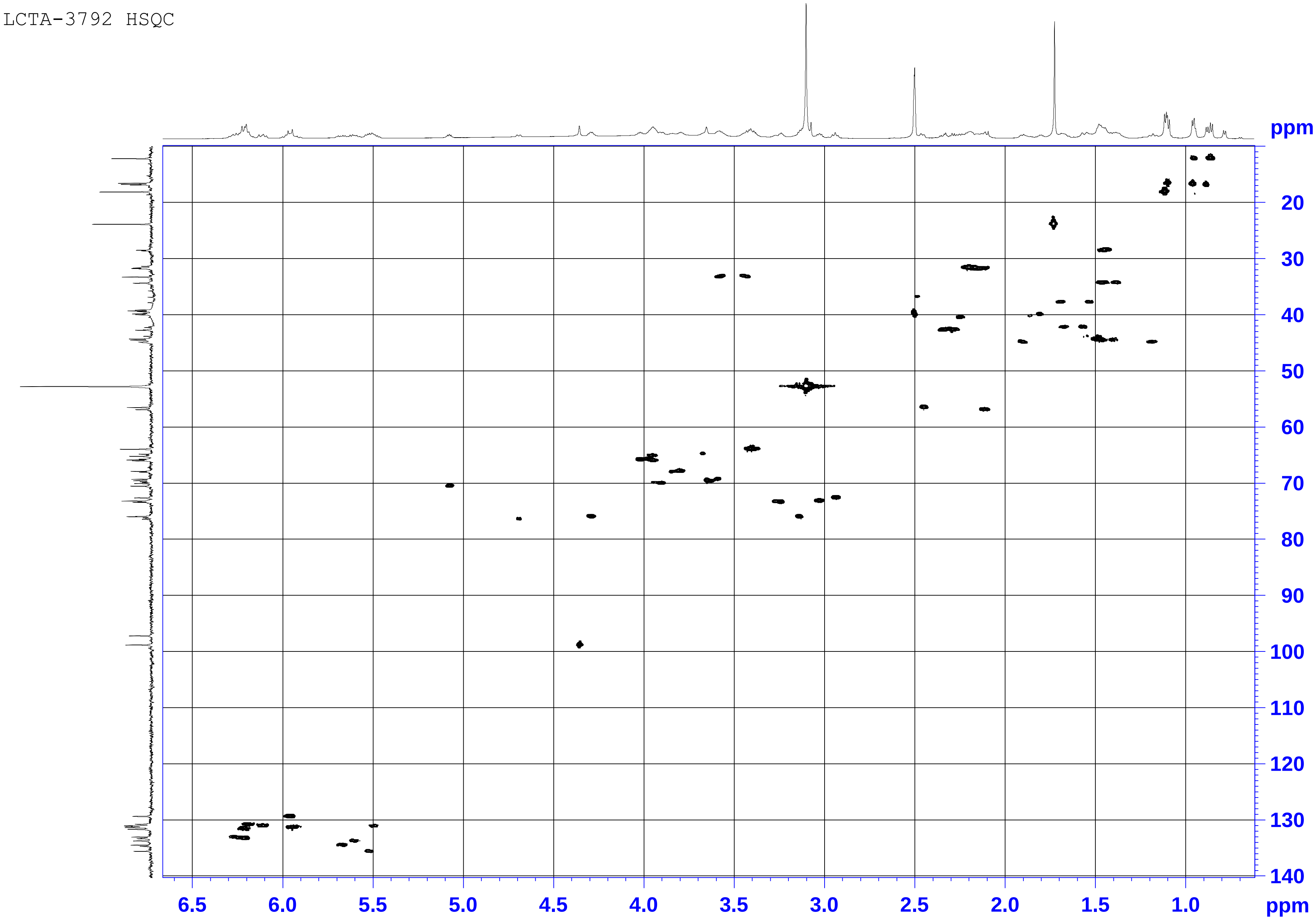

Figure S24. ^{1}H-^{13}C HSQC NMR spectra of the Nys derivative **5c**.

LCTA-3652

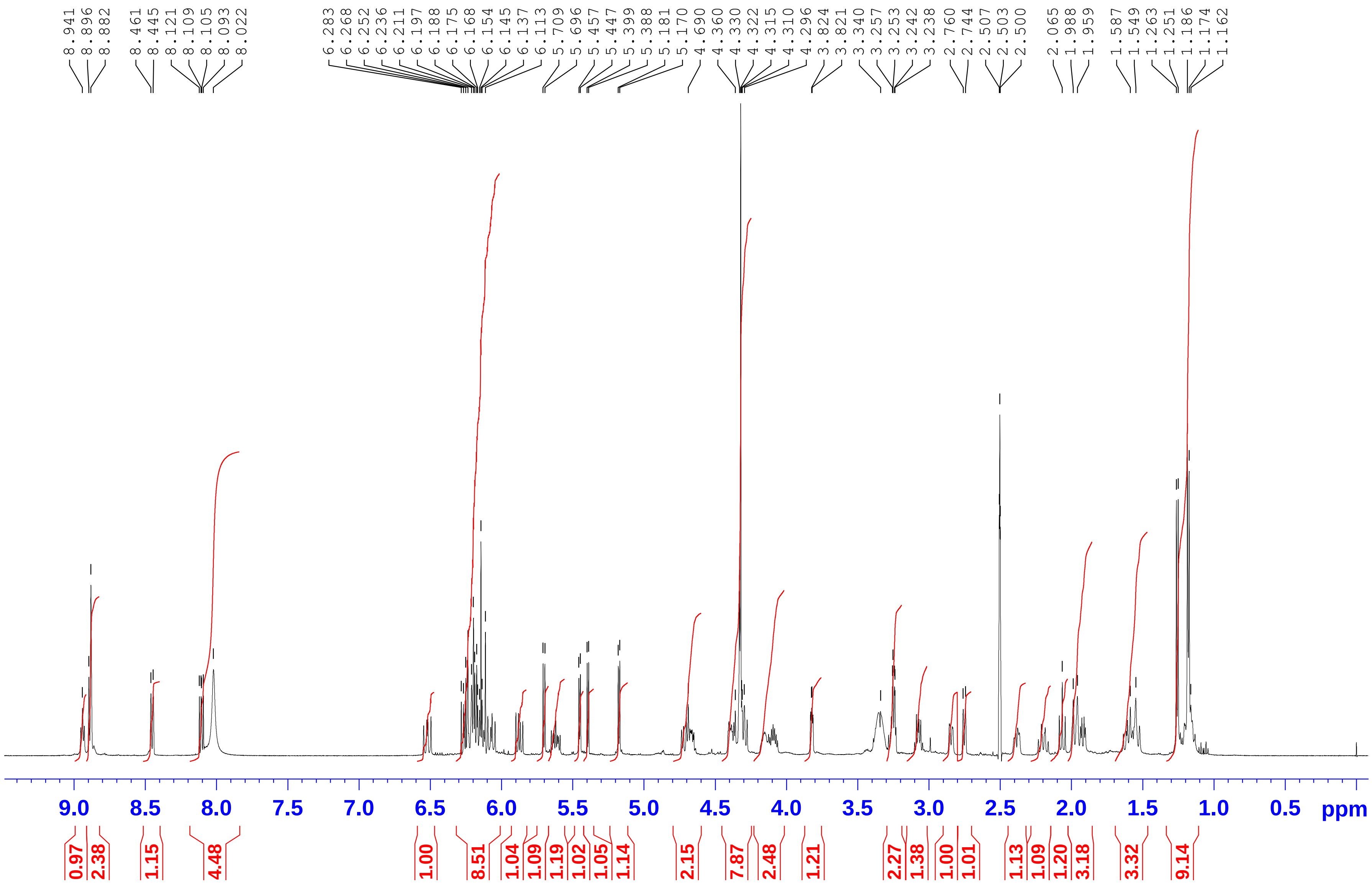

Figure S25. ^{1}H NMR spectra of the Nata derivative **6a**.

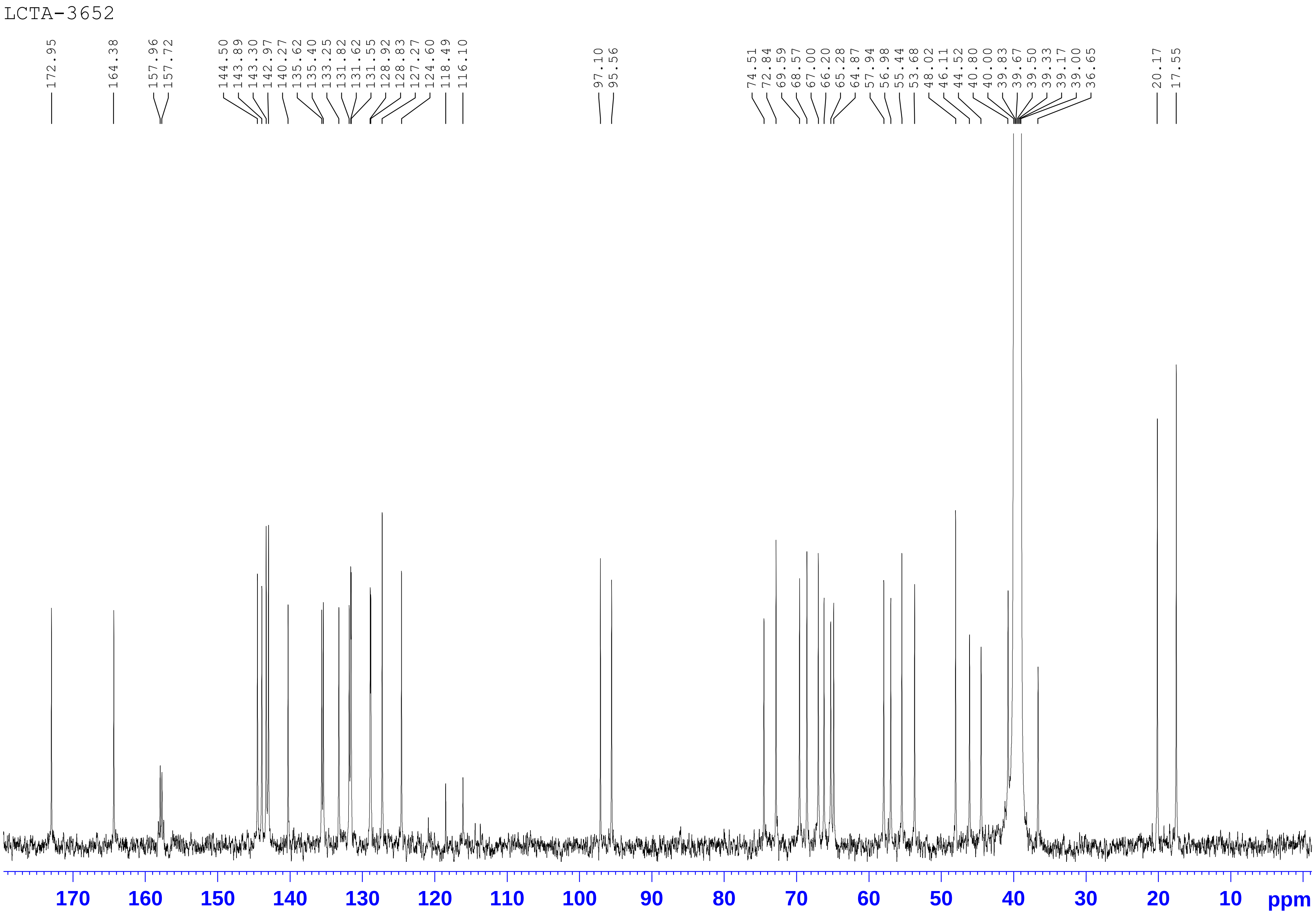

Figure S26. ^{13}C NMR spectra of the Nata derivative **6a**.

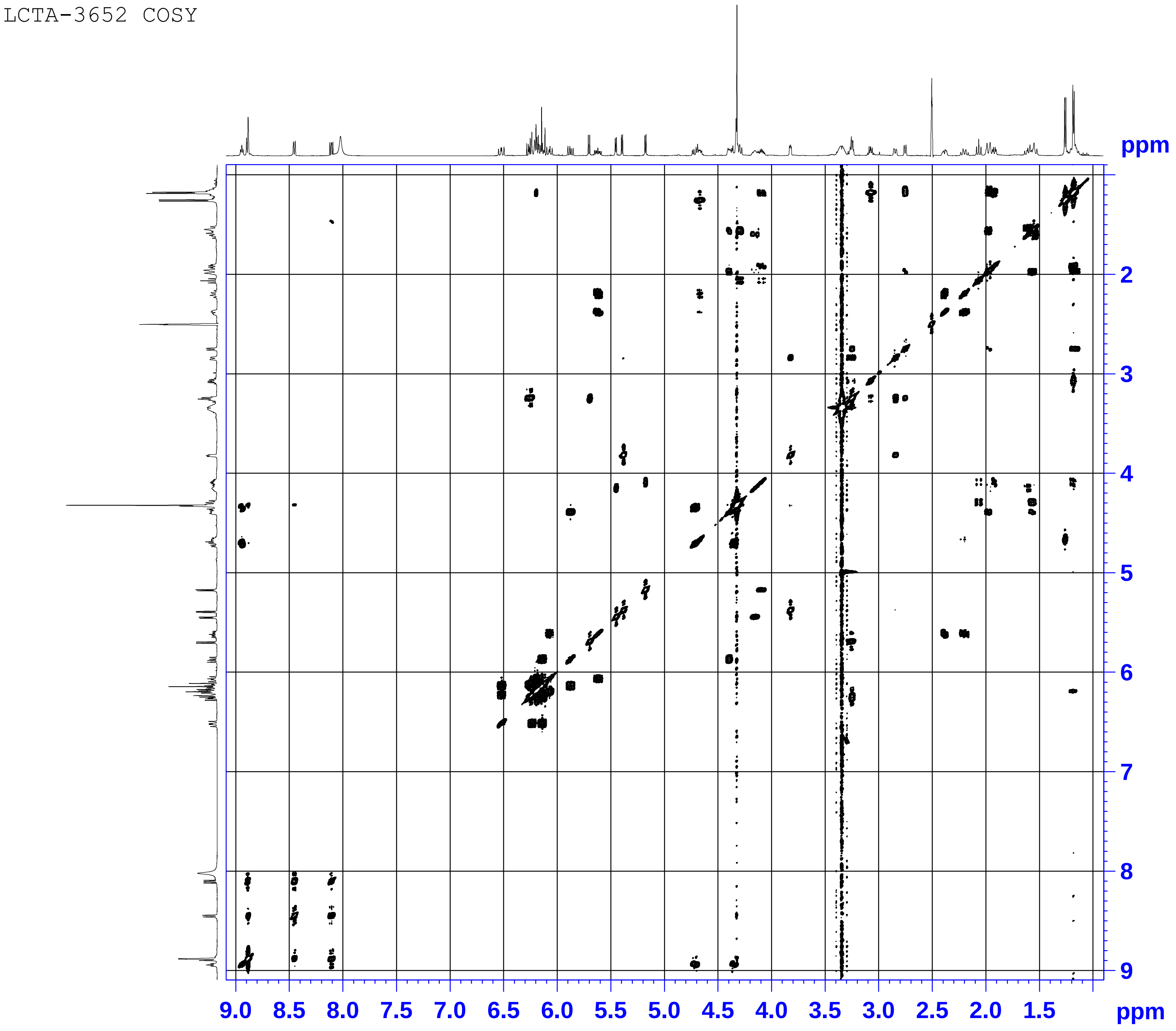

Figure S27. ^1H-^1H COSY spectra of the Nata derivative **6a**.

LCTA-3652 HSQC.

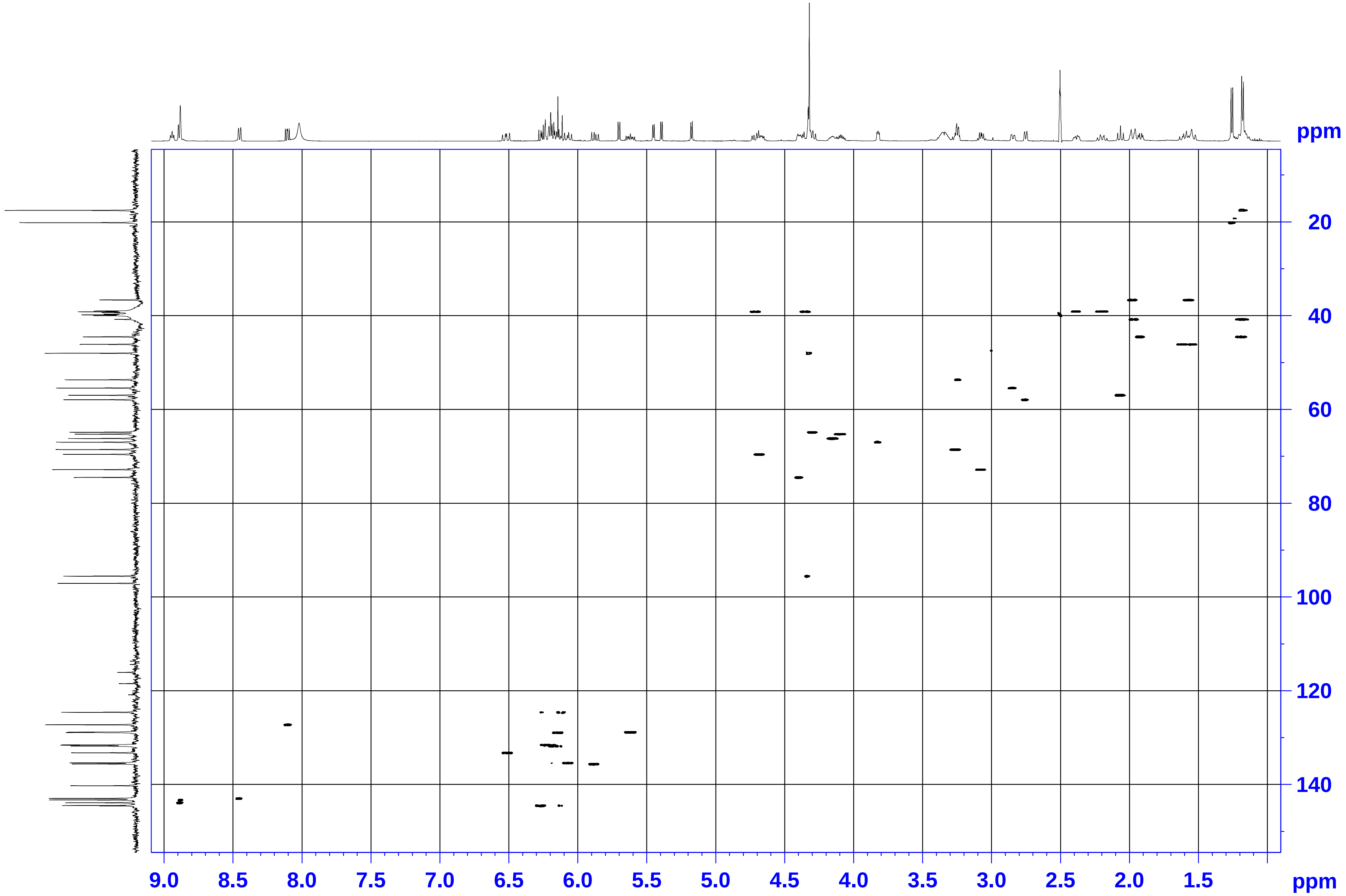

Figure S28. ^{1}H-^{13}C HSQC NMR spectra of the Nata derivative **6a**.

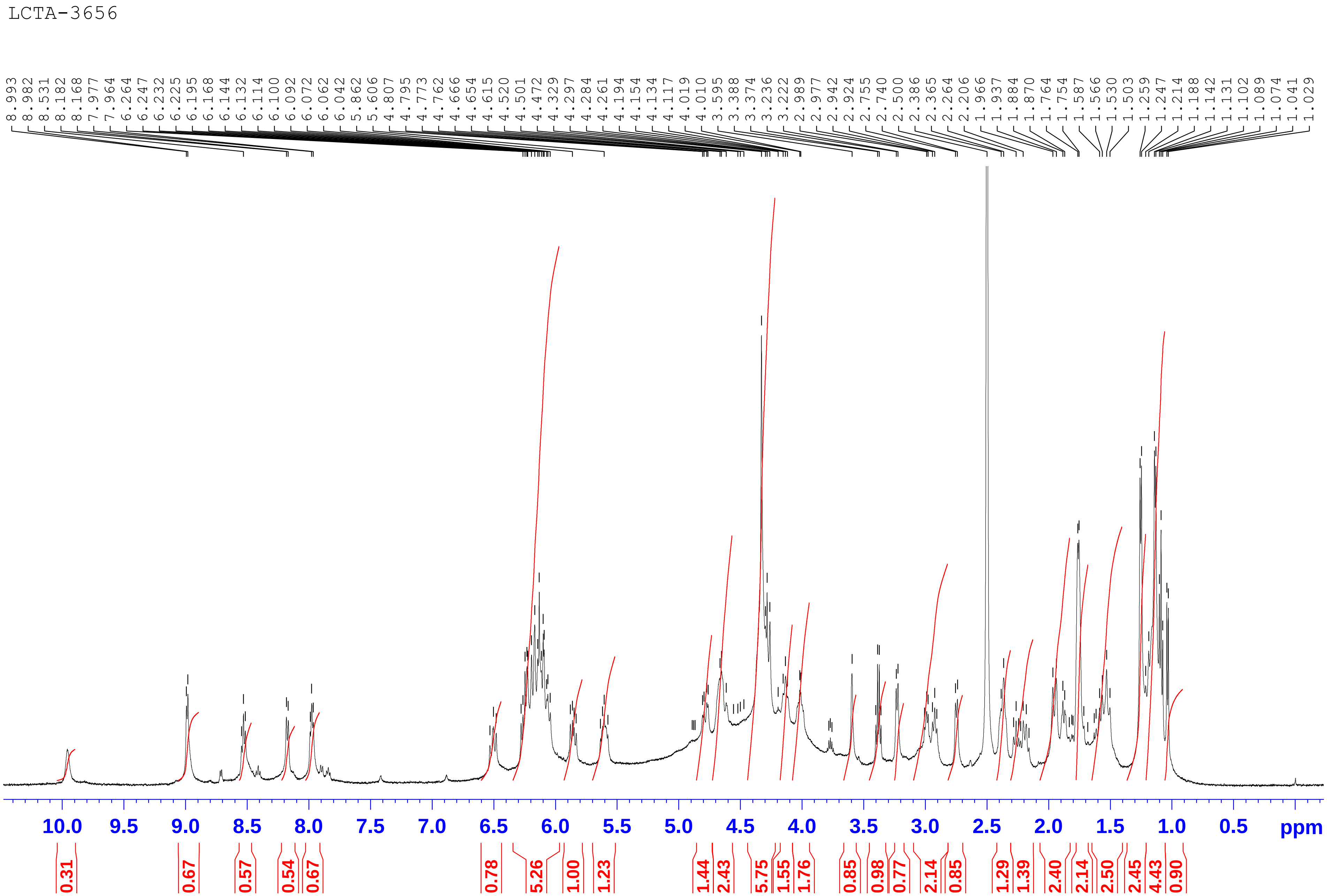

Figure S29. ^{1}H NMR spectra of the Nata derivative **6b**.

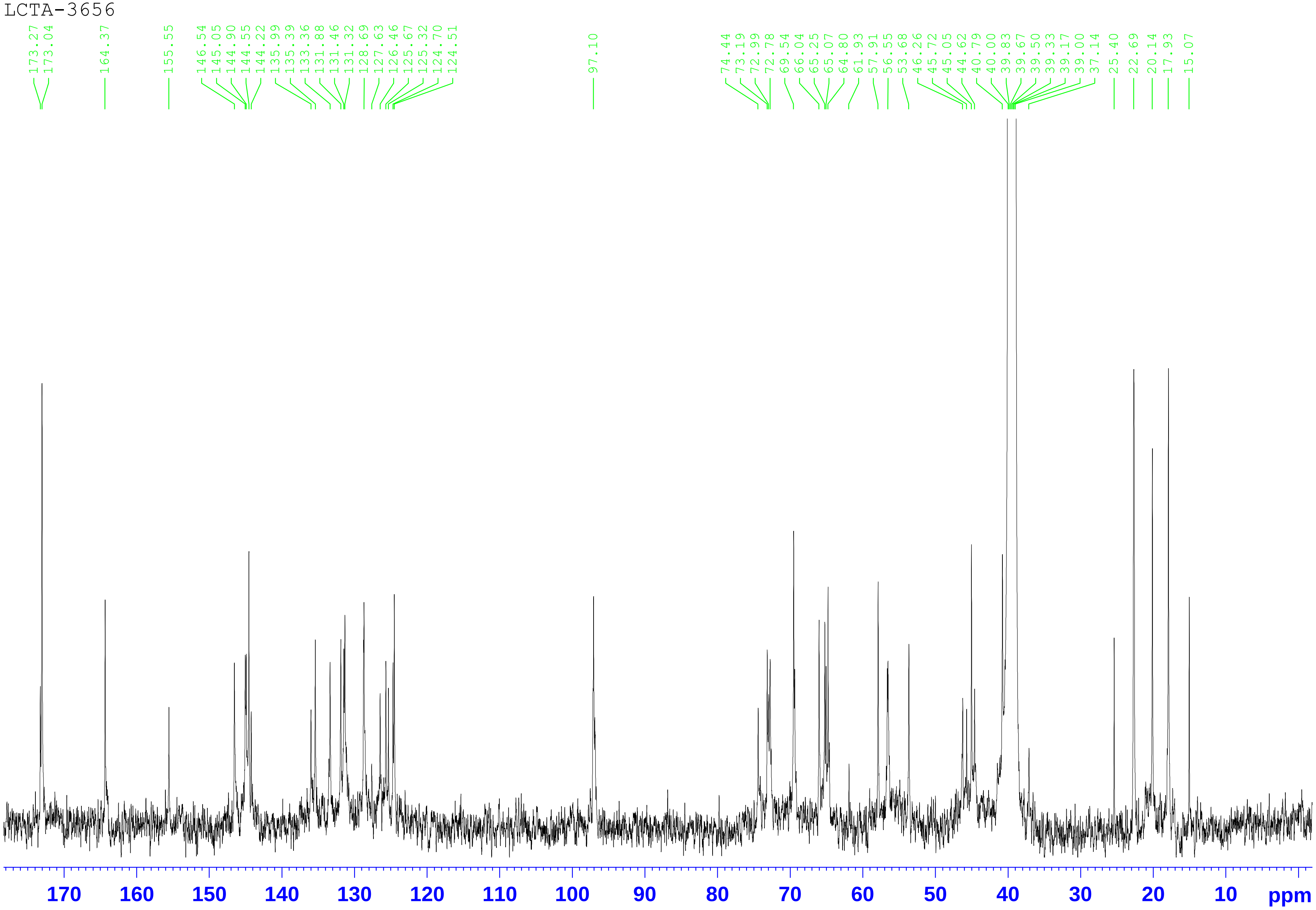

Figure S30. ^{13}C NMR spectra of the Nata derivative **6b**.

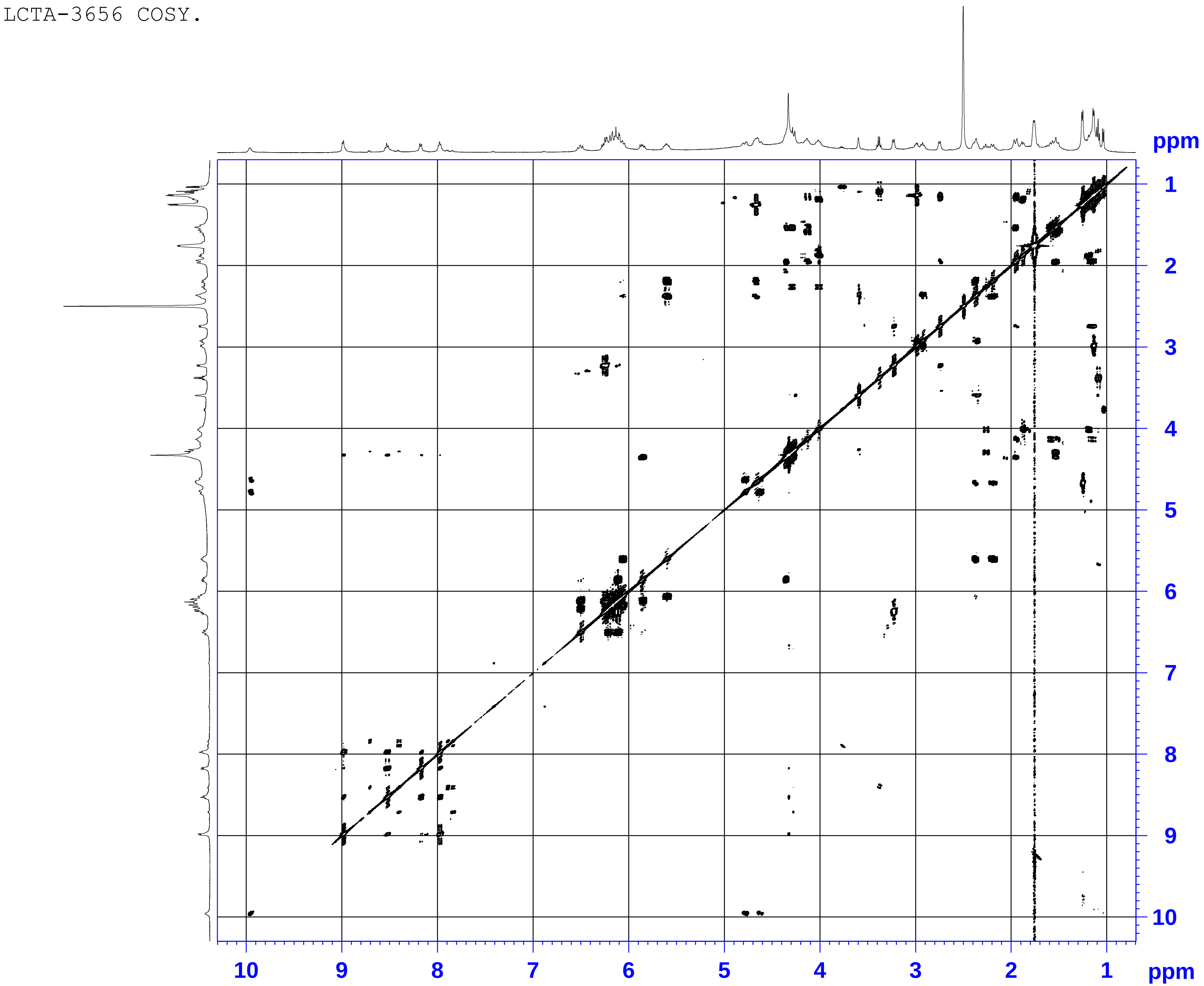

Figure S31. ^1H-^1H COSY spectra of the Nata derivative **6b**.

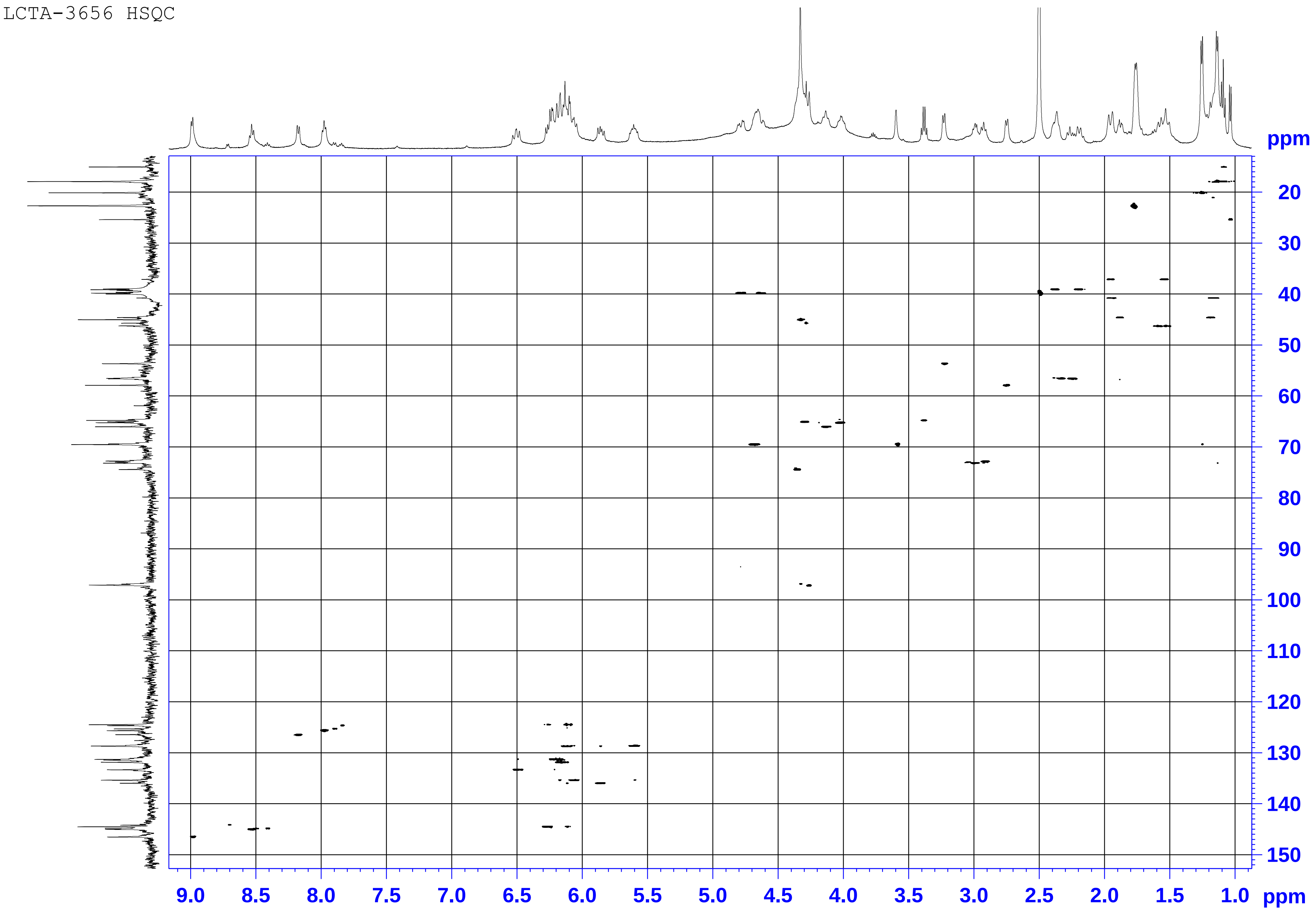

Figure S32. ^{1}H -^{13}C HSQC NMR spectra of the Nata derivative **6b**.

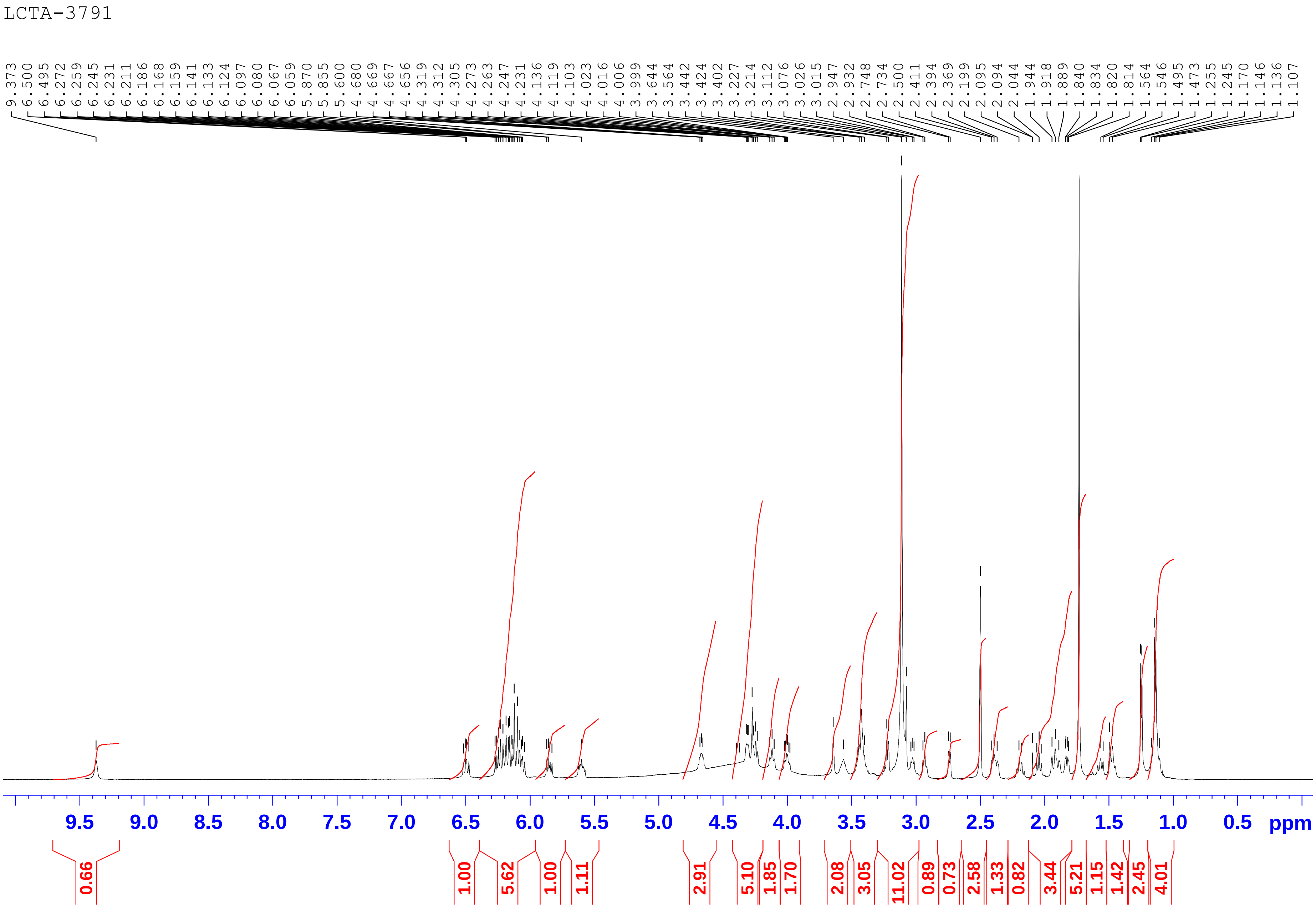

Figure S33. ^{1}H NMR spectra of the Nata derivative **6c**.

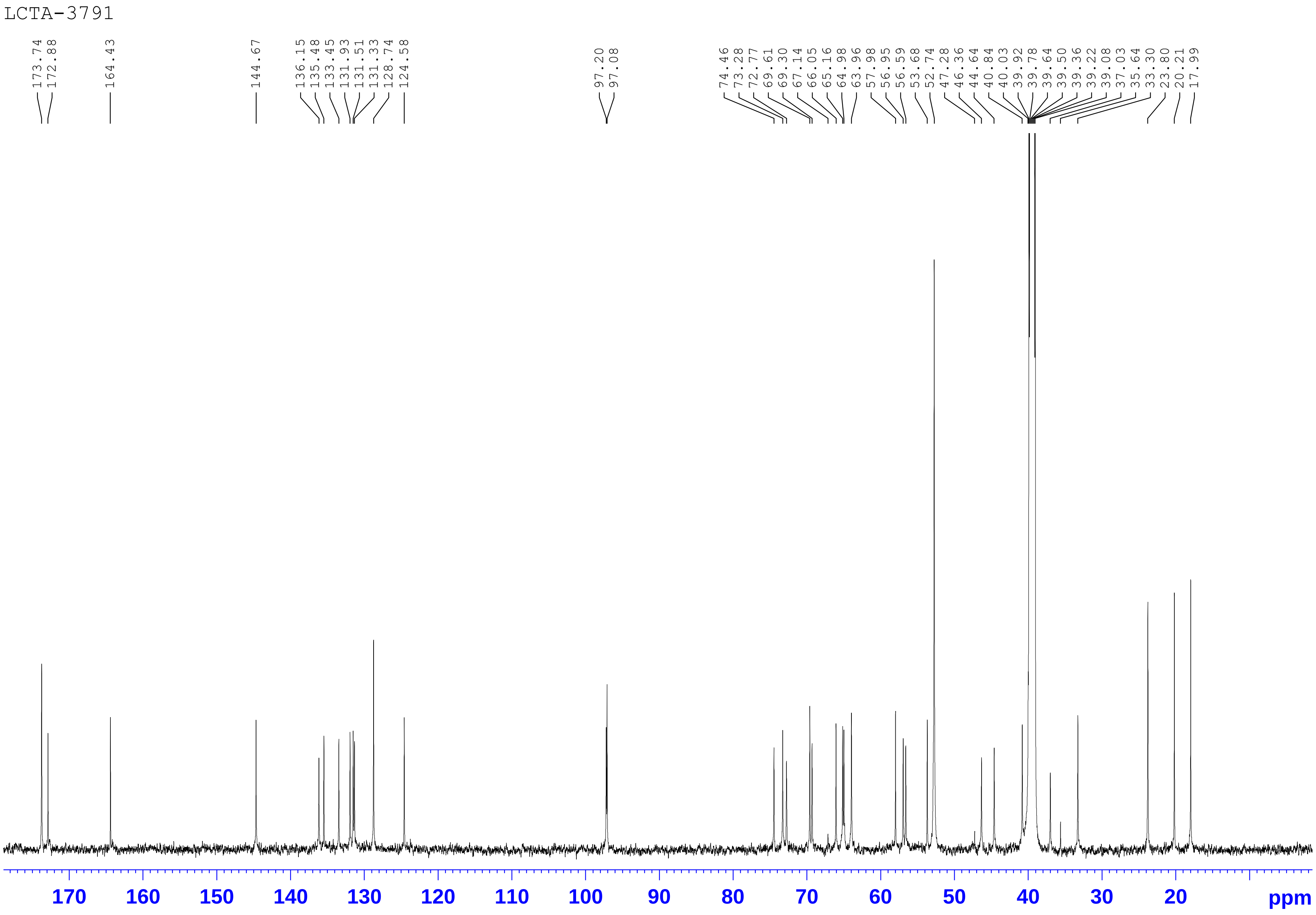

Figure S34. ^{13}C NMR spectra of the Nata derivative **6c**.

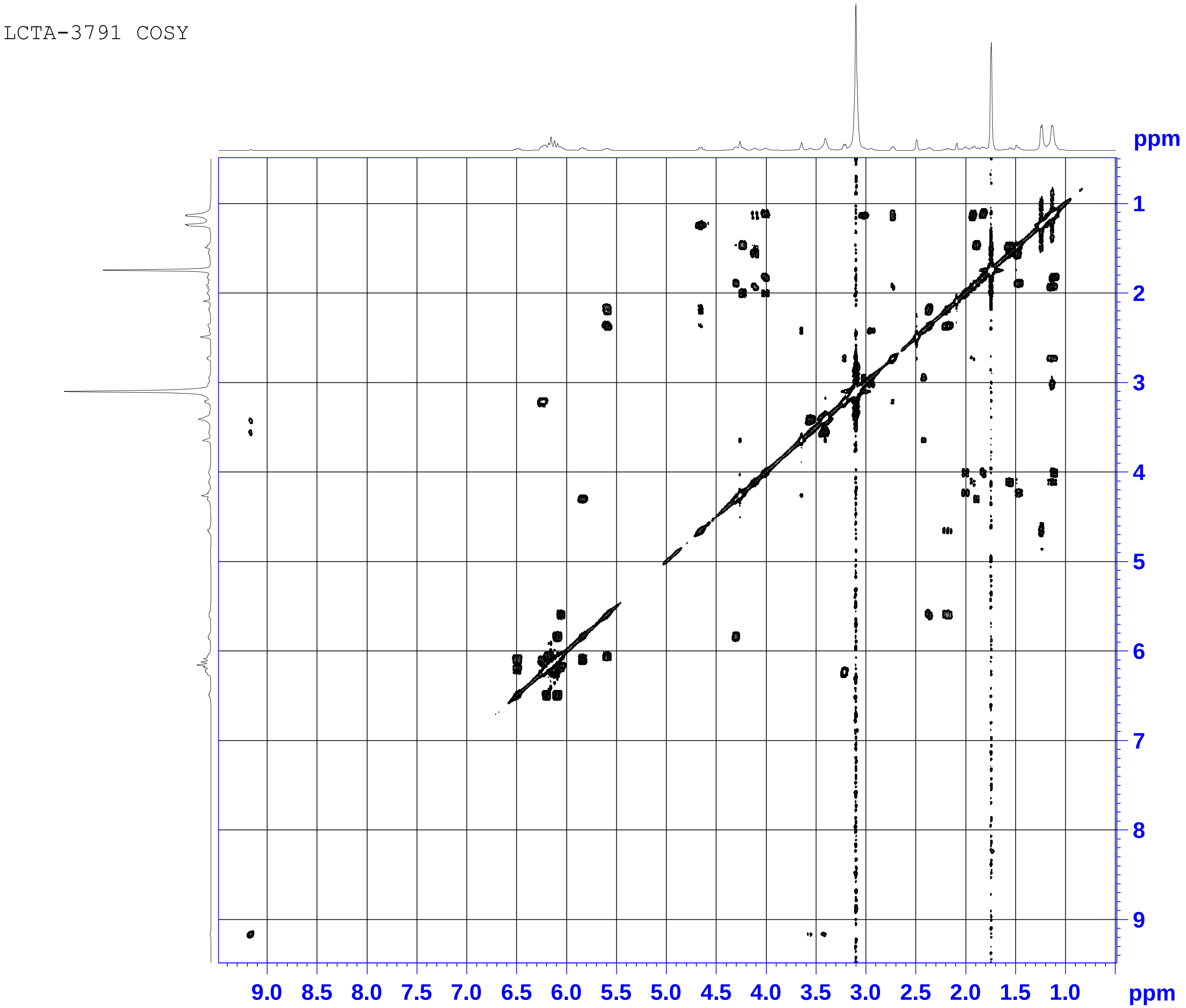

Figure S35. ^{1}H-^{1}H COSY spectra of the Nata derivative **6c**.

LCTA-3791 HSQC

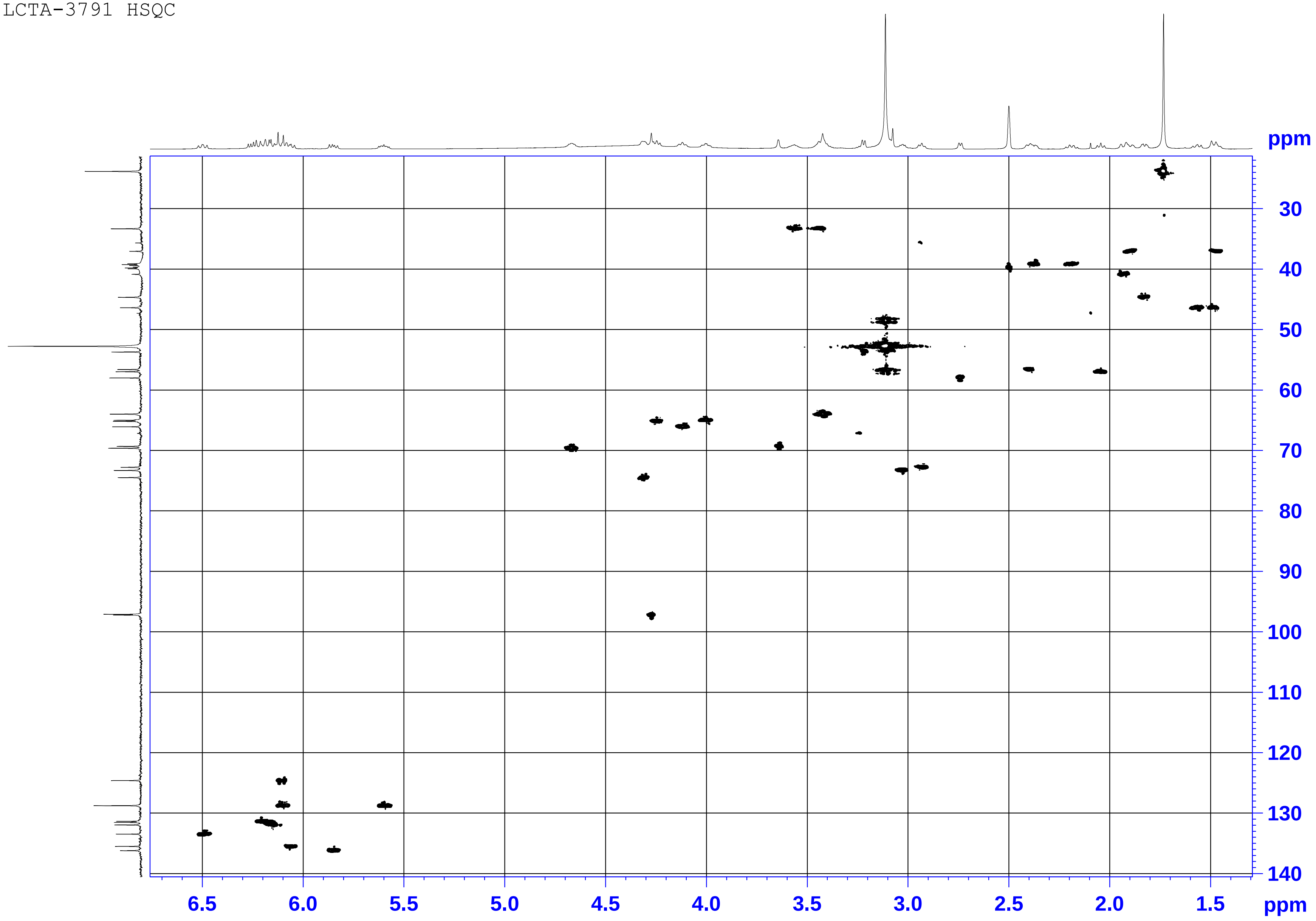

Figure S36. ^{1}H-^{13}C HSQC NMR spectra of the Nata derivative **6c**.

.
